# Supplementary material for: Magnitude of Mycobacterium tuberculosis, drug resistance and associated factors among presumptive tuberculosis patients at St. Paul’s Hospital Millennium Medical College, Addis Ababa, Ethiopia
Source: PLoS One. 2022 Aug 1;17(8):e0272459. doi: 10.1371/journal.pone.0272459 (PMC9342779; doi:10.1371/journal.pone.0272459)
Supplement: S1 File — (DOCX) [file pone.0272459.s001.docx]

## English version of the questionnaire

**Name of data collector** __________________ **Date** __________**Code of the client**_________

| **I** | **Demographic Data** |  | |
| --- | --- | --- | --- |
|  | **Questions** | **Response** | |
| 1.2 | Age of Respondent |  | |
| 1.3 | Sex | 1. Male 2. Female | |
| 1.4 | Marital status | 1. Single 2. Married 3. Divorce 4. Widowed | |
| 1.5 | Resident | 1. Urban 2. Rural | |
| 1.6 | Previous treatment for TB | 1. Yes 2. No | |
| 1.7 | Contact with TB patient | 1. Yes 2. No 3. Unknown | |
| 1.8 | Past BCG vaccination | 1. Yes 2. No | |
| 1.9 | Family number (overcrowding) | _____________________ | |
| 1.10 | Monthly income | _____________________ | |
| **II.** | **Clinical Data** | 1. Fever. | 1. Yes B. No |
|  |  | 1. Weight loss | A Yes B. No |
|  |  | 1. Cough | 1. Yes B. No |
|  |  | 1. Chest pain | 1. Yes B. No |
|  |  | 1. External adenopathy | 1. Yes B. No |
|  |  | 1. Diarrhea | 1. Yes B. No |
|  |  | 1. Dyspnea | 1. Yes B No |
|  |  | 1. Radiological signs | A. Interstitial  B. Thoracic lymphadenopathy  C. Bronchiectasis  D. Cavity  E. Bilateral  F. Unilateral |
|  |  | 1. Treatment history with anti-TB drugs | 1. Previously treated. 2. Previously untreated |
|  |  | 1. Treatment of anti HIV drug | 1. Pre treatment 2. On treatment |
|  |  | 1. Duration of treatment time | __________________ |
|  |  | 1. Type of the current HIV Drug | ______________________ |
|  |  | 1. Presumptive DRTB | 1. New 2. Relapse 3. Failure 4. Lost to follow-up 5. MDR-contact |
|  |  | 1. Site of TB | 1. Pulmonary. 2. Extra-pulmonary |
|  |  | 1. Type of specimen | 1. Respiratory (sputum). 2. Non-respiratory 3. Lymph node aspirate. 4. Pus 5. Pleural fluids 6. Others |
|  |  | 1. CD4 count | ____________/mm3 |
|  |  | 1. Viral Load | ______________/mm3 |
| **III** | **Laboratory Data** | - 1. Gen x-pert TB | 1. Detected 2. Not detected |
| 3.1 | If Q3.1 result not detected, **culture** | 1. Culture Positive 2. Culture Negative | |
|  | If Gen x-pert result positive: | 1. Resistance pattern TB 2. Non-Resistance TB | |
|  | If Q 3.1 Culture result positive, | 1. Confirmed by ZN 2. Not-confirmed by ZN | |
|  | If Q 3.1 Culture result positive, | 1. Growth on BAP 2. Not Growth on BAP | |
|  | If Q 3.1 positive, done drug susceptibility test | 1. Drug Resistant on MGIT 2. Non Resistance on MGIT | |

Thank you

## Local Language version of the questionnaire

**እዝል 3- ቃለ መጠይቅ**

**የጠያቂ ስም ______________ቀን:_______ ፊርማ________የተሳታፊ መለያ ቁጥር_____**

| **I** | **ክፍል 1: የተሳታፊው ማህበራዊ ነክመረጃዎች** | | |
| --- | --- | --- | --- |
|  | **ጥያቄዎች** | **መልሶቻቸው** | |
| 1.2 | እድሜ |  | |
| 1.3 | ጾታ | 1. ወንድ 2. ሴት | |
| 1.4 | የጋብቻ ሁኔታ? | 1. ያላገባ 2. ያገባ 3. የተፋታ 4. የሞተበት | |
| 1.5 | አድራሻ ? | 1. ከተማ 2. ገጠር | |
| 1.6 | ከጥናቱ በፊት የነቀርሳ መድሃኒት ወስደዋል? | 1. አዎ፣ ወስጃለሁ 2. የለም፣ አልወሰድኩም | |
| 1.7 | ከነቀርሳ ታማሚ ጋር ንክኪ አሉዎት ? | 1. አዎ 2. የለም 3. አይታወቅም | |
| 1.8 | ሥለ ነቀርሳ ተከትበዋል? | 1. አዎ 2. የለም | |
| 1.9 | የቤተሰብ ቁጥር ብዛት ? | _____________________ | |
| 1.10 | የወር ገቢዎ? | _____________________ | |
| **II.** | **የጤና ምርመራ መረጃ** | 1. ትኩሳት አለዎት? | 1. አዎ 2. የለም |
|  |  | 1. ክብደት መቀነስ | 1. አዎ 2. የለም |
|  |  | 1. ሳል አለዎት? | 1. አዎ 2. የለም |
|  |  | 1. የደረት ህመምስ | 1. አዎ 2. የለም |
|  |  | 1. ከሳንባ ውጭ ያለ አካል ዕብጠት ና ማቃጠል | 1. አዎ 2. የለም |
|  |  | 1. ተቅማጥ አለዎት? | 1. አዎ 2. የለም |
|  |  | 1. ቶሎቶሎ መተንፈስ | 1. አዎ 2. የለም |
|  |  | 1. የራጅ ምርመራ ምልክቶች | 1. አንጀት 2. ደረት ላይ እብጠት ና ማቃጠል 3. ሳንባን ማቃጠል 4. ባይላተራል የራጅ ምልክት 5. ዩኒላተራል የራጅ ምልክት |
|  |  | 1. የፀረ-ቲቢ መድኃኒት አጠቃቀም ታሪክ | 1. ካሁን በፊት ወስጃለሁ 2. እስካሁን አልወሰድኩም |
|  |  | 1. የፀረ-ኤች አይ ቪ መድኃኒት አጠቃቀም ታሪክ | 1. ወስጀ አላውቅም 2. እየወሰድኩ ነው |
|  |  | 1. የፀረ-ኤች አይ ቪ መድሃኒቱን እየወሰዱ ከሆነ ለምን ያህል ጊዜ ቆዩ | __________________ |
|  |  | 1. የሚወስዱት ፀረ-ኤች አይ ቪ መድሃኒት አይነት | ___________________ |
|  |  | 1. መድሃኒቱን የተላመደ ነቀርሳ በሽታ የመሆን ዕድል | 1. አዲስ ታካሚ 2. በድጋሜ ያገረሸ 3. ስኬት አልባ 4. ክትትል ማቋረጥ 5. መድሃኒቱነ የተላመደ ነቀርሳ ጋር ንክኪ |
|  |  | 1. በሽታው የሚገኝበት አካል | 1. ሣንባ ውስጥ 2. ከሣንባ ውጭ |
|  |  | 1. የናሙናው አይነት | 1. አክታ 2. ከመተንፈሻ አካል ውጭ 3. ከእባጭ አካባቢ የተወሰደ 4. ፈሳሺ 5. መግል 6. የሳንባ ፍሳሺ 7. ሌላ |
|  |  | 1. የሲዲ4 ቁጥር | ____________/ሚሜ^3^ |
|  |  | 1. ቫይራል ሎድ | ______________/ሚሜ^3^ |
| **III** | **የላቦራቶሪ ውጤት** | - 1. ጅን ኤክስፐርት | 1. ተቢ የተገኘ/የታወቀ 2. ቲቢ ያልተገኘ/ያልታወቀ |
|  | በጅን ኤክስፐርት ውጤት ከሌለው | 1. ካልቸር ላይ የታወቀ 2. በካለቸር ያልታወቀ | |
|  | 3.1 ጅን ኤክስፐርት ውጤት ካለው፣ | 1. መድሃኒቱን የተላመደ ነወ 2. መድሃኒቱን ያልተላመደ ነው | |
|  | 3.1 ካልቸር ላይ ከታወቀ | 1. በ ZN የተረጋገጠ 2. በ ZN ያልተረጋገጠ | |
|  | 3.1 ካልቸር ላይ ካልታወቀ | 1. በብለድ አጋር ፕሌት ላይ ያደገ 2. በብለድ አጋር ፕሌት ላይ ያላደገ | |
|  | 3.1 ካልቸሩ ውጤት ያለው ከሆነ፤ መድሃኒቱ ፍቱን መሆኑን መስራት | 1. መድሃኒቱን የተላመደ ቲቢ በ MGIT 2. መድሃኒቱን ያልተላመደ ቲቢ በ MGIT | |

አመሰግናለሁ
