# Supplementary material for: Magnitude of Mycobacterium tuberculosis, drug resistance and associated factors among presumptive tuberculosis patients at St. Paul’s Hospital Millennium Medical College, Addis Ababa, Ethiopia
Source: PLoS One. 2022 Aug 1;17(8):e0272459. doi: 10.1371/journal.pone.0272459 (PMC9342779; doi:10.1371/journal.pone.0272459)
Supplement: Attachment — Submitted filename: PONE-D-22-03876_ suggesions and comnts.pdf [file pone.0272459.s002.pdf]

PLOS ONE

Magnitude of Multidrug Resistance Mycobacterium tuberculosis and associated factors among presumptive patients at St. Paul’s Hospital Millennium Medical College, Addis Ababa, Ethiopia

--Manuscript Draft--

**Manuscript Number:**	PONE-D-22-03876
**Article Type:**	Research Article
**Full Title:**	Magnitude of Multidrug Resistance Mycobacterium tuberculosis and associated factors among presumptive patients at St. Paul’s Hospital Millennium Medical College, Addis Ababa, Ethiopia
**Short Title:**	Multidrug Resistance Mycobacterium tuberculosis
**Corresponding Author:**	Addisu Gize Yeshanew, M.Sc<br>St. Paul's Hospital Millennium Medical College<br>Addis Ababa, Ethiopia, ETHIOPIA
**Keywords:**	M. tuberculosis; X-Pert; Lowenstein-Jensen (LJ); Associated Factors; Addis Ababa
**Abstract:**	Background : Mycobacterium tuberculosis (M. tuberculosis) remains one of the most significant causes of death and a major public health problem in the community. Therefore, the goal of this study was to determine magnitude of Multi Drug Mycobacterium tuberculosis (MDR-TB) and its associated factors among TB-presumptive patients at St. Paul’s Hospital Millennium Medical College, Addis Ababa, Ethiopia.<br>Methods: A cross-sectional study was conducted at SPHMMC, Addis Ababa, Ethiopia from Jan to July 2019. Socio-demographic data were collected by using structured questionnaire in face to face interview with patients. Sputum samples were collected and the laboratory analysis was done by microscopy and X-pert MTB/RIF assay and LJ culture media. Data were analyzed by SPSS version 23. Binary logistic regression was done to identify the associated risk factors, and p-value less than 0.05 was taken as significant association.<br>Results: Of the total of 436 respondents, 223 (51%) were male. The mean ±SD age the participants were 38±17years. Out of the total participants, the overall confirmed Mycobacterium tuberculosis was through X-pert MTB/RIF assay and LJ culture media was 27 (6.2%), and three isolates were resistant for either INH or RIF drug, while two of them were MDR-TB based on line probe assays method. Previous TB-contact history, patient weight loss, having pneumonia with chest X-ray finding, and CD4 + T-cells count 200-350/mm 3 of blood were significantly associated predictors for MTB infection.<br>Conclusion: The magnitude of M. tuberculosis and MDR-TB in this study highlights the need for further extended early case detection and managing MDR-TB cases to minimize transmission and the suffering of patients.
**Order of Authors:**	Melkayehu Kassa
	Kassu Desta
	Rozina Ambachew
	Zenebe Youhannes
	Alganesh Yohannes
	Nuhamen Zena
	Misikir Amare
	Betselot Zerihun
	Melaku Getu
	Addisu Gize, M.Sc
**Additional Information:**	

Powered by Editorial Manager® and ProduXion Manager® from Aries Systems Corporation

Question	Response
**Financial Disclosure**<br><br>Enter a financial disclosure statement that describes the sources of funding for the work included in this submission. Review the submission guidelines for detailed requirements. View published research articles from *PLOS ONE* for specific examples.<br><br>This statement is required for submission and **will appear in the published article** if the submission is accepted. Please make sure it is accurate.<br><br>**Unfunded studies**<br>Enter: *The author(s) received no specific funding for this work.*<br><br>**Funded studies**<br>Enter a statement with the following details:<br>• Initials of the authors who received each award<br>• Grant numbers awarded to each author<br>• The full name of each funder<br>• URL of each funder website<br>• Did the sponsors or funders play any role in the study design, data collection and analysis, decision to publish, or preparation of the manuscript?<br>• **NO** - Include this sentence at the end of your statement: *The funders had no role in study design, data collection and analysis, decision to publish, or preparation of the manuscript.*<br>• **YES** - Specify the role(s) played.<br><br>* typeset	This research work was supported by Addis Ababa University, Ethiopia.<br>The funders had no role in study design, data collection and analysis, decision to publish, or preparation of the manuscript.
**Competing Interests**<br><br>Use the instructions below to enter a competing interest statement for this submission. On behalf of all authors, disclose any competing interests that could be perceived to bias this work—acknowledging all financial support and any other relevant financial or non-financial competing interests.	The authors have declared that no competing interests exist.

This statement is required for submission and **will appear in the published article** if the submission is accepted. Please make sure it is accurate and that any funding sources listed in your Funding Information later in the submission form are also declared in your Financial Disclosure statement.<br><br>View published research articles from *PLOS ONE* for specific examples.<br><br>**NO authors have competing interests**<br><br>Enter: *The authors have declared that no competing interests exist.*<br><br>**Authors with competing interests**<br><br>Enter competing interest details beginning with this statement:<br><br>*I have read the journal's policy and the authors of this manuscript have the following competing interests: [insert competing interests here]*<br><br>* typeset	
**Ethics Statement**<br><br>Enter an ethics statement for this submission. This statement is required if the study involved:<br><br>• Human participants<br>• Human specimens or tissue<br>• Vertebrate animals or cephalopods<br>• Vertebrate embryos or tissues<br>• Field research<br><br>Write "N/A" if the submission does not require an ethics statement.<br><br>General guidance is provided below. Consult the submission guidelines for detailed instructions. **Make sure that all information entered here is included in the Methods section of the manuscript.**	The proposed study was approved by the Department of Medical Laboratory Science, Addis Ababa university research and ethics committee concerning the ethical issues giving a reference number SR/LS/025/19.

**Format for specific study types**<br><br>**Human Subject Research (involving human participants and/or tissue)**<br>• Give the name of the institutional review board or ethics committee that approved the study<br>• Include the approval number and/or a statement indicating approval of this research<br>• Indicate the form of consent obtained (written/oral) or the reason that consent was not obtained (e.g. the data were analyzed anonymously)<br><br>**Animal Research (involving vertebrate animals, embryos or tissues)**<br>• Provide the name of the Institutional Animal Care and Use Committee (IACUC) or other relevant ethics board that reviewed the study protocol, and indicate whether they approved this research or granted a formal waiver of ethical approval<br>• Include an approval number if one was obtained<br>• If the study involved *non-human primates*, add *additional details* about animal welfare and steps taken to ameliorate suffering<br>• If anesthesia, euthanasia, or any kind of animal sacrifice is part of the study, include briefly which substances and/or methods were applied<br><br>**Field Research**<br><br>Include the following details if this study involves the collection of plant, animal, or other materials from a natural setting:<br>• Field permit number<br>• Name of the institution or relevant body that granted permission	
**Data Availability**<br><br>Authors are required to make all data underlying the findings described fully available, without restriction, and from the time of publication. PLOS allows rare exceptions to address legal and ethical concerns. See the PLOS Data Policy and FAQ for detailed information.	Yes - all data are fully available without restriction

Powered by Editorial Manager® and ProduXion Manager® from Aries Systems Corporation

A Data Availability Statement describing where the data can be found is required at submission. Your answers to this question constitute the Data Availability Statement and **will be published in the article**, if accepted.<br><br>**Important:** Stating 'data available on request from the author' is not sufficient. If your data are only available upon request, select 'No' for the first question and explain your exceptional situation in the text box.<br><br>Do the authors confirm that all data underlying the findings described in their manuscript are fully available without restriction?	
**Describe where the data may be found in full sentences. If you are copying our sample text, replace any instances of XXX with the appropriate details.**<br><br>• If the data are **held or will be held in a public repository**, include URLs, accession numbers or DOIs. If this information will only be available after acceptance, indicate this by ticking the box below. For example: *All XXX files are available from the XXX database (accession number(s) XXX, XXX.).*<br>• If the data are all contained **within the manuscript and/or Supporting Information files**, enter the following: *All relevant data are within the manuscript and its Supporting Information files.*<br>• If neither of these applies but you are able to provide **details of access elsewhere**, with or without limitations, please do so. For example:<br><br>*Data cannot be shared publicly because of [XXX]. Data are available from the XXX Institutional Data Access / Ethics Committee (contact via XXX) for researchers who meet the criteria for access to confidential data.*<br><br>*The data underlying the results presented in the study are available*	All data relevant to this study are available on the manuscript.

from (include the name of the third party and contact information or URL).<br>• This text is appropriate if the data are owned by a third party and authors do not have permission to share the data.<br><br>* typeset	
Additional data availability information:	

Powered by Editorial Manager® and ProduXion Manager® from Aries Systems Corporation

Manuscript
Click here to access/download;Manuscript;Ko Manuscript-all_PLOS ONE.docx

Magnitude of Multidrug Resistance *Mycobacterium tuberculosis* and associated factors among presumptive patients at St. Paul's Hospital Millennium Medical College, Addis Ababa, Ethiopia

Melkayehu Kassa[1], Kassu Desta[2], Rozina Ambachew[1], Zenebe Youhannes[1], Alganesh Yohannes[1], Nuhamen Zena[1], Misikir Amare[3], Betselot Zerihun[3], Melaku Getu[3] and Addisu Gize[1*]

[1]St.Paul's Hospital Millennium Medical College, Addis Ababa, Ethiopia.

[2]College of Health Science, Addis Ababa University, Addis Ababa, Ethiopia

[3]Ethiopian Public Health Institute, Addis Ababa, Ethiopia.

Authors' Email:

Melkayehu Kassa= kassamelkayehu@gamil.com

Kassu Desta= kassudesta2020@gmail.com

Rozina Ambachew= nanieyasu@gmail.com

Zenebe Youhannes= zenebezoha@gmail.com

Alganesh Yohannes= alganesh5838@gmail.com

Nuhamen Zena= nuhamenzf@gmail.com

Misikir Amare= misikiramare.ma@gmail.com

Betselot Zerihun= betselotzerihun3@gmail.com

Melaku Getu= melakgetu2005@gmail.com

Addisu Gize= addisu.gize@sphmmc.edu.et

*Corresponding author= Addisu Gize, Email: addisu.gize@sphmmc.edu.et , or konjoaddisu@gmail.com

[illegible]bstract

Background: *Mycobacterium tuberculosis* (*M. tuberculosis*) remains one of the most significant causes of death and a major public health problem in the community.

Therefore, the goal of this study was to determine magnitude of Multi Drug *Mycobacterium tuberculosis (*MDR-TB) and its associated factors among TB- presumptive patients at St. Paul's Hospital Millennium Medical College, Addis Ababa, Ethiopia.

Methods: A cross-sectional study was conducted at [illegible]HMMC, Addis Ababa, Ethiopia from Jan to July 2019. Socio-demographic data were collected by using structured questionnaire in face to face interview with patients. Sputum samples were collected and the laboratory analysis was done by microscopy and X-pert MTB/RIF assay and LJ culture media. Data were analyzed by SPSS version 23. Binary logistic regression was done to identify the associated risk factors, and p-value less than 0.05 was taken as significant association.

Results: Of the total of 436 respondents, 223 (51%) were male. The mean ±SD age the participants were 38±17years. Out of the total participants, the overall confirmed *Mycobacterium tuberculosis* was through X-pert MTB/RIF assay and LJ culture media was 27 (6.2%), and three [illegible]lates were resistant for either INH or RIF drug, while two of them were MDR-TB based on line probe assays method. Previous TB-contact history, patient weight loss, having pneumonia with chest X-ray finding, and CD4$^+$ T-cells count 200-350/mm^3 of blood were significantly associated predictors for MTB infection.

Conclusion: The magnitude of *M. tuberculosis* and MDR-TB in this study highlights the need for further extended early case detection and managing MDR-TB cases to minimize transmission and the suffering of patients.

Key words: *M. tuberculosis, X-Pert, Lowenstein-Jensen (LJ)***,** Associated Factors, Addis Ababa.

[illegible]ckground

Tuberculosis (TB) is an infectious disease caused by strains belonging to the *Mycobacterium tuberculosis* complex. It is transmitted by respiratory route when a patient is coughing or sneezing, and one strain of TB, Mycobacterium bovis, can be caused by drinking not boiled milk [1]. World Health Organization (WHO) estimated that 10 million people developed tuberculosis (TB) and 1.6 million died of TB globally in [illegible]7 and one-fourth of people infected with latent *Mycobacterium tuberculosis* [2].

Globally, the estimated prevalence of MDR-TB was 3.3% in newly diagnosed patients in the WHO 2015 report. This was higher to 20% in patients with a history of anti-TB treatment[illegible]).

A global TB report estimated that there were about [illegible],000 (247 per 100,000 population) incident cases of TB in Ethiopia.

Ethiopia ranks seventh among the world’s 22 high-TB-burden countries, 10th among high-TB-pandemic countries, and fourth in sub-Saharan Africa [3].

Based on the[illegible]5 nationwide survey in Ethiopia, the prevalence of MDR-TB was 1.6% among new cases and 11.8% in the retreatment cases and rifampicin resistant was lower than 2% in new cases [4].

[illegible]gh mortality rate was observed in different health institution of the Northern Ethiopia; 87 (11.3%) patients died in Mekelle Hospital and Ayder Comprehensive Hospital [5], 38 (14.02%) children from TB/HIV co-infected University of Gondar Comprehensive Specialized Hospital [6] and from [illegible]ltidrug resistant tuberculosis (MDR-TB) data showed that 61(29.47%) of the patients died in different hospitals of Amhara region, Northwest Ethiopia [7]. Generally in Ethiopia, TB mortality rate declined from 393.8/100,000 to 100/100,000 between 1990 and 2016

(with a total decline of 75%), which indicates slow decline and resulted males had higher TB mortality rate than females [8].

Sputum smear microscopy remains the most common way to diagnose pulmonary TB. Depending on the report and method used, smear microscopy can accurately detect TB in 20% to 80% (using fluorescence microscopy methods) of TB cases. However, it could be used to diagnose TB when sputum has sufficient bacillary load, and it cannot detect drug resistance. Thus, HIV-associated TB often goes undetected because people living with HIV (PLHIV), especially those with severe immunosuppression generally have very low numbers of bacilli [9]. [illegible]nce, *X-Pert* used as an initial diagnostic test for TB detection and rifampicin resistance detection in patients suspected of having TB, MDR-TB, or HIV-associated TB is sensitive and specific [10]. Therefore, the goal of this study was to determine magnitude of *Mycobacterium tuberculosis* and its associated factors among TB- presumptive patients referred to St. Paul's Hospital Millennium Medical College, Addis Ababa, Ethiopia.

Materials and methods

Study area

The study was conducted in St Paul's Hospital Millennium Medical College, Addis Ababa, Ethiopia. It is currently has 392 beds, with an annual average of 200,000 patients and a catchment population of more than 5 million. The hospital receives referrals from around the country and is under the guidance of the Ethiopian Federal Ministry of Health.

Study design and period

A cross-sectional study was conducted at SPHMMC, Addis Ababa, Ethiopia from Jan to July 2019, and all patients who visited were source population while all *[illegible]obacterium tuberculosis*

[illegible]sumptive patients visited microbiology laboratory and fulfill the inclusion criteria were considered as study population.

Inclusion and Exclusion criteria

All presumptive *Mycobacterium tuberculosis* patients visiting Microbiology laboratory were included and patients who have inadequate specimen, previous history of known multidrug [illegible]sistance for *Mycobacterium tuberculosis* were excluded from the study.

Variables

Magnitude of *Mycobacterium tuberculosis* and its drug resistance pattern among presumptive patients dependent variables. Whereas, socio-demographic characteristics (age, sex, etc), possible risk factors like; TB contact history, previous treatment for TB, presumptive DRTB, BCG vaccination status, CD4 and HIV viral load counts were independent variables.

Sample size and sampling technique

The sample size was estimated based on the assumption of single population proportion formula, considering the previous study conducted in [illegible]remarkose, Northwest Ethiopia taken as 23%, 5% marginal error, and 95% confidence level to get the highest sample size, the calculation result determined as:

$$n = \frac{(z^{\alpha}/_{2})^2 \, p\,(1-p)}{d^2} \Longrightarrow \frac{(1.96)^2 \, 0.5(1-0.5)}{(0.05)^2} = 384 \text{ study subjects.}$$

Where: n = minimum sample size,

P = estimated proportion of *Mycobacterium tuberculosis* for the study population, and taking 10% non-response rate, the final sample size become 422 participants.

d= the margin of sample error, $z^{\alpha}/_{2}=$ the standard normal variable at $1-^{\alpha}/_{2}$ confidence level and we used consecutive sampling technique was used to select the study population.

Data collection procedure

Data collectors were trained and informed how to collect the data. Structured questionnaire was used to collect the socio-demographic status and associated risk factors of the study participants. From each presumptive *Mycobacterium tuberculosis* patients, 2-4 ml of clinical sputum sample was collected.

Laboratory procedures

Microscopy and gene X-pert® MTB/RIF were done.

The Gene X-pert® MTB/RIF purifies and concentrates *M. tuberculosis* bacilli from clinical samples. Genomic material isolated from the captured bacteria by sonication and subsequently amplifies the genomic DNA by polymerase chain reaction (PCR). Furthermore, the process identifies all the clinically relevant rifampicin resistance inducing mutations in the RNA polymerase beta (rpoB) gene in the *M. tuberculosis* genome in a real time format using fluorescent probes Gene X-pert is capable of detecting rifampicin resistance in pulmonary and extra-pulmonary specimens from clinical cases of TB. The Gene X-pert can detect mutations in the rpoB gene and show the results in <2 hours, finally the results were recorded [11]. Lowenstein-Jensen (LJ) medium was used which incorporates congo red and malachite green to inhibit unwanted bacteria for culturing. Once good growth was obtained, the positive slants were stored in a cool, dark place to archive the positive *M. tuberculosis* isolates.

Data Quality Assurance

The questionnaire was pre-tested and proper training prior to the actual data collection was given for data collectors. The necessary adjustments were made after the pre-test. The quality of data was maintained through strictly following the pre-analytical, analytical and post-analytical steps.

Data analysis and interpretation

The collected data were entered to EPI info 2002 version 3.32 after data editing and cleaning it was exported to SPSS version 23 windows software computer program for analysis. The logistic regression was employed to assess the association between of d[illegible]erent factors. A p-value of less than 0.05 was considered as statistical significance.

Ethical considerations

This study was approved by Department of Medical Laboratory Sciences, College of Health Sciences, Addis Ababa University, Addis Ababa, Ethiopia giving a reference number SR/LS/025/19. Then official permission was obtained from Institutional Review Board (IRB) of St. Paul's Hospital Millennium Medical College, Addis Ababa, Ethiopia, and then submitted to laboratory department. Written informed consent was secured from each participant. Patients' names were not recorded on the questionnaire to guarantee confidentiality of the information and privacy of the patients. Infected patients and/or those who had resistance *M. tuberculosis* were informed to their health care provider for better care and management.

Results

Socio-demographic characteristics

The total of 436 respondents were included in the study, of this 223 (51%) were male. The mean ± SD age the participants were 38±17years. [illegible] highest age category was 35-49 years and the

least was less than 15 years old. Majority of the respondents were 240 (55%) [illegible]e urban resident, 214 (49%) had monthly income 100-1000 Ethiopian Birr, table 1.

Clinical data

[illegible]m the total 374 (85.8%) were suspected for pulmonary tuberculosis and 62 (14.2%) were suspected for extra-pulmonary tuberculosis, 130 (30 %) were HIV positive individuals. [illegible]ut 422 (96.8%) of the participants were presumptive TB whereas 14(3.2%) were presumptive DRTB. In this study 33(7.5%) had history of TB patient/family contact, 68(15.6%) history of alcohol drinking, 22 (5%) history of cigarettes smoking, 319 (73.1%) fever, 311 (71.3%) night sweating and 365(83.7%) had cough. Out of 130 HIV positive participants, 104 (81%) were on anti-HIV treatment and monitored their $CD4^+$ T-cells count, in addition, 119 (91.5%) participants tested for HIV viral load. Higher magnitude seems to be appeared for those who have $CD4^+$count 200-350/mm^3 (5/34) and their viral load was ≥1000/mm^3 (6/90), table 2.

[illegible]agnitude of *M. tuberculosis* and resistance pattern

Out of the total participants, [illegible]8.3%) were detected with X-pert, and of this figure only 2 (0.5 %) of them were RIF resistant. [illegible]arding culture result, 27(6.2%) was positive and one *M. tuberculosis* strain was resistant for Isozianide drug (mono-resistant) and 2 were resistant for Isozianide and RIF (Multidrug resistant TB). The bivariate logistic regression analysis of socio-[illegible]emographic characteristics showed, age of less than 15 years old has 1.8 times (95% CI: 0.4, 8.1) more likely to develop *M. tuberculosis* as compared to age greater than 50 years old, and widowed participants were 2.6 times (95% CI: 0.4, 17) more likely to have *M. tuberculosis* than single marital status, and [illegible]’t Workers were 1.8 times (95% CI: 0.6, 5.9) more likely to have *M. tuberculosis* than housewife, however, none of the socio-demographic characteristics significantly associated with[illegible]. tuberculosis, table 3.

[illegible]ivariate analysis

Presence of contact history with tuberculosis infected patients, pneumonia confirmed with chest-X-ray examination, and $CD4^{+}$results were associated factors for *M. Tuberculosis* in the bivariate logistic analysis, however, none of the factors associated in multivariable analysis, table 4.

Discussion

The highest TB frequency was observed in age groups of 35-49 years old, living in 4-6 family size / house, regarding to occupation; laborer workers, having monthly income 1001-2000 Ethiopian Birr. The TB magnitude among this productive age group (35-49) years of study participants was (9 [33.3%]). This might be due to more exposure to the high workload, and wide range of mobility in these age-groups.

In this study it seems that as the number of family size per house hold increase the prevalence of smear positivity also increases. Family size 5-6 was highly affected by *Mycobacterium tuberculosis*. Different studies indicated individuals living in larger family size members and malnutrition are at higher risk of developing pulmonary tuberculosis [12], however our study revealed that no association family size/house hold and *Mycobacterium tuberculosis*.

Higher *Mycobacterium tuberculosis* was detected from participants diagnosed the reason for presumptive tuberculosis 25/436 (5.7%), from non-vaccinated for BCG 18/436 (4.1%), in non-alcoholic drinkers 21/436 (4.8%), and non-cigarette smokers 25/436 (5.7%).

Again higher *Mycobacterium tuberculosis* result observed in tuberculosis symptoms like in those who have night sweating 23/436(5.2%), fever 22/436(5.0%), weight loss 20/436(4.5%), cough 24/436(5.5%), loss of appetite 20/436(4.5%), and chest pain 16/436(3.7%). The least results

were observed those who have diarrhea 3/436(0.7%), dyspnea 9/436 (2.0%), and external-adenopathy 3/436 (0.7%) sign and symptoms of *Mycobacterium tuberculosis.*

The distribution of pulmonary tuberculosis was also measured in terms of contact history with chronic coughers, smoking habit and alcoholism to trace the epidemiological features of the disease. In this study, the magnitude of pulmonary tuberculosis was not significantly high in those who had contact with TB infected patients, previous history of anti-TB treatment, drinking of alcohol and in those who were smokers. These findings were different from the studies done in Addis Ababa, Ethiopia in 2011 and north Gondar in 2015 [13, 14].

The possible reason might be due to lower number of participant diagnosis the reason for presumptive DRTB in our cases and using more number of participants from urban resident.

Higher result again observed in previously treated patients 20/436 (4.5%) with anti-TB drugs and in new patients for presumptive drug resistance tuberculosis 24/436 (5.5%).

Statistically significant association was observed between culture positive pulmonary tuberculosis and TB contact History and some of tuberculosis patient symptoms weight loss, having pneumonia and CD 4^+ counts. The previous study also indicated that pulmonary tuberculosis associated with the level of $CD4^+$ in HIV patients and the amount of virus present in the participant's blood [13, 15].

The current result seems similar with reports of 10% (20 individuals smear positive) study conducted in Addis Ababa, Ethiopia in 2017 [16], prisons settings of East Gojjam Zone, Northwest Ethiopia using Gene *X-pert* MTB/RIF, 9(3.4%) [17] and 9.9 % of the study conducted in extra pulmonary tuberculosis at University of Gondar, Northwest Ethiopia [18]. This overall culture confirmed *Mycobacterium Tuberculosis*, 27/436(6.2%) magnitude is lower than the study conducted in the Health Centers of Addis Ababa, Ethiopia reported as 46.0% (233/506) [13],

from Metehara sugar factory hospital, eastern Ethiopia (14.2%)[18] and 124 (32.2 %) of studied in two public hospitals in East Gojjam zone, northwest Ethiopia [19].

As compared to retrospective study report, from the University of Gondar Hospital from January 2013 to August 2015, prevalence of (24.6%), we found low result [20].

Our finding also lower than 23.2% of the study conducted in Debre markos Referral Hospital, Ethiopia using Gene *X-pert* MTB/RIF assay.

The possible reason for the difference might be associated with the variation of the diagnostic methods we used, for example in our cases we used sputum sedimentation concentration technique for microscopic smear examination, Gene *X-pert* assay and finally LJ culture for confirmation whereas, a single diagnostic tool used in the previous study like; stained by Ziehl-Neelsen staining and examined by Microscopy in the case of Metehara [18], using Gene *X-pert* MTB/RIF in the case of prisons settings of East Gojjam Zone [17]. This low prevalence may also mean that there might be a comparatively good TB infection control around our study area, Addis Ababa, Ethiopia.

From the overall confirmed *Mycobacterium Tuberculosis* 6.2% (27/436), a total of three *Mycobacterium tuberculosis* strain showed resistance pattern to anti-tuberculosis drug, of which two of them were multi drug (INH and RIF) resistance strains. This result lower than the study conducted in the University of Gondar Hospital, northwest Ethiopia which is resulted as 71(15%) resistant to rifampicin [20] among tuberculosis-presumptive cases at University of Gondar Hospital, northwest Ethiopia, 15.58 % of two public hospitals in East Gojjam zone, northwest Ethiopia,[19] and 12 (10.3%) patients referred to Debre markos Referral Hospital, Ethiopia [21].

From a total of 130 HIV positive status, MTB was detected in only 10 (7.7%) of the participants. Out of this sero-positive figure, one mono (INH) resistant and one MDR- TB (INH+RIF)

resistant strains were detected. Regarding participants' viral load and TB relation, only one mono resistant strain was found in the participant serum which contains high copies of viral load count ($\geq$1000/mmm^3). This might be due to HIV infection; HIV infection may cause mal-absorption of anti-TB drugs and immune suppression which leads to resistance and our result is supported by other findings [22, 23].

The bivariate logistic analysis showed that presumptive drug resistance tuberculosis two times more likely (2.6 (95% CI 0.6, 12, p=0.2)) to develop tuberculosis than presumptive tuberculosis; also having the symptoms of night sweating two times more likely (2.4(95% CI 0.8, 7.2, p=0.1)) to develop tuberculosis than those who did not the symptoms of night sweating. Having the presence of chest pain also (1.6 (95% CI 0.8, 3.7, p=0.2)) times more likely to develop *Mycobacterium tuberculosis* than from those who did not have chest pain.

[illegible]onclusion

Presence of contact history with previous tuberculosis infected patients, [illegible]ent weight loss, presence of pneumonia with radiological examination, and $CD4^+$ results were the identified symptoms and factors associated from *M. Tuberculosis* in the bivariate logistic analysis.

[illegible]eneral, this study highlights low magnitude *Mycobacterium tuberculosis* among presumptive patients visited to SPHMMC, Addis Ababa, Ethiopia, however from the total of three strains, two of MDR strains were observed on those who have history of failure, relapse and previously treated with anti-TB treatment.

Health education about tuberculosis, TB control programs should be continued and large community based study also recommended to sustain this low result of the disease.

Strengthening TB infection control activities and proper implementation of DOTS are also recommended to reduce the burden of MDR-TB.

Abbreviation

ATCC=American Type Culture Collection, DST= Drug Susceptibility Test, FMOH=Federal Ministry of Health, HIV=Human Immunodeficiency Virus, INH=Isoniazid, IQC=Internal Quality Controls, LPA=line probe assay, MDR-TB=Multidrug-Resistant Tuberculosis, MOTT= Mycobacteria other than TB, MTB= Mycobacterium Tuberculosis, OADC = Oleic Acid Albumin Dextrose Complex, PPE=Personal protective equipment, PTB= Pulmonary Tuberculosis, RIF=Rifampicin, RMR= Rifampicin Mono-Resistant, SOP= Standard Operating Procedures, SPHMMC= Saint Paul's Hospital Millennium Medical College, SR=sample reagent, STM=Streptomycin, TB=Tuberculosis, TTD= Time to detection, WHO= World Health Organization, XDR-TB= Extensively Drug-Resistant Tuberculosis.

Declaration

Ethics approval and consent to participate

The proposed study was approved by the Department of Medical Laboratory Science, Addis Ababa university research and ethics committee concerning the ethical issues giving a reference number SR/LS/025/19.

Consent for publication

Not applicable. This study does not contain any individual or personal data.

Availability of data and materials

All data relevant to this study are available on the manuscript.

Funding

This research work was supported by Addis Ababa University, Ethiopia. The funder had no role in study design, data collection and analysis, decision to publish, or preparation of the manuscript.

Authors' contributions

MK, KD, RA, ZY, AY, NZ, MA, BZ, MG and AG were involved in study conception, data collection and analysis, drafting the manuscript. MK, KD and AG reviewed critically the manuscript for intellectual content. All authors have read, edited and approved the manuscript.

Acknowledgement

Authors would like to thank the Departments of Medical Laboratory Science, College of Health Sciences, Addis Ababa University, Department of national *tuberculosis* reference laboratory staffs, members of Ethiopian Public Health Institute who were willing to use laboratory space and consumable fund, and also St. Paul's Hospital Millennium Medical College (SPHMMC) management, last but not lease we are grateful for all study participants.

Competing interests

The authors declare that they have no competing interests.

References

1. Gelaw SM. Socioeconomic Factors Associated with Knowledge on Tuberculosis among Adults in Ethiopia [Internet]. Tuberculosis Research and Treatment. 2016 [cited 2019]. Available from: https://www.hindawi.com/journals/trt/2016/6207457/

2. Churchyard GJ, Swindells S. Controlling latent TB tuberculosis infection in high-burden countries: A neglected strategy to end TB. PLOS Med. 2019 ;16(4):e1002787.

3. Organization WH. Global tuberculosis report 2015. 2015. Geneva World Health Organ. 2015;

4. Jaleta KN, Gizachew M, Gelaw B, Tesfa H, Getaneh A, Biadgo B. Rifampicin-resistant Mycobacterium tuberculosis among tuberculosis-presumptive cases at University of Gondar Hospital, northwest Ethiopia. Infect Drug Resist. 2017 ;10:185–92.

5. Asgedom SW, Tesfaye D, Nirayo YL, Atey TM. Time to death and risk factors among tuberculosis patients in Northern Ethiopia. BMC Res Notes. 2018;11(1):696.

6. Atalell KA, Tebeje NB, Ekubagewargies DT. Survival and predictors of mortality among children co-infected with tuberculosis and human immunodeficiency virus at University of Gondar Comprehensive Specialized Hospital, Northwest Ethiopia. A retrospective follow-up study. PLOS ONE. 2018 ;13(5):e0197145.

7. Woya AA, Tekile AK, Basha GW. Spatial Frailty Survival Model for Multidrug-Resistant Tuberculosis Mortality in Amhara Region, Ethiopia [Internet]. Tuberculosis Research and Treatment. 2019 [cited 2019];1(1)

8. Deribew A, Deribe K, Dejene T, Tessema GA, Melaku YA, Lakew Y, et al. Tuberculosis Burden in Ethiopia from 1990 to 2016: Evidence from the Global Burden of Diseases 2016 Study. Ethiop J Health Sci [Internet]. 2018 [cited 2019];28(5).

9. Piatek AS, Cleeff MV, Alexander H, Coggin WL, Rehr M, Kampen SV, et al. GeneXpert for TB diagnosis: planned and purposeful implementation. Glob Health Sci Pract. 2013;1(1):18–23.

10. Steingart KR, Sohn H, Schiller I, Kloda LA, Boehme CC, Pai M, et al. Xpert® MTB/RIF assay for pulmonary tuberculosis and rifampicin resistance in adults. Cochrane Database Syst Rev 2013;1(1).

11. Atashi S, Izadi B, Jalilian S, Madani SH, Farahani A, Mohajeri P. Evaluation of GeneXpert MTB/RIF for determination of rifampicin resistance among new tuberculosis cases in west and northwest Iran. New Microbes New Infect. 2017 ;19:117–20.

12. Zenebe Y, Anagaw B, Tesfay W, Debebe T, Gelaw B. Smear positive extra pulmonary tuberculosis disease at University of Gondar Hospital, Northwest Ethiopia. BMC Res Notes. 2013 ;6(1):21.

13. Deribew A, Negussu N, Melaku Z, Deribe K. Investigation Outcomes of Tuberculosis Suspects in the Health Centers of Addis Ababa, Ethiopia. PLOS ONE. 2011 ;6(4):e18614.

14. Mekonnen F, Tessema B, Moges F, Gelaw A, Eshetie S, Kumera G. Multidrug resistant tuberculosis: prevalence and risk factors in districts of metema and west armachiho, Northwest Ethiopia. BMC Infect Dis. 2015 ;15(1):461.

15. Lawn SD, Badri M, Wood R. Tuberculosis among HIV-infected patients receiving HAART: long term incidence and risk factors in a South African cohort. AIDS. 2005 ;19(18):2109.

16. Nugussie DA, Mohammed GA, Tefera AT. Prevalence of Smear-Positive Tuberculosis among Patients Who Visited Saint Paul's Specialized Hospital in Addis Ababa, Ethiopia [Internet]. BioMed Research International. 2017 [cited 2019 Oct 7].

17. Gizachew Beza M, Hunegnaw E, Tiruneh M. Prevalence and associated factors of tuberculosis in prisons settings of East Gojjam Zone, Northwest Ethiopia. Int J Bacteriol. 2017;2017.

18. 63. Yohanes A, Abera S, Ali S. Smear positive pulmonary tuberculosis among suspected patients attending metehara sugar factory hospital; eastern Ethiopia. Afr Health Sci. 2012 ;12(3):325-330–330.

19. Adane K, Ameni G, Bekele S, Abebe M, Aseffa A. Prevalence and drug resistance profile of Mycobacterium tuberculosis isolated from pulmonary tuberculosis patients attending two public hospitals in East Gojjam zone, northwest Ethiopia. BMC Public Health. 2015 ;15(1):572.

20. Jaleta KN, Gizachew M, Gelaw B, Tesfa H, Getaneh A, Biadgo B. Rifampicin-resistant Mycobacterium tuberculosis among tuberculosis-presumptive cases at University of Gondar Hospital, northwest Ethiopia. Infect Drug Resist. 2017 ;10:185–92.

21. Mulu W, Abera B, Yimer M, Hailu T, Ayele H, Abate D. Rifampicin-resistance pattern of Mycobacterium tuberculosis and associated factors among presumptive tuberculosis patients referred to Debre Markos Referral Hospital, Ethiopia: a cross-sectional study. BMC Res Notes. 2017 ;10(1):8.

22. Wells CD, Cegielski JP, Nelson LJ, Laserson KF, Holtz TH, Finlay A, et al. HIV Infection and Multidrug-Resistant Tuberculosis—The Perfect Storm. J Infect Dis. 2007 ;196(Supplement_1):S86–107.

23. Getahun H, Gunneberg C, Granich R, Nunn P. HIV Infection—Associated Tuberculosis: The Epidemiology and the Response. Clin Infect Dis. 2010 ;50(Supplement_3):S201–7.

List of tables

Table 1: Socio-demographic characteristics and magnitude of M. *tuberculosis* drug resistance pattern and its associated factors among patients referred to SPHMMC, Addis Ababa, Ethiopia, 2019.

**Variables/ characteristics**		**M. Tb Present**	**M. Tb Absent**	**No. of Participants**	**Percentages (%)**
**Sex**	Male	16	208	224	51.3
	Female	11	201	212	48.6
**Age groups**	<15 yrs	3	36	39	8.9
	15-24	4	52	56	12.8
	25-34	6	92	98	22.5
	35-49	9	118	127	29.1
	>50 yrs	5	111	116	26.6
**Residence**	Urban	13	227	240	55.0
	Rural	14	182	196	45.0
**Family size/house**	1-3	8	144	152	34.9
	4-6	16	204	220	50.5
	>6	3	61	64	14.6
**Marital status**	Single	10	136	146	33.5
	married	12	226	238	54.6

	Divorced	3	17	20	4.6
	Widowed	2	30	32	7.3
	Laborer	8	89	97	22.2
	Gov't Workers	6	91	97	22.2
**Occupational status**	Private workers	5	58	109	25.0
	House wife	5	104	70	16
	Student	3	67	63	14.4
	Illiterate	7	112	119	27.3
	1-8th grades	7	140	147	33.7
**Educational status**	9-12th grades	8	98	106	24.3
	>12th grade	5	59	64	14.7
	<100 Birr	4	56	60	13.8
	100-1000 Birr	4	79	83	19.0
	1001-2000 Birr	8	147	155	35.6
**Monthly Income**	2001-3000 Birr	5	54	59	13.5
	3001-4000 Birr	0	32	32	7.3
	4001-5000 Birr	5	20	25	5.7
	>5001 Birr	1	20	22	5.0

Table 2: Clinical characteristics for magnitude of M. *tuberculosis, drug* resistance pattern and its associated factors among patients referred to SPHMMC, Addis Ababa, Ethiopia, 2019.

**Variables/ Characteristics**		**M. Tb present**	**M. Tb Absent**	**Number of participants**	**Percentages (%)**
	Presumptive TB	25	397	422	96.8

Reason for Diagnosis	Presumptive DRTB	2	12	14	3.2
BCG Vaccination	Vaccinated	9	147	156	35.8
	Non-Vaccinated	18	262	280	64.2
TB contact History	Yes	5	28	33	7.5
	No	22	381	403	92.5
Alcohol Drinking	Yes	6	62	68	15.6
	No	21	347	368	84.4
Cigarette Smoking	Smokers	2	20	22	5.1
	Non-smokers	25	389	414	95.0
Night Sweating	Yes	23	287	310	71.1
	No	4	122	126	28.9
Presence of Fever	Yes	22	296	318	73.0
	No	5	113	118	27.0
Weight loss	Yes	20	180	200	46.0
	No	7	229	236	54.0
Presence of Cough	Yes	24	340	364	83.5
	No	3	69	72	16.5
Loss of Appetite	Yes	20	265	285	65.4
	No	7	144	151	34.6
Presence of Chest Pain	Yes	16	190	206	47.2
	No	11	219	230	52.8
Presence of Diarrhea	Yes	3	54	57	13.0
	No	24	355	379	87.0

	Yes	9	131	140	32.1
Presence of dyspnea	No	18	278	296	67.9
	Yes	3	60	63	14.4
External-Adenopathy	No	24	349	373	85.6
	Previously treated	7	103	110	25.2
Anti-TB Treatment	Previously untreated	20	306	326	74.8
	New	24	362	384	88.1
	Relapse	2	44	46	10.6
Presumptive DRTB	Failure	1	3	6	1.4
	Positive	10	120	130	29.8
HIV Status	Negative	17	289	306	70.2
	PTB	24	349	373	85.6
Tuberculosis type	EPTB	3	60	63	14.4
	<200 cells/mm^3	0	16	16	15.5
CD4$^+$ Count	200-350/mm^3	5	29	34	33.0
	>350/mm^3	1	52	53	51.5
	<1000/ mm^3	2	27	29	24.4
HIV Viral load	≥1000/ mm^3	6	84	90	75.6

Table 3**:** Bivariate analysis for socio-demographic factors among patients referred to SPHMMC, Addis Ababa, Ethiopia, 2019.

Variables/ characteristics	MTB	MTB	Total	COR (95%	P-

		Negative	Positive		CI)	value
**Sex**	Male	208	16	224	1.4(0.6-3.1)	0.4
	Female	201	11	212	1	
**Age groups**	<15 yrs	36	3	39	1.8(0.4, 8.1)	0.41
	15-24	52	4	56	1.7(0.4, 6.6)	0.44
	25-34	92	6	98	1.5(0.4, 4.9)	0.55
	35-49	118	9	127	1.7(0.6, 5.2)	0.36
	>50 yrs	111	5	116	1	
**Residence**	Urban	227	13	240	1	
	Rural	182	14	196	1.4(0.6, 2.9)	0.4
**Family size/house**	1-3	144	8	152	1	
	4-6	204	16	220	1.4(0.6, 3.4)	0.4
	>6	61	3	64	0.9(0.3, 3.5)	0.8
**Marital status**	Single	136	10	146	1	
	Married	226	12	238	1(0.2, 5.3)	0.9
	Divorced	17	3	20	0.8(0.2, 3.7)	0.7
	Widowed	30	2	32	2.6(0.4, 17)	0.3
**Occupational status**	Laborer	89	8	97	1.4(0.4, 4.6)	0.6
	Gov’t Workers	91	6	97	1.8(0.6, 5.9)	0.3
	Private workers	58	5	63	1.7(0.4, 6.4)	0.4
	Student	67	3	70	0.9(0.2, 4.0)	0.9
	House wife	104	5	109	1	
	Illiterate	112	7	119	0.7(0.3, 2.4)	0.6

**Educational status**	1-8th grades	140	7	147	0.6(0.2, 1.9)	0.4
	9-12th grades	98	8	106	0.9(0.3, 3.0)	0.9
	>12th grade	59	5	64	1	
**Monthly Income**	<100 Birr	56	4	60	1.4(0.2, 13)	0.8
	100-1000 Birr	79	4	83	1(0.1, 9)	0.9
	1001-2000 Birr	147	8	155	1(0.3, 9)	0.9
	2001-3000 Birr	54	5	59	1.8(0.2, 16)	0.6
	3001-4000 Birr	32	0	32	-	0.9
	4001-5000 Birr	20	5	25	5(0.5, 46)	0.2
	>5001 Birr	20	2	22	1	

Table 4: Bivariate analysis of clinical factors for *M. tuberculosis* among presumptive patients referred to SPHMMC, Addis Ababa, Ethiopia, 2019.

**Variables/ characteristics**		**Result of M.TB**			**COR (95%CI)**	**P-value**
		**Negative**	**Positive**	**Total**		
Reason for Diagnosis	Presumptive TB	397	25	422	1	
	Presumptive DRTB	12	2	14	2.6(0.6, 12)	0.2
BCG Vaccination	Vaccinated	147	9	156	1	
	Non-Vaccinated	262	18	280	1.1(0.5, 2.6)	0.7
TB contact History	Yes	28	5	33	3.1(1.1, 8.7)	0.03
	No	381	22	403	1	

Alcohol Drinking	Yes	62	6	68	1.6(0.6, 4.1)	0.3
	No	347	21	368	1	
Cigarette Smoking	Smokers	20	2	22	1.6(0.3,7.0)	0.5
	Non-smokers	389	25	414	1	
	[illegible]eumonia	25	3	28	3(33, 319)	0.02
	Interstitial	28	3	31	3(0.3, 30)	1.0
Chest X-ray	Bronchiectasis	11	1	12	2.6(0.3, 27)	0.34
	Bilateral	6	8	14	9(0.9, 8)	0.4
	Unilateral	14	5	19	0.5(0.6, 4.3)	0.5
	Normal	334	7	331	1	
	Untreated	103	7	110	1	
Anti-TB Treatment	Previously treated	306	20	326	1.1(0.4, 2.5)	0.9
	New	362	24	386	1	
Presumptive DRTB	Relapse	44	2	46	0.7(0.2, 3)	0.6
	Failure	3	1	4	5.0(0.5, 5.0)	0.2
	Positive	120	10	130	1.4(0.6, 3.1)	0.4
HIV Status	Negative	289	17	306	1	
CD 4 Count/ mm^3	<200	16	0	16	1.2(0.9, 2.4)	0.9
blood	200-350	29	5	34	8.9(0.5, 0.9)	0.049
	≥350	52	2	54	1	
Viral Load /mm^3	<1000	27	2	29	1	
blood	≥1000	84	6	90	0.9(0.2, 5.0)	0.9
