# Supplementary material for: Magnitude of Mycobacterium tuberculosis, drug resistance and associated factors among presumptive tuberculosis patients at St. Paul’s Hospital Millennium Medical College, Addis Ababa, Ethiopia
Source: PLoS One. 2022 Aug 1;17(8):e0272459. doi: 10.1371/journal.pone.0272459 (PMC9342779; doi:10.1371/journal.pone.0272459)
Supplement: Attachment — Submitted filename: PONE-D-22-03876_R1.pdf [file pone.0272459.s005.pdf]

PLOS ONE

Magnitude of Mycobacterium tuberculosis, drug resistance and associated factors among presumptive patients at St. Paul’s Hospital Millennium Medical College, Addis Ababa, Ethiopia

--Manuscript Draft--

Manuscript Number:	PONE-D-22-03876R1
Article Type:	Research Article
Full Title:	Magnitude of Mycobacterium tuberculosis, drug resistance and associated factors among presumptive patients at St. Paul’s Hospital Millennium Medical College, Addis Ababa, Ethiopia
Short Title:	Drug Resistance Mycobacterium tuberculosis
Corresponding Author:	Addisu Gize Yeshanew, M.Sc<br>St. Paul's Hospital Millennium Medical College<br>Addis Ababa, Ethiopia, ETHIOPIA
Keywords:	M. tuberculosis, GeneXpert MTB/RIF, Lowenstein-Jensen (LJ), Associated Factors, Addis Ababa
Abstract:	Background: Mycobacterium tuberculosis (M. tuberculosis) remains one of the most significant causes of death and a major public health problem in the community.<br>As a result, the aim of this study was to determine magnitude of Mycobacterium tuberculosis, its drug resistance, and associated factors among presumptive M. Tuberculosis (MTB) patients at St. Paul’s Hospital Millennium Medical College, Addis Ababa, Ethiopia.<br>Methods: Cross-sectional study was used at St. Paul’s Hospital Millennium Medical College (SPHMMC), Addis Ababa, Ethiopia from January to July 2019. Demographic and clinical data were collected by structured questionnaire through face to face interview.<br>Using microscopic examination and GeneXpert MTB/RIF assay and culturing in the Lowenstein-Jensen (LJ) culture media, we collected and analyzed both pulmonary and extra-pulmonary clinical samples.<br>Data were analyzed by SPSS version 23. Binary logistic regression was done to identify the associated risk factors and p-value less than 0.05 was taken as significant association.<br>Results: Of the total 436 respondents, 223(51%) were male. The mean ±SD age of the participants was 38±17years. Overall, 27/436(6.2%) of the participants had confirmed Mycobacterium tuberculosis using the GeneXpert MTB/RIF assay and LJ culture media, and two isolates were resistant to RIF and one to INH medication, with two (0.5%) being MDR-TB.<br>MTB infection was associated with previous TB contact history, patient weight loss, and CD4+ T-cell counts of 200-350/mm3 of blood.<br>Conclusion: The magnitude of M. tuberculosis and MDR-TB in this study underscores the need for improved early case detection and management of MDR-TB in order to reduce transmission and patient suffering.
Order of Authors:	Melkayehu Kassa
	Kassu Desta
	Rozina Ambachew
	Zenebe Youhannes
	Alganesh Yohannes
	Nuhamen Zena
	Misikir Amare
	Betselot Zerihun
	Melak Getu

Powered by Editorial Manager® and ProduXion Manager® from Aries Systems Corporation

	Addisu Gize, M.Sc
**Response to Reviewers:**	View Letter<br>Date:Mar 16 2022 10:05AM<br>To:"Addisu Gize Yeshanew" konjoaddisu@gmail.com<br>From:"PLOS ONE" plosone@plos.org<br>Subject:PLOS ONE Decision: Revision required [PONE-D-22-03876]<br>Attachment(s): PONE-D-22-03876_ suggesions and comnts.pdf<br><br>PONE-D-22-03876<br>Magnitude of Multidrug Resistance Mycobacterium tuberculosis and associated factors among presumptive patients at St. Paul's Hospital Millennium Medical College, Addis Ababa, Ethiopia<br>PLOS ONE<br><br>Dear Dr. Yeshanew,<br><br>Thank you for submitting your manuscript to PLOS ONE. After careful consideration, we feel that it has merit but does not fully meet PLOS ONE's publication criteria as it currently stands. Therefore, we invite you to submit a revised version of the manuscript that addresses the points raised during the review process.<br><br>Please submit your revised manuscript. If you will need significantly more time to complete your revisions, please reply to this message or contact the journal office at plosone@plos.org. When you're ready to submit your revision, log on to https://www.editorialmanager.com/pone/ and select the 'Submissions Needing Revision' folder to locate your manuscript file.<br><br>Please include the following items when submitting your revised manuscript:<br>•A rebuttal letter that responds to each point raised by the academic editor and reviewer(s). You should upload this letter as a separate file labeled 'Response to Reviewers'.<br>•A marked-up copy of your manuscript that highlights changes made to the original version. You should upload this as a separate file labeled 'Revised Manuscript with Track Changes'.<br>•An unmarked version of your revised paper without tracked changes. You should upload this as a separate file labeled 'Manuscript'.<br>If you would like to make changes to your financial disclosure, please include your updated statement in your cover letter. Guidelines for resubmitting your figure files are available below the reviewer comments at the end of this letter.<br><br>If applicable, we recommend that you deposit your laboratory protocols in protocols.io to enhance the reproducibility of your results. Protocols.io assigns your protocol its own identifier (DOI) so that it can be cited independently in the future. For instructions see: https://journals.plos.org/plosone/s/submission-guidelines#loc-laboratory-protocols. Additionally, PLOS ONE offers an option for publishing peer-reviewed Lab Protocol articles, which describe protocols hosted on protocols.io. Read more information on sharing protocols at https://plos.org/protocols?utm_medium=editorial-email&utm_source=authorletters&utm_campaign=protocols.<br><br>We look forward to receiving your revised manuscript.<br><br>Kind regards,<br><br>Frederick Quinn<br>Academic Editor<br>PLOS ONE<br>Response: Dear Frederick Quinn, Academic Editor, PLOS ONE<br>We authors would like to thank for your invitation to submit our revised version of the manuscript, PONE-D-22-03876 titled" Magnitude of Multidrug Resistance Mycobacterium tuberculosis and associated factors among presumptive patients at St. Paul's Hospital Millennium Medical College, Addis Ababa, Ethiopia", that addresses the points raised during the review process.<br>We included a point-by-point response within the 'Response to Reviewers' box in the submission system and modifications are highlighted in the track changes of the

original manuscript, and we uploaded also the clean or unmarked version of our revised paper without tracked changes.
Journal Requirements:
When submitting your revision, we need you to address these additional requirements.
1. Please ensure that your manuscript meets PLOS ONE's style requirements, including those for file naming. The PLOS ONE style templates can be found at https://journals.plos.org/plosone/s/file?id=wjVg/PLOSOne_formatting_sample_main_body.pdf and
https://journals.plos.org/plosone/s/file?id=ba62/PLOSOne_formatting_sample_title_authors_affiliations.pdf
Response: Thank you for the concern, the PLOS ONE's style requirement has been incorporated accordingly in all sections.
2. Thank you for submitting the above manuscript to PLOS ONE. During our internal evaluation of the manuscript, we found significant text overlap between your submission and the following previously published works, some of which you are an author.
- https://www.hindawi.com/journals/trt/2016/6207457/
- https://www.dovepress.com/rifampicin-resistant-mycobacterium-tuberculosis-among-tuberculosis-pre-peer-reviewed-fulltext-article-IDR
- https://www.ghspjournal.org/content/1/1/18.full
- https://www.ncbi.nlm.nih.gov/pmc/articles/PMC3557681/
We would like to make you aware that copying extracts from previous publications, especially outside the methods section, word-for-word is unacceptable. In addition, the reproduction of text from published reports has implications for the copyright that may apply to the publications.
Please revise the manuscript to rephrase the duplicated text, cite your sources, and provide details as to how the current manuscript advances on previous work. Please note that further consideration is dependent on the submission of a manuscript that addresses these concerns about the overlap in text with published work.
We will carefully review your manuscript upon resubmission, so please ensure that your revision is thorough.
Response: Thank you for your concern again and the constructive comment. We have rephrased and completely modified each word taken from the previous published articles.
3. Please include additional information regarding the survey or questionnaire used in the study and ensure that you have provided sufficient details that others could replicate the analyses. For instance, if you developed a questionnaire as part of this study and it is not under a copyright more restrictive than CC-BY, please include a copy, in both the original language and English, as Supporting Information.
Response: Regarding our survey tool or questionnaire, we have uploaded as supporting file and any interested can replicate for the study and analyses.
4. Thank you for stating the following in the Acknowledgments Section of your manuscript:
"Authors would like to thank the Departments of Medical Laboratory Science, College of Health
Sciences, Addis Ababa University, Department of national tuberculosis reference laboratory
staffs, members of Ethiopian Public Health Institute who were willing to use laboratory space
and consumable fund, and also St. Paul's Hospital Millennium Medical College (SPHMMC)
management, last but not lease we are grateful for all study participants."
We note that you have provided funding information that is not currently declared in your Funding Statement. However, funding information should not appear in the Acknowledgments section or other areas of your manuscript. We will only publish funding information present in the Funding Statement section of the online submission form.
Please remove any funding-related text from the manuscript and let us know how you would like to update your Funding Statement. Currently, your Funding Statement reads as follows:
"This research work was supported by Addis Ababa University, Ethiopia.
The funders had no role in study design, data collection and analysis, decision to publish, or preparation of the manuscript."
Please include your amended statements within your cover letter; we will change the

online submission form on your behalf.
Response: We included our Funding statement in the cover letter, same time the statement which stated about our funding information is removed from the revised manuscript.
5. Your ethics statement should only appear in the Methods section of your manuscript. If your ethics statement is written in any section besides the Methods, please move it to the Methods section and delete it from any other section. Please ensure that your ethics statement is included in your manuscript, as the ethics statement entered into the online submission form will not be published alongside your manuscript.
[Note: HTML markup is below. Please do not edit.]
Response: The comment is well taken and based on the comment; we have stated the ethics statement only in the method section of the revised manuscript.

Reviewers' comments:

Reviewer's Responses to Questions
Comments to the Author

1. Is the manuscript technically sound, and do the data support the conclusions?

The manuscript must describe a technically sound piece of scientific research with data that supports the conclusions. Experiments must have been conducted rigorously, with appropriate controls, replication, and sample sizes. The conclusions must be drawn appropriately based on the data presented.
Reviewer #1: Partly
Reviewer #2: Partly
Response: It is true that one’s perception different from other. However, we author’s believe that our manuscript technically sound, and do the data support the manuscript conclusions.

____
2. Has the statistical analysis been performed appropriately and rigorously?
Reviewer #1: No
Reviewer #2: I Don't Know
Response: Again it may be due to personal difference or may be because of reviewers are not public health professionals. However, we author’s consulted by health professionals for our statistical analysis, and we believe that the statistical analysis has been performed appropriately.

____
3. Have the authors made all data underlying the findings in their manuscript fully available?

The PLOS Data policy requires authors to make all data underlying the findings described in their manuscript fully available without restriction, with rare exception (please refer to the Data Availability Statement in the manuscript PDF file). The data should be provided as part of the manuscript or its supporting information, or deposited to a public repository. For example, in addition to summary statistics, the data points behind means, medians and variance measures should be available. If there are restrictions on publicly sharing data—e.g. participant privacy or use of data from a third party—those must be specified.
Reviewer #1: Yes
Reviewer #2: Yes
Response: Thank you.

____
4. Is the manuscript presented in an intelligible fashion and written in standard English?

PLOS ONE does not copyedit accepted manuscripts, so the language in submitted articles must be clear, correct, and unambiguous. Any typographical or grammatical errors should be corrected at revision, so please note any specific errors here.
Reviewer #1: No
Reviewer #2: No
Response: The comment is well taken. By now our manuscript revised detail to have intelligible fashion and Standard English language presentation to make it clear for the readers.

5. Review Comments to the Author

Please use the space provided to explain your answers to the questions above. You may also include additional comments for the author, including concerns about dual publication, research ethics, or publication ethics. (Please upload your review as an attachment if it exceeds 20,000 characters)

Reviewer #1: his deals on an important and current issue, TB and drug resistance TB in Ethiopia. However, the manuscript poorly organized and need major revision. I try to mention some points on the main manuscript but many more left.

Response: Again, this comment is well taken. Based on each comment mentioned in the main manuscript document, we have done major revision and incorporated the suggested comments on the revised manuscript.

We authors glad to appreciate reviewer#1effort for his comment/concern and questions for unclear points found in main manuscript document.

Reviewer #2: Comments

The author picked an interesting topic that has public health importance but it is not a new topic. Such kind of work has important role in monitoring the distribution of MDR-TB in the study area. The study aimed to determine the magnitude of MDR-TB and associated factors as it is seen from the title of the manuscript and objectives mentioned in the abstract; however, in the background, results, and discussion there is a different scenario. There are concerns I encountered in this manuscript some of them are: what was the aim of this study?

Response: Thank you for your interest and stating the importance of the study as public health problem. By now, we have taken your comment and concern and define the objective of the study.

Why it vary from one section to the other? There are inconsistencies in this regard that can affect the results, discussion, and conclusion section.

Response: Again the comment is well taken. We have modified many things in the revised manuscript to keep the consistency in result, discussion and conclusion.

The other one is the sample size issue, unless it is resolved, I think it will affect the manuscript.

Response: With all due respect to the reviewer, we believe that this point is not correct and our sample size is calculated with single population proportion formula, and by now it is resolved in the revised document.

Authors are advised to carefully revise the manuscript. My comments are depicted below.

Response: Well come for your comments and thank you for investing your time on our manuscript.

I. General comments

1. Revise language

Response: The comment is well taken. By now our manuscript revised detail to have intelligible fashion and Standard English language presentation to make it clear for the readers.

2. Follow the scientific way of writing to name of bacteria

Response: We agree with this reviewer that very few bacteria were named non-scientifically, but now we have scientifically corrected their names.

3. There are several typographical errors that need revision for example in citing a reference in one place it is placed in [] in some other place it is presented as superscript. Sometimes reference which is not on the list is cited.

Response: Thank you for your constructive comment. We have corrected our manuscript citation and references based on the journal requirement.

4. Proper use of abbreviations: If abbreviations appear for the first time in the manuscript use the expanded form then after use the shortened form.

Response: Again the comment is well taken, and by now we have corrected our manuscript abbreviation.

5. I suggest the use of ‘GeneXpert’ instead of “X-pert MTB/RIF”

Response: Again the comment is well taken, and by now we have corrected our manuscript as per suggestion as GeneXpert.

Powered by Editorial Manager® and ProduXion Manager® from Aries Systems Corporation

II. Abstract
1. Several typos example "….from Jan to July 2019…" change Jan to January
Response: Thank you. We have corrected as per the comment.

2. The title does not reflect the objective (prevalence of MTB vs Prevalence of MDR; factors associated with the magnitude of MTB? Or MDR? ….justify or modify the title
Response: The comment is accepted. Based on the comment the title modified as "Magnitude of Mycobacterium tuberculosis, Drug resistance and associated factors among presumptive patients at St. Paul's Hospital Millennium Medical College, Addis Ababa, Ethiopia"

3. The background in the abstract does not reflect the title
Response: Based on the comment, now our background in the abstract section corrected to reflect the title.

4. Which method was used to detect MDR? The method in the abstract needs revision it should go with the title and objective (detection of MDR) what was the need for microscopy?
Response: The methods used for MDR detection were GeneXpert MTB/RIF assay and LJ culture media. We used Microscopy for cross checking, and now we have revised the abstract section.

5. What was the sample size of this study? 422? 436?
Response: It was proposed the minimum sample size using single population formula as 422. However, we used 436 participants in the actual data collection procedure (more than the determined sample size) during the study period.

6. Omit socio-demographic data, focus on the main findings: MDR, factors associated with MDR (this was not shown anywhere in the manuscript)
With all due respect to the reviewer, we believe that this point is not correct. Socio-demographic data is mandatory for magnitude of M. tuberculosis and to describe the statistical associations.
7. A statement "Out of the total participants, the overall confirmed Mycobacterium tuberculosis was through X-pert MTB/RIF assay and LJ culture media was 27 (6.2%), and three isolates were resistant for either INH or RIF drug, while two of them were MDR-TB based on line probe assays method" needs revision
Response: Based on the comment, we have revised the stated statement.

8. In a statement "Previous TB-contact history, patient weight loss, having pneumonia with chest X-ray finding, and CD4+ T-cells count 200-350/mm3 of blood were significantly associated predictors for MTB infection" does not go with the title and objective. As I understand the aim of the study is to determine factors associated with MDR-TB
Response: Based on the above comment, now we have revised the title to reflect exactly the objective and the associated factors.

9. What is the prevalence of MDR and its predictors, these two should be given emphasis in results and conclusion.
Response: Thank you very much. The prevalence of MDR-TB was 0.5%, and the predictors are not previous TB-contact history, patient weight loss, CD4+ T-cells count 200-350/mm3 of blood were significantly associated predictors for MTB infection, and not for MDR-TB.

III. Background
1. The first sentence "…..caused by strains belonging to …" as strains is inappropriately used, modify as "…caused by Mycobacterium tuberculosis complex…"
i. In a statement "Globally, the estimated prevalence of MDR-TB was 3.3% in newly diagnosed patients in the WHO 2015 report. This was higher to 20% in patients with a history of anti-TB treatment(30)." Reference # 30 does not exist in the reference list.
Response: The comment is well taken and raised by the first reviewer, and by now it correctly cited.

2. Use updated information in the background: 2017 WHO report is old
Response: Thank you for your suggestion and our manuscript is revised using the

	updated WHO 2020 report.<br><br>3. Also use the updated information for Ethiopia instead of using 2005 report<br>Response: Thank you for your suggestion and our manuscript is revised using the updated 2018 national study.<br><br>4. The last sentence of the background is not in line with what has been mentioned in the title and abstract. They contradict each other, justify or revise<br>a. “Therefore, the goal of this study was to determine magnitude of Mycobacterium tuberculosis and its associated factors among TB- presumptive patients referred to St. Paul’s Hospital Millennium Medical College, Addis Ababa, Ethiopia.”<br>b. “Therefore, the goal of this study was to determine magnitude of Multi Drug Mycobacterium tuberculosis (MDR-TB) and its associated factors among TB- presumptive patients at St. Paul’s Hospital Millennium Medical College, Addis Ababa, Ethiopia”<br>Response: Thank you for your comment and now we have revised as suggested in the option a.<br><br>5. Use another word instead of the “the goal of this study…”<br>Response: We changed the goal of this study by the aim of this study.<br><br>IV. Materials and Methods<br>1. In the study area, mention the TB clinic (patient flow, service…etc)<br>Response: The comment is taken and described in the revised document.<br><br>2. What does ‘Jan’ stands for? Is it January?<br>Response: Yes to mean that January. Now we have corrected it as January in the revised manuscript.<br>3. Study variables should go with the title and objective of the study: MDR-TB and predictors of MDR-TB<br>Response: Now we have corrected the title and objective of the study, and both dependent and independent variables are in line with the revised title and objectives.<br><br>4. Determination of sample size is not clear. If p=23% (reference is not given, it is not clear whether it is the prevalence of TB or MDR), d=5% is used the sample size will be 272 + 10% (n=299) which is different from what is mentioned in the manuscript (n=422). Moreover, a sample size different from these two was used in the result section (n=436).<br>Response: The comment is accepted. Now we have revised that our sample size calculation using single population proportion formula, and the minimum sample size for the study was 422 participants. However that, during the actual collection of the study period we have collected data from 436 participants. (I think it is expected to collect more data from the pre-determined sample to be more representative).<br>5. Mention sampling technique; non-probability sampling technique (convenient)? Systematic random sampling technique? Random sampling technique?<br>Response: We have already mentioned our sampling technique as “consecutive sampling technique was used to select the study population”, which is usually applicable to collect data from health institution as probability sampling techniques, since it is difficult to apply Systematic random sampling technique or random sampling technique. This sampling technique is considered as probability sampling method in most literatures.<br>6. Detail is needed on data collection procedure: how the questionnaire was validated? Move clinical specimen collection to the laboratory section.<br>Response: It is already stated as “the questionnaire was pre-tested and proper training prior to the actual data collection was given for data collectors. The necessary adjustments were made after the pre-test”, in the data quality assurance section and collection procedure is well revised and categorized as: Collection using questionnaire, laboratory collection procedures, using GeneXpert, Microscopic examination and culturing method.<br>7. How the sample was collected from children who are unable to provide sputum? I think it is difficult to obtain sputum from children, how did you handle it?<br>Response: Thank you for your concern. We were describing that taking from participants of 2-4 ml of clinical sputum samples, pus, lymph node aspirate or peritoneal, pleural fluid and gastric aspirate. Sputum sample was taken usually from

Powered by Editorial Manager® and ProduXion Manager® from Aries Systems Corporation

children older than 10 years who can produce sputum. We used Gastric aspirate or induced sputum by physician support for children unable to provide sputum by coughing.

8. In Laboratory procedures section mention what was done to identify MTB, MDR (microscopy?, culture, GeneXpert) instead of description or performance of the methods.
Response: The comment is accepted, and the procedures are mentioned in the revised manuscript.

9. I am not sure if the use of trademark is allowed by the journal (®), check it out.
Response: The comment is accepted and removed from the revised document.

10. Data quality assurance: mention how the quality of data was maintained both for socio-demographic, clinical characteristics, and Laboratory data
Response: Thank you. By now we have clearly mentioned.
11. Data analysis and interpretation: what does data editing mean? You should have considered multivariate analysis.
Response: The comment is taken and removed unnecessary words, and regarding to the multivariable analysis consideration, we agree with the reviewer the importance of multivariate analysis in terms of controlling the compfounders. However that, no socio-demographic factors was a candidate (p<0.25) for multivariable analysis in the table 3, and for few variable that were associated in the table 4, unfortunately we did not do analysis for multivariable analysis.

12. In a statement "…to assess the association between different factors.." what are these factors?
Response: Again the comment is taken and corrected as " to assess the association between TB and risk factors"

13. Ethical consideration: correct "…Department of Medical Laboratory Sciences" as "…….Science" check if it is school or department. Does the department of Medical Laboratory Science have IRB; most of the time IRB is at the college level.
Response: It is corrected as Science. Yes, it is department not the school, and has its own IRB.

14. In the sentence "…then submitted to laboratory department" which laboratory department? And why?
Response: I think this is clear, Laboratory department means the actual study area which data was collected, it is St. Paul's Hospital Millennium Medical College, Microbiology laboratory department, while the study was approved by Department of Medical Laboratory Science, Addis Ababa, University.

15. Did you obtain assent for children?
Response: Yes. Now we have stated in the ethics section of the revised manuscript.

16. Include operational definition for MDR, presumptive TB, and presumptive MDR-TB
Response: Thank you for your constructive comments. By now we have included our operation definition for MDR TB, presumptive TB, and presumptive MDR-TB

V. Results
1. The whole result is based on the sample size (436vs422vs299) which is different from the one mentioned in the method section (where do 436 come from?).
Response: The minimum sample size calculated or predetermined was 422, however the actual study participants were 436, and it is advised to use large sample size in any study to be more representative of the study result.

2. "…had monthly income 100-1000 Ethiopian Birr, table 1." May be re-written as "……………Ethiopian Birr (Table 1)." Do the same wherever it applies.
Response: The comment is taken and modified based on the comment.
3. In a clinical data: "About 422 (96.8%) of the participants were presumptive TB.." how is this different from "From the total 374 (85.8%) were suspected for pulmonary tuberculosis and 62 (14.2%) were suspected for extra-pulmonary tuberculosis"
Response: No difference in the interpretation except different wording. By now it is

Powered by Editorial Manager® and ProduXion Manager® from Aries Systems Corporation

corrected be consistent as presumptive TB.
4. Why most of the study participants are HIV positive? What kind of impact does it have on your study?
Response: Of the total 130 (30%) were HIV positive participants, of which104 (81%) were on anti-HIV treatment. These may have an impact to be infected with MTB or the chance to develop TB or MDR-TB.
5. Rename subheading 'Bivariate analysis' as 'Factors associated with MDR-TB'
Response: The comment is accepted and modified as reviewer's wish bivariate analysis to factor associated to MTB.
6. As mentioned above, the analysis does not go with title and objective and also multivariate analysis was not conducted.
Response: The comment was taken from the previous concern and suggestion. By now the title is modified as "Magnitude of Mycobacterium tuberculosis, Drug resistance and associated factors among presumptive patients at St. Paul's Hospital Millennium Medical College, Addis Ababa, Ethiopia", which is in line with the objective and the analysis of the manuscript.

VI. Discussion
1. Because of some points mentioned above, the discussion is not in order: it should follow the main findings of the study: should discuss the magnitude of MDR that is followed by risk factors. Discussion can be re-written after addressing the comments given above.
Response: The comment is taken and our manuscript is revised based on the comment.
2. Some types, page 11: "Mycobacterium Tuberculosis" correct as Mycobacterium tuberculosis, 'East Gojjam zone, northwest Ethiopia,19" & "eastern Ethiopia (14.2%)18 " references are in superscript put them in []
Response: Thank you. By now it is correctly cited as [18].
3. In a statement "The possible reason for the difference might be associated with the variation of the diagnostic methods we used, for example in our cases we used sputum sedimentation concentration technique for microscopic smear examination, Gene X-pert assay and finally LJ culture for"confirmation whereas, a single diagnostic tool used in the previous study like;stained by ZiehlNeelsen staining and examined by Microscopy in the case of Metehara [18]" in the method section you did not mention that you have used sputum sedimentation concentration technique. Moreover if you have used all this the fining should be higher.
Response: Thank you very much again. Now it is clearly stated in the method section of the revised manuscript about sputum sedimentation concentration technique. In addition the overall result of the study will be lower because of minimizing of false positive using parallel different diagnostic and confirmatory methods.
VII. Conclusions
1. Conclusion needs revision
Response: The constructive comment is well taken, and we have revised the conclusion section.
VIII. References and Tables
2. Reference writing style is not uniform (#1, 7, 8, 16, 18,), Add URL for refer #3,
Response: By now, we have made. All the references are uniform which have URL and PubMed citation format with their own doi and PMID in the revised manuscript.
3. Tables: the labeling of table 1 is not correct (it is not for MDR?), you may omit Colum 3 and 4 (MTB present & MTB absent), the age category could have been reduced. Replace 'illiterate' with no formal education, monthly income category is not reasonable.
Response: We have revised the table title and its content as per the comment.
The same comments apply for table 2 (correct labeling of the table and omit Colum 3 &4.
Response: We have revised the table title and its content as per the comment.
Expand all abbreviations mentioned in the table under the tables. Re-write labeling of table 3 &4 analysis of what with what (MDR with socio or TB with socio) please check your title of manuscript and objective.
Response: We have revised the table title and its content as per the comment.
And also consider multivariate analysis for variables with p<0.25. In the tables include both frequency and percentages in each cell.
Response: Regarding to the multivariable analysis consideration, we agree with the reviewer the importance of multivariate analysis in terms of controlling the

	compfounders. However that, no socio-demographic factors was a candidate (p<0.25) for multivariable analysis from table 3, and for few variable that were associated in the table 4, unfortunately we did not do analysis for multivariable analysis.____<br>6. PLOS authors have the option to publish the peer review history of their article (what does this mean?). If published, this will include your full peer review and any attached files.<br><br>If you choose "no", your identity will remain anonymous but your review may still be made public.<br><br>Do you want your identity to be public for this peer review? For information about this choice, including consent withdrawal, please see our Privacy Policy.<br>Reviewer #1: No<br>Reviewer #2: No<br>Response: No problem.<br>[NOTE: If reviewer comments were submitted as an attachment file, they will be attached to this email and accessible via the submission site. Please log into your account, locate the manuscript record, and check for the action link "View Attachments". If this link does not appear, there are no attachment files.]<br><br>While revising your submission, please upload your figure files to the Preflight Analysis and Conversion Engine (PACE) digital diagnostic tool, https://pacev2.apexcovantage.com/. PACE helps ensure that figures meet PLOS requirements. To use PACE, you must first register as a user. Registration is free. Then, login and navigate to the UPLOAD tab, where you will find detailed instructions on how to use the tool. If you encounter any issues or have any questions when using PACE, please email PLOS at figures@plos.org. Please note that Supporting Information files do not need this step.____<br>In compliance with data protection regulations, you may request that we remove your personal registration details at any time. (Remove my information/details). Please contact the publication office if you have any questions.
**Additional Information:**	
**Question**	**Response**
**Financial Disclosure**<br><br>Enter a financial disclosure statement that describes the sources of funding for the work included in this submission. Review the submission guidelines for detailed requirements. View published research articles from *PLOS ONE* for specific examples.<br><br>This statement is required for submission and **will appear in the published article** if the submission is accepted. Please make sure it is accurate.	This research work was supported by Addis Ababa University, Ethiopia.<br>The funders had no role in study design, data collection and analysis, decision to publish, or preparation of the manuscript.

**Unfunded studies**<br>Enter: *The author(s) received no specific funding for this work.*<br><br>**Funded studies**<br>Enter a statement with the following details:<br>• Initials of the authors who received each award<br>• Grant numbers awarded to each author<br>• The full name of each funder<br>• URL of each funder website<br>• Did the sponsors or funders play any role in the study design, data collection and analysis, decision to publish, or preparation of the manuscript?<br>• **NO** - Include this sentence at the end of your statement: *The funders had no role in study design, data collection and analysis, decision to publish, or preparation of the manuscript.*<br>• **YES** - Specify the role(s) played.<br><br>* typeset	
**Competing Interests**<br><br>Use the instructions below to enter a competing interest statement for this submission. On behalf of all authors, disclose any competing interests that could be perceived to bias this work—acknowledging all financial support and any other relevant financial or non-financial competing interests.<br><br>This statement is required for submission and **will appear in the published article** if the submission is accepted. Please make sure it is accurate and that any funding sources listed in your Funding Information later in the submission form are also declared in your Financial Disclosure statement.<br><br>View published research articles from *PLOS ONE* for specific examples.	The authors have declared that no competing interests exist.

**NO authors have competing interests**<br><br>Enter: *The authors have declared that no competing interests exist.*<br><br>**Authors with competing interests**<br><br>Enter competing interest details beginning with this statement:<br><br>*I have read the journal's policy and the authors of this manuscript have the following competing interests: [insert competing interests here]*<br><br>* typeset	
**Ethics Statement**<br><br>Enter an ethics statement for this submission. This statement is required if the study involved:<br><br>• Human participants<br>• Human specimens or tissue<br>• Vertebrate animals or cephalopods<br>• Vertebrate embryos or tissues<br>• Field research<br><br>Write "N/A" if the submission does not require an ethics statement.<br><br>General guidance is provided below. Consult the submission guidelines for detailed instructions. **Make sure that all information entered here is included in the Methods section of the manuscript.**	The proposed study was approved by the Department of Medical Laboratory Science, Addis Ababa university research and ethics committee concerning the ethical issues giving a reference number SR/LS/025/19.

**Format for specific study types**<br><br>**Human Subject Research (involving human participants and/or tissue)**<br>• Give the name of the institutional review board or ethics committee that approved the study<br>• Include the approval number and/or a statement indicating approval of this research<br>• Indicate the form of consent obtained (written/oral) or the reason that consent was not obtained (e.g. the data were analyzed anonymously)<br><br>**Animal Research (involving vertebrate animals, embryos or tissues)**<br>• Provide the name of the Institutional Animal Care and Use Committee (IACUC) or other relevant ethics board that reviewed the study protocol, and indicate whether they approved this research or granted a formal waiver of ethical approval<br>• Include an approval number if one was obtained<br>• If the study involved *non-human primates*, add *additional details* about animal welfare and steps taken to ameliorate suffering<br>• If anesthesia, euthanasia, or any kind of animal sacrifice is part of the study, include briefly which substances and/or methods were applied<br><br>**Field Research**<br><br>Include the following details if this study involves the collection of plant, animal, or other materials from a natural setting:<br>• Field permit number<br>• Name of the institution or relevant body that granted permission	
**Data Availability**<br><br>Authors are required to make all data underlying the findings described fully available, without restriction, and from the time of publication. PLOS allows rare exceptions to address legal and ethical concerns. See the PLOS Data Policy and FAQ for detailed information.	Yes - all data are fully available without restriction

A Data Availability Statement describing where the data can be found is required at submission. Your answers to this question constitute the Data Availability Statement and **will be published in the article**, if accepted.<br><br>**Important:** Stating 'data available on request from the author' is not sufficient. If your data are only available upon request, select 'No' for the first question and explain your exceptional situation in the text box.<br><br>Do the authors confirm that all data underlying the findings described in their manuscript are fully available without restriction?	
**Describe where the data may be found in full sentences. If you are copying our sample text, replace any instances of XXX with the appropriate details.**<br><br>• If the data are **held or will be held in a public repository**, include URLs, accession numbers or DOIs. If this information will only be available after acceptance, indicate this by ticking the box below. For example: *All XXX files are available from the XXX database (accession number(s) XXX, XXX.).*<br>• If the data are all contained **within the manuscript and/or Supporting Information files**, enter the following: *All relevant data are within the manuscript and its Supporting Information files.*<br>• If neither of these applies but you are able to provide **details of access elsewhere**, with or without limitations, please do so. For example:<br><br>*Data cannot be shared publicly because of [XXX]. Data are available from the XXX Institutional Data Access / Ethics Committee (contact via XXX) for researchers who meet the criteria for access to confidential data.*<br><br>*The data underlying the results presented in the study are available from (include the name of the third party*	All data relevant to this study are available on the manuscript.

and contact information or URL).<br>• This text is appropriate if the data are owned by a third party and authors do not have permission to share the data.<br><br>* typeset	
Additional data availability information:	

Manuscript

Click here to access/download;Manuscript;Unmarked_Cleaned_Manuscript_1 -Jo.docx

Magnitude of *Mycobacterium tuberculosis*, drug resistance and associated factors among presumptive patients at St. Paul's Hospital Millennium Medical College, Addis Ababa, Ethiopia

Melkayehu Kassa[1], Kassu Desta[2], Rozina Ambachew[1], Zenebe Gebreyohannes[1], Alganesh Gebreyohanns[1], Nuhamen Zena[1], Misikir Amare[3], Betselot Zerihun[3], Melak Getu[3] and Addisu Gize[1*]

[1]St.Paul's Hospital Millennium Medical College, Addis Ababa, Ethiopia

[2]College of Health Science, Addis Ababa University, Addis Ababa, Ethiopia

[3]Ethiopian Public Health Institute, Addis Ababa, Ethiopia

Authors' Email:

Melkayehu Kassa= kassamelkayehu@gamil.com

Kassu Desta= kassudesta2020@gmail.com

Rozina Ambachew= nanieyasu@gmail.com

Zenebe Gebreyohannes= zenebezoha@gmail.com

Alganesh Gebreyohanns= alganesh5838@gmail.com

Nuhamen Zena= nuhamenzf@gmail.com

Misikir Amare= misikiramare.ma@gmail.com

Betselot Zerihun= betselotzerihun3@gmail.com

Melak Getu= melakgetu2005@gmail.com

Addisu Gize= addisu.gize@sphmmc.edu.et

*Corresponding author= Email: addisu.gize@sphmmc.edu.et , or konjoaddisu@gmail.com (AG)

Abstract

Background: *Mycobacterium tuberculosis* (*M. tuberculosis*) remains one of the most significant causes of death and a major public health problem in the community.

As a result, the aim of this study was to determine magnitude of *Mycobacterium tuberculosis,* its drug resistance, and associated factors among presumptive *M. Tuberculosis* (MTB) patients at St. Paul's Hospital Millennium Medical College, Addis Ababa, Ethiopia.

Methods: Cross-sectional study was used at St. Paul's Hospital Millennium Medical College (SPHMMC), Addis Ababa, Ethiopia from January to July 2019. Demographic and clinical data were collected by structured questionnaire through face to face interview.

Using microscopic examination and GeneXpert MTB/RIF assay and culturing in the Lowenstein-Jensen (LJ) culture media, we collected and analyzed both pulmonary and extra-pulmonary clinical samples.

Data were analyzed by SPSS version 23. Binary logistic regression was done to identify the associated risk factors and p-value less than 0.05 was taken as significant association.

Results: Of the total 436 respondents, 223(51%) were male. The mean ±SD age of the participants was 38±17years. Overall, 27/436(6.2%) of the participants had confirmed *Mycobacterium tuberculosis* using the GeneXpert MTB/RIF assay and LJ culture media, and two isolates were resistant to RIF and one to INH medication, with two (0.5%) being MDR-TB.

MTB infection was associated with previous TB contact history, patient weight loss, and CD4+ T-cell counts of 200-350/mm3 of blood.

Conclusion: The magnitude of *M. tuberculosis* and MDR-TB in this study underscores the need for improved early case detection and management of MDR-TB in order to reduce transmission and patient suffering.

Key words: *M. tuberculosis, GeneXpert MTB/RIF, Lowenstein-Jensen (LJ)***,** Associated Factors, Addis Ababa.

Background

Tuberculosis (TB) is a disease caused by *Mycobacterium tuberculosis* complex bacteria. The most common mode of transmission is through the respiratory system when an infected person coughs or sneezes near healthy people. However, *Mycobacterium bovis* can infect us while we are consuming and drinking unboiled milk from infected cattle [1]. World Health Organization (WHO) estimated that 9.9 million people developed tuberculosis (TB) and 1.5 million died of TB globally in 2020 and 22% of the world population are infected with latent *Mycobacterium tuberculosis*, of which 95% cases occur in resource limited settings [2]. According to a WHO report published in 2019, estimates that 3.4% of new patients, and 18% of previously treated cases have MDRTB [3].

According to the same report, the incidence of tuberculosis in Ethiopia is estimated to be 247 per 100,000, placing the country seventh in the world and fourth among Sub-Saharan African countries with significant TB burdens [3, 4]. The epidemiology of MDR-TB in Ethiopia was studied in 2018 and revealed an overall prevalence of 7.24%, with 2.1% of new cases and 21.07%t of retreatment cases [4].

A higher mortality rate was observed in different health institutions in Northern Ethiopia; 87 (11.3%) patients died in Mekelle Hospital and Ayder Comprehensive Hospital [5], 38 (14.02%) children from TB/HIV co-infected University of Gondar Comprehensive Specialized Hospital [6], and 61(29.47%) of the patients died in different hospitals of Amhara region, Northwest Ethiopia [7]. In general, the TB mortality rate in Ethiopia decreased from 393.8/100,000 to 100/100,000 between 1990 and 2016 (a 75% decrease), indicating a slow decline. As a result,

study suggested that males had a higher TB mortality rate than females [8].The common microscopic examination accuracy for *M. tuberculosis* in sputum stained smear difference ranged from 20% in the light microscope up 80% in the case of fluorescence method applications.

Despite this range difference, it could be used to detect TB when the clinical sample contains sufficient tuberculosis micro-organism without testing of its resistant pattern. This leads to missing of the causative agent detection in immunocopromised individuals like those who are living with HIV and children since they could not produce enough sputum which has low numbers of bacilli [9].

Hence, GeneXpert MTB/RIF assay test is sensitive and specific and an initial diagnostic test for TB and rifampicin resistance detection in patients suspected of having TB, MDR-TB or HIV-associated TB [10]. Therefore, the aim of this study was to determine magnitude of *Mycobacterium tuberculosis,* its drug resistance and associated factors among presumptive TB patients referred to St. Paul's Hospital Millennium Medical College, Addis Ababa, Ethiopia.

Materials and methods

Study area

The study was conducted in St Paul's Hospital Millennium Medical College, Addis Ababa, Ethiopia. The hospital receives referrals from throughout the country. It has currently 392 beds, with an annual average of 200,000 patients and a catchment population of more than 5 million. On average, the microbiology laboratory receives and process five sputum samples for pulmonary tuberculosis and TB and two extra-pulmonary tuberculosis clinical samples per day.

Study design and period

From January to July 2019, a cross-sectional study was conducted at SPHMMC in Addis Ababa, Ethiopia. All patients who visited the hospital were the source population, and all patients with the diagnosis of presumptive pulmonary tuberculosis who visited the microbiology laboratory and met the inclusion criteria were the study population.

Inclusion and Exclusion criteria

All patients with a presumptive diagnosis of *Mycobacterium tuberculosis* who visited a microbiology lab were included in the study. Patients with insufficient specimens or a history of known *Mycobacterium tuberculosis* treatment resistance were excluded from the study.

Variables

Magnitude of *Mycobacterium tuberculosis* and its drug resistance pattern among presumptive patients were dependent variables. Whereas, socio-demographic characteristics (age, sex, etc.), possible risk factors like; TB contact history, previous treatment for TB, presumptive Drug Resistance Tuberculosis (DRTB), BCG vaccination status, $CD4^{+}$ and HIV viral load counts were independent variables.

Sample size and sampling technique

To get the maximum sample size, the sample size was determined using the assumption of a single population proportion formula, taking into account a proportion of 50%, a margin of error of 5%, and a confidence level of 95%. The calculation result was as follows:

$$n = \frac{(z^{\alpha}/_{2})^2\, p\,(1-p)}{d^2} \Longrightarrow \frac{(1.96)^2\, 0.5(1-0.5)}{(0.05)^2} = 384 \text{ study subjects.}$$

Where: n = minimum sample size,

P = estimated proportion of patients with *Mycobacterium tuberculosis* for the study population, and taking 10% non-response rate, the final sample size become 422 participants.

d= the margin of sample error, $z^{\alpha}/_{2}$= the standard normal variable at 1-$^{\alpha}/_{2}$ confidence level and we used consecutive sampling technique was used to select the study population.

Data collection procedure

Data collectors were given training and instructions on how to collect the information. The study participants' socio-demographic status and related risk variables were collected using a structured questionnaire. For each patient with a presumptive diagnosis of *Mycobacterium tuberculosis*, a 2-4 mL of clinical sputum pus, lymph node aspirate or peritoneal and pleural fluid, and gastric aspirate samples were collected. For children who were unable to cough up sputum, we used gastric aspirate or induced sputum with physician assistance.

Laboratory procedures

For all samples, GeneXpert MTB/RIF, microscopy, and culture tests were done in parallel.

In the case of sputum samples, pellets were used for GeneXpert MTB/RIF assay. Sample reagent (1.5 ml) was added to 0.5 ml of the re-suspended sputum pellet and manually agitated twice at room temperature during a 15-minutes period.

In the case of other clinical body fluids, we transferred the entire specimen to a conical centrifuge tube, and concentrate the specimen at 3000 g for 15 minutes , carefully poured off the supernatant through a funnel into a discard can containing 5% sodium hypochlorite, re-suspend the deposit to a final volume of 2 ml by Phosphate Buffer Saline (PBS) , using transfer pipette, we added a double volume of the GeneXpert MTB/RIF sample Reagent (1.4 ml) to 0.7 ml (2:1) of suspension, then 2 ml transferred to the concentrate and load to the GeneXpert MTB/RIF cartridge. The GeneXpert MTB/RIF purifies *M. tuberculosis* from these clinical samples. The

genetic content of *M. tuberculosis* captured and subsequently amplifies the genomic DNA by polymerase chain reaction (PCR). In addition, it identifies RIF's resistance mutations in the RNA polymerase beta (rpoB) gene of *M. tuberculosis* in all clinically important samples within 2 hours [11].

Regarding microscopic examination, all sputum smears are prepared from decontaminated and concentrated specimens. The smears stained with Ziehl-Neelsen (ZN) staining techniques, could be used to count both viable and non-viable bacilli as acid-fast bacilli (AFB).

This method uses a carbolfuchsin as primary stain, acid alcohol as decolorizer, and methylene blue as counterstain. Acid-fast organisms stain red, while the background of debris stains blue. The ZN stain confirms the acid-fast property of mycobacteria using microscopy examination. Bacillary density will be graded as scanty, 1+, 2+, and 3+, and all such smears will be defined as ''smear-positive''.

Lowenstein-Jensen (LJ) culture medium was used which incorporates congo red and malachite green to inhibit unwanted bacteria for culturing. Once good growth was obtained, the positive slants were stored in a cool, dark place to archive the positive *M. tuberculosis* isolates.

Data Quality Assurance

The questionnaire was pre-tested and proper training prior to the actual data collection was given for data collectors. The necessary adjustments were made after the pre-test. The quality of data was maintained through strictly following the pre-analytical, analytical and post-analytical steps through each day supervision and SOPs of the laboratory procedures.

Data analysis and interpretation

The collected data were entered to EPI info 2002 version 3.32 after data cleaning it was exported to SPSS version 23 windows software computer program for analysis. The logistic regression

was employed to assess the association between TB and its risk factors. A p-value of less than 0.05 was considered as statistical significance.

Ethical considerations

This study was approved by Department of Medical Laboratory Science, College of Health Sciences, Addis Ababa University, Addis Ababa, Ethiopia. Then official permission was obtained from Institutional Review Board (IRB) of St. Paul's Hospital Millennium Medical College, Addis Ababa, Ethiopia, and then submitted to laboratory department. Written informed consent was secured from each participant greater than 18 years old and assents were obtained for those less than 18 years old. Patients' names were not recorded on the questionnaire to guarantee confidentiality of the information and privacy of the patients. Infected patients and/or those who had resistance *M. tuberculosis* were informed to their health care provider for better care and management.

Operational Definition

MDR-TB: is non-susceptibility of *M. tuberculosis* at least two first line TB drugs (isoniazid and rifampicin).

Presumptive TB: a patient who presents with symptoms or signs suggestive of TB including cough >2week, fever >2week, significant weight loss, haemoptysis, any abnormality chest radiograph.

Presumptive MDR-TB: smear positive previously treated patients who define as relapse, return after default, and failure; new smear positive pulmonary TB patients who sputum remains smear positive at month 2 or 3 of treatment.

Results

Socio-demographic characteristics

A total of 436 participants were enrolled during the study period, of this 223 (51%) were male. The mean ± SD age the participants were 38±17years. The highest age range was 35-49 years old, while the youngest was under 15 years old. Majority of the respondents were 240 (55%) urban residents, and 214 (49%) had monthly income of 100-1000 Ethiopian Birr (Table 1).

Clinical data

There were a total of 374 (85.8%) patients with pulmonary tuberculosis and 62 (14.2%) people with extra-pulmonary tuberculosis, with 130 (30%) of them being HIV positive. Presumptive TB was discovered in 422 (96.8%) of the individuals, while presumptive DRTB was found in 14 (3.2%) of the total participants.

In this study 33(7.5%) of the participants had history of contact with a TB patient, 68(15.6%) had history of alcohol drinking, and 22 (5%) were cigarette smokers. Among the study participants 319 (73.1%) had fever, 311 (71.3%) had night sweating, and 365(83.7%) had cough. Out of 130 HIV positive participants, 104 (81%) were on anti-HIV treatment and were monitored based on their $CD4^+$ T-cells count. In addition, 119 (91.5%) participants were tested for HIV viral load. Higher magnitude *M. tuberculosis* seems to be appeared for those who have $CD4^+$count 200-350/mm^3 (5/34) and viral load ≥1000/mm^3 (6/90) (Table 2).

Magnitude of *M. tuberculosis* and its resistance pattern

Out of the total participants of clinical specimen, *M. tuberculosis* was detected in 36(8.3%) of the samples with GeneXpert MTB/RIF, and of which only 2 (0.5 %) of them were RIF resistant. Regarding culture result, 27(6.2%) of the participants were confirmed for M. tuberculosis infection and one *M. tuberculosis* strain was resistant for Isozianide drug (mono-resistant) and 2 were resistant for Isozianide and RIF (Multidrug resistant TB).

Factors associated to *M. tuberculosis*

The bivariate logistic regression analysis of socio-demographic characteristics revealed that participants under the age of 15 years old were 1.8 times (95% CI: 0.4, 8.1) more likely to develop M. tuberculosis than those over 50 years old. Widowed participants were 2.6 times (95% CI: 0.4, 17) more likely to have *M. tuberculosis* than unmarried ones, and government workers were 1.8 times (95% CI: 0.6, 5.9) higher than housewives (Table 3). On bivariate logistic analysis, contact history with tuberculosis-infected patients, pneumonia confirmed by chest X-ray examination, and $CD4^{+}$results were associated factors for *M. Tuberculosis*; however, none of these factors were associated in the multivariate analysis (Table 4).

Discussion

People aged 35 to 49 years old, as well as those living in families with 4-6 members, had the highest frequency of tuberculosis. In terms of occupation, laborers earning between 1000 and 2000 Ethiopian Birr per month were the most susceptible to tuberculosis. This could be because these age groups are more likely to be subjected to high workloads and have a greater range of motion.

The current study found that as the number of people living together increases (5-6 family size), *M. tuberculosis* positivity increases as well. Other studies have found that having a larger family and malnutrition contribute to the development of tuberculosis [12], however, the present study found no association between family size/household and *M. tuberculosis*.

Higher *M. tuberculosis* was detected among participants in presumptive diagnosis of tuberculosis 25/436 (5.7%), non-vaccinated for BCG 18/436 (4.1%) than vaccinated, non-alcoholic drinkers 21/436 (4.8%) than alcoholic drinkers, and non-cigarette smokers 25/436 (5.7%) than smokers.

Again, among symptomatic tuberculosis patients, higher *M. tuberculosis* results were observed in those with night sweating 23/436 (5.2%), fever 22/436 (5.0%), weight loss 20/436 (4.5%), cough 24/436 (5.5%), loss of appetite 20/436 (4.5%), and chest pain 16/436 (3.7%) had . The lowest results were reported in those patients with dyspnea 9/436 (2.0%), diarrhea 3/436 (0.7%), and palpable lymphadenopathy 3/436 (0.7%).

Although tuberculosis has been frequently observed in people who have had a history of contact with a diseased person who has already developed a cough, as well as cigarette smokers and alcohol users, our data suggest that these people are not significantly more likely to develop tuberculosis. These findings were different from the studies done in Addis Ababa, Ethiopia in 2011 and north Gondar in 2015 [13, 14].

The possible reason could be the lower number of participants diagnosed with presumptive DRTB and the fact that most participants live in an urban area. Higher results were observed in patients who had previously been treated with anti-TB medications (20/436, or 4.5%), as well as new patients with a presumptive diagnosis of drug-resistant tuberculosis 24/436 (5.5%).

There was a statistically significant link between culture-positive pulmonary tuberculosis and TB contact history, pneumonia, and CD 4^+ counts, as well as several tuberculosis patient symptoms such as weight loss. The earlier study also found a link between pulmonary tuberculosis and the number of $CD4^+$ cells in HIV patients and the amount of virus in their blood [13, 15].

The current result seems similar with reports of study conducted in Addis Ababa, Ethiopia in 2017 [16], prisons settings of East Gojjam Zone, Northwest Ethiopia using GeneXpert MTB/RIF, 9(3.4%) [17] and 9.9 % of the study conducted in extra pulmonary tuberculosis at University of Gondar, Northwest Ethiopia [18]. This overall culture confirmed *M .tuberculosis*, 27/436(6.2%) magnitude is lower than the study conducted in the Health Centers of Addis

Ababa, Ethiopia reported as 46.0% (233/506) [13], from Metehara sugar factory hospital, eastern Ethiopia (14.2%) and 124 (32.2%) of studied in two public hospitals in East Gojjam zone, northwest Ethiopia [19].

We detected a reduced prevalence of tuberculosis (24.6%) when compared to a retrospective study report from the University of Gondar Hospital from January 2013 to August 2015 [20]. Our results were also lower than those of a study conducted in Debre Markos Referral Hospital in Ethiopia, which found a prevalence of 23.2 % utilizing the GeneXpert MTB/RIF assay.

The difference could be due to the different diagnostic methods we used; for example, in our cases, we used the sputum sedimentation concentration technique for microscopic smear examination, GeneXpert MTB/RIF assay, and finally, LJ culture for confirmation, whereas in previous studies, a single diagnostic tool was used, such as stained by Ziehl-Neelsen staining and examined by Microscopy in Metehara [18], GeneXpert MTB/RIF in prisons settings of East Gojjam Zone [17]. This low prevalence could also indicate that TB infection control in our study area, Addis Ababa, Ethiopia, is relatively good.

From the overall confirmed *M. tuberculosis* 6.2% (27/436), a total of three *M. tuberculosis* strain showed resistance pattern to anti-tuberculosis drug, of which two of them were multi drug (INH and RIF) resistance strains. This result was lower than the study conducted in the University of Gondar Hospital, northwest Ethiopia which was reported as 71(15%) of tuberculosis-presumptive cases were resistant to rifampicin [20] ,and 15.58 % of two public hospitals in East Gojjam zone, northwest Ethiopia [19], and 12 (10.3%) patients referred to Debre markos Referral Hospital, Ethiopia [21].

Only 10(7.7%) of the 130 HIV-infected patients tested positive for MTB, one mono (INH) resistant and one MDR-TB (INH+RIF) resistant strain were found in this seropositive figure.

In terms of viral load and tuberculosis, only one mono resistant strain was discovered in the participant serum, which has a high viral load count (1000/mmm3). This could be linked to HIV infection, which causes anti-TB drug mal-absorption and immunological suppression, leading to resistance, and our findings are backed up by previous research [22, 23].

According to the bivariate logistic analysis, patients with a presumptive diagnosis of drug resistance were two times more likely 2.6 times (95 % CI 0.6, 12, p=0.2) to acquire tuberculosis than those with a presumptive diagnosis of tuberculosis. Patients who also had night sweating were two times more likely to get tuberculosis 2.4 times (95% CI 0.8, 7.2, p=0.1) than those who did not have. When compared to patients who did not have chest pain, having chest pain was also associated with had 1.6 times, (95%t CI 0.8, 3.7, p=0.2) greater risk of getting *Mycobacterium tuberculosis.*

Conclusion

In general, this study found low-magnitude *M. tuberculosis* in patients with presumptive diagnosis of TB at SPHMMC in Addis Ababa, Ethiopia. And three DRTB strains, including two MDR strains, were discovered in individuals with a history of failure, relapse, and previous anti-TB treatment.

Contact with tuberculosis-infected patients, patient weight loss, pneumonia on radiological examination, and low CD4+ levels were all found to be linked with *M. tuberculosis*. To maintain this low illness outcome, health education on tuberculosis, TB control programs, and large community-based studies should be continued. To lessen the incidence of MDR-TB, it is also advised that TB infection control activities be strengthened and DOT be properly implemented.

Abbreviation

ATCC=American Type Culture Collection, BCG=Bacillus Calmette–Guérin, DOT= Directly Observed Therapy, DST= Drug Susceptibility Test, FMOH=Federal Ministry of Health, HIV=Human Immunodeficiency Virus, INH=Isoniazid, IQC=Internal Quality Controls, MDR-TB=Multidrug-Resistant Tuberculosis, MOTT= Mycobacteria other than TB, MTB= *Mycobacterium Tuberculosis*, OADC = Oleic Acid Albumin Dextrose Complex, PPE=Personal protective equipment, PTB= Pulmonary Tuberculosis, RIF=Rifampicin, RMR= Rifampicin Mono-Resistant, SOP= Standard Operating Procedures, SPHMMC= Saint Paul's Hospital Millennium Medical College, SR=sample reagent, STM=Streptomycin, TB=Tuberculosis, TTD= Time to detection, WHO= World Health Organization.

Declaration

Consent for publication

Not applicable. This study does not contain any individual or personal data.

Availability of data and materials

All data relevant to this study are available on the manuscript.

Authors' contributions

MK, KD, RA, ZG, AGe, NZ, MA, BZ, MG and AGi contributed to the study's conceptualization, data gathering and analysis, and manuscript writing. The manuscript was critically examined for intellectual content by MK, KD, and AGi. The manuscript has been read, modified, and approved by all authors.

Acknowledgement

The authors would like to express their gratitude to the staff of the Departments of Medical Laboratory Science, College of Health Sciences, Addis Ababa University, and the Department of National Tuberculosis Reference Laboratory, as well as the management of St. Paul's Hospital

Millennium Medical College (SPHMMC). Finally, we thank all study participants. Last but not least, authors appreciated Dr. Yoseph Solomon Bezabih for his contribution of revising English language and maximizing the readability of the manuscript.

Competing interests

The authors declare that they have no competing interests.

References

1. Gelaw SM. Socioeconomic factors associated with knowledge on tuberculosis among adults in Ethiopia. Tuberc Res Treat. 2016; 2016:6207457. doi: 10.1155/2016/6207457. Epub 2016 Feb 1. PMID: 26949546; PMCID: PMC4753341

2. World Health Organization. Global tuberculosis report 2021. https://www.who.int/publications/i/item/9789240037021 (Accessed on November 08, 2021).

3. Global Tuberculosis Report 2019. Geneva: World Health Organization; 2019. https://www.who.int/publications/i/item/9789241565714

4. Girum T, Muktar E, Lentiro K ,Wondiye H and Shewangizaw M. Epidemiology of multidrug-resistant tuberculosis (MDR-TB) in Ethiopia: a systematic review and meta-analysis of the prevalence, determinants and treatment outcome. Tropical Diseases, Travel Medicine and Vaccines. 2018 Jun 14;4:5. doi: 10.1186/s40794-018-0065-5. PMID: 29942536; PMCID: PMC6000958.

5. Asgedom SW, Tesfaye D, Nirayo YL, Atey TM. Time to death and risk factors among tuberculosis patients in Northern Ethiopia. BMC Res Notes. 2018;11(1):696. doi: 10.1186/s13104-018-3806-7. PMID: 30286801; PMCID: PMC6172746.

6. Atalell KA, Tebeje NB, Ekubagewargies DT. Survival and predictors of mortality among children co-infected with tuberculosis and human immunodeficiency virus at University of Gondar Comprehensive Specialized Hospital, Northwest Ethiopia. A retrospective follow-up study. PLoS One. 2018 May 22;13(5):e0197145. doi: 10.1371/journal.pone.0197145. PMID: 29787596; PMCID: PMC5963769.

7. Woya AA, Tekile AK, Basha GW. Spatial Frailty Survival Model for Multidrug-Resistant Tuberculosis Mortality in Amhara Region, Ethiopia. Tuberc Res Treat. 2019 Jan 1;2019:8742363. doi: 10.1155/2019/8742363. PMID: 30693105; PMCID: PMC6332868.

8. Deribew A, Deribe K, Dejene T, Tessema GA, Melaku YA, Lakew Y, Amare AT, Bekele T, Abera SF, Dessalegn M, Kumsa A, Assefa Y, Kyu H, Glenn SD, Misganaw A, Biadgilign S. Tuberculosis Burden in Ethiopia from 1990 to 2016: Evidence from the Global Burden of Diseases 2016 Study. Ethiop J Health Sci. 2018 Sep;28(5):519-528. doi: 10.4314/ejhs.v28i5.2. PMID: 30607066; PMCID: PMC6308773.

9. Piatek AS, Cleeff MV, Alexander H, Coggin WL, Rehr M, Kampen SV, et al. GeneXpert for TB diagnosis: planned and purposeful implementation. Glob Health Sci Pract. 2013;1(1):18–23. doi: 10.9745/GHSP-D-12-00004. PMID: 25276513; PMCID: PMC4168558.

10. Steingart KR, Sohn H, Schiller I, Kloda LA, Boehme CC, Pai M, et al. Xpert® MTB/RIF assay for pulmonary tuberculosis and rifampicin resistance in adults. Cochrane Database Syst Rev. 2013 Jan 31;(1):CD009593. doi: 10.1002/14651858.CD009593.pub2. Update

in: Cochrane Database Syst Rev. 2014;(1):CD009593. PMID: 23440842; PMCID: PMC4470352.

11. Atashi S, Izadi B, Jalilian S, Madani SH, Farahani A, Mohajeri P. Evaluation of GeneXpert MTB/RIF for determination of rifampicin resistance among new tuberculosis cases in west and northwest Iran. New Microbes New Infect. 2017 ;19:117–20. doi: 10.1016/j.nmni.2017.07.002. PMID: 28794886; PMCID: PMC5547232.

12. Zenebe Y, Anagaw B, Tesfay W, Debebe T, Gelaw B. Smear positive extra pulmonary tuberculosis disease at University of Gondar Hospital, Northwest Ethiopia. BMC Res Notes. 2013 ;6(1):21. doi: 10.1186/1756-0500-6-21. PMID: 23331864; PMCID: PMC3558382.

13. Deribew A, Negussu N, Melaku Z, Deribe K. Investigation Outcomes of Tuberculosis Suspects in the Health Centers of Addis Ababa, Ethiopia. PLOS ONE. 2011 ;6(4):e18614. doi: 10.1371/journal.pone.0018614. PMID: 21526179; PMCID: PMC3079716.

14. Mekonnen F, Tessema B, Moges F, Gelaw A, Eshetie S, Kumera G. Multidrug resistant tuberculosis: prevalence and risk factors in districts of metema and west armachiho, Northwest Ethiopia. BMC Infect Dis. 2015 ;15(1):461. doi: 10.1186/s12879-015-1202-7. PMID: 26503269; PMCID: PMC4624367.

15. Lawn SD, Badri M, Wood R. Tuberculosis among HIV-infected patients receiving HAART: long term incidence and risk factors in a South African cohort. AIDS. 2005 ;19(18):2109 -16. doi: 10.1097/01.aids.0000194808.20035.c1. PMID: 16284460.

16. Nugussie DA, Mohammed GA, Tefera AT. Prevalence of Smear-Positive Tuberculosis among Patients Who Visited Saint Paul's Specialized Hospital in Addis Ababa, Ethiopia. Biomed Res Int. 2017;2017:6325484. doi: 10.1155/2017/6325484. Epub 2017 Aug 21. PMID: 28904965; PMCID: PMC5585560.

17. Gizachew Beza M, Hunegnaw E, Tiruneh M. Prevalence and associated factors of tuberculosis in prisons settings of East Gojjam Zone, Northwest Ethiopia. Int J Bacteriol. 2017;2017 :3826980. doi: 10.1155/2017/3826980. Epub 2017 Oct 17. Erratum in: Int J Bacteriol. 2018 Aug 9; 2018:1020349. PMID: 29226216; PMCID: PMC5684609.

18. Yohanes A, Abera S, Ali S. Smear positive pulmonary tuberculosis among suspected patients attending metehara sugar factory hospital; eastern Ethiopia. Afr Health Sci. 2012 ;12(3):325-30. doi: 10.4314/ahs.v12i3.12. PMID: 23382747; PMCID: PMC3557681.

19. Adane K, Ameni G, Bekele S, Abebe M, Aseffa A. Prevalence and drug resistance profile of *Mycobacterium tuberculosis* isolated from pulmonary tuberculosis patients attending two public hospitals in East Gojjam zone, northwest Ethiopia. BMC Public Health. 2015 ;15(1):572. doi: 10.1186/s12889-015-1933-9. PMID: 26092570; PMCID: PMC4473837.

20. Jaleta KN, Gizachew M, Gelaw B, Tesfa H, Getaneh A, Biadgo B. Rifampicin-resistant Mycobacterium tuberculosis among tuberculosis-presumptive cases at University of Gondar Hospital, northwest Ethiopia. Infect Drug Resist. 2017 ;10:185–92. doi: 10.2147/IDR.S135935. PMID: 28652786; PMCID: PMC5476602.

21. Mulu W, Abera B, Yimer M, Hailu T, Ayele H, Abate D. Rifampicin-resistance pattern of Mycobacterium tuberculosis and associated factors among presumptive tuberculosis patients referred to Debre Markos Referral Hospital, Ethiopia: a cross-sectional study. BMC Res Notes. 2017 ;10(1):8. doi: 10.1186/s13104-016-2328-4. PMID: 28057041; PMCID: PMC5217448.

22. Wells CD, Cegielski JP, Nelson LJ, Laserson KF, Holtz TH, Finlay A, et al. HIV Infection and Multidrug-Resistant Tuberculosis—The Perfect Storm. J Infect Dis. 2007 ;196(Supplement_1):S86–107. doi: 10.1086/518665. PMID: 17624830.

23. Getahun H, Gunneberg C, Granich R, Nunn P. HIV Infection—Associated Tuberculosis: The Epidemiology and the Response. Clin Infect Dis. 2010 ;50(Supplement_3):S201–7. doi: 10.1086/651492. PMID: 20397949.

List of tables

Table 1: Socio-demographic characteristics among presumptive TB patients at SPHMMC, Addis Ababa, Ethiopia, 2019

Variables/ characteristics		No. of Participants	Percentages (%)
Sex	Male	224	51
	Female	212	49
	<15 years	39	9
	15-24 years	56	13
	25-34 years	98	22

Age groups	35-49 years	127	29
	>50 years	116	27
Residence	Urban	240	55
	Rural	196	45
Family size/house	1-3	152	35
	4-6	220	50
	>6	64	15
Marital status	Single	146	33
	married	238	55
	Divorced	20	5
	Widowed	32	7
Occupational status	Laborer	97	22
	Government workers	97	22
	Private workers	109	25
	House wife	70	16
	Student	63	15
Educational status	No formal Education	119	27
	1-8th grades	147	34
	9-12th grades	106	24
	>12th grade	64	15
	<100 Birr	60	14
	100-1000 Birr	83	19
	1001-2000 Birr	155	35.

	2001-3000 Birr	59	14
Monthly Income	3001-4000 Birr	32	7
	4001-5000 Birr	25	6
	>5001 Birr	22	5.0

Table 2: Clinical characteristics participants among presumptive TB patients at SPHMMC, Addis Ababa, Ethiopia, 2019

Variables/ Characteristics		Number of participants	Percentages (%)
Reason for diagnosis	Presumptive TB	422	97
	Presumptive DRTB	14	3
BCG vaccination	Vaccinated	156	36
	Non-Vaccinated	280	64
TB contact history	Yes	33	8
	No	403	92
Alcohol drinking	Yes	68	16
	No	368	84
Cigarette smoking	Smokers	22	5
	Non-smokers	414	95
Night sweating	Yes	310	71
	No	126	29
Presence of fever	Yes	318	73
	No	118	27

Weight loss	Yes	200	46
	No	236	54
Presence of Cough	Yes	364	83
	No	72	17
Loss of appetite	Yes	285	65
	No	151	35
Presence of chest pain	Yes	206	47
	No	230	53
Presence of diarrhea	Yes	57	13
	No	379	87
Presence of dyspnea	Yes	140	32
	No	296	68
External-adenopathy	Yes	63	14
	No	373	86
Anti-TB Treatment	Previously treated	110	25
	Previously untreated	326	75
Presumptive DRTB	New	384	88
	Relapse	46	11
	Failure	6	1
HIV status	Positive	130	30
	Negative	306	70
Tuberculosis type	PTB	373	86
	EPTB	63	14

	<200 cells/mm^3	16	16
CD4$^+$ count	200-350/mm^3	34	33
	>350/mm^3	53	51
	<1000/ mm^3	29	24
HIV viral load	≥1000/ mm^3	90	76

BCG= Bacillus Calmette–Guérin, DRTB= Drug Resistance Tuberculosis, EPTB=Extra Pulmonary Tuberculosis HIV=Human Immunodeficiency Virus, MDR-TB=Multidrug-Resistant Tuberculosis, MTB= Mycobacterium Tuberculosis, PTB=Pulmonary Tuberculosis

Table 3**:** Socio-demographic factor analysis of *M. Tuberculosis* among presumptive TB patients at SPHMMC, Addis Ababa, Ethiopia, 2019

Variables/ characteristics		#M.TB Not detected (%)	#M.TB Detected (%)	#Total (%)	COR (95% CI)	P-value
Sex	Male	208 (93)	16 (7)	224 (51)	1.4(0.6-3.1)	0.4
	Female	201 (95)	11 (5)	212 (49)	1	
Age groups	<15 years	36 (92)	3 (8)	39 (9)	1.8(0.4, 8.1)	0.41
	15-24 years	52 (93)	4 (7)	56 (13)	1.7(0.4, 6.6)	0.44
	25-34 years	92 (94)	6 (6)	98 (22)	1.5(0.4, 4.9)	0.55
	35-49 years	118 (93)	9 (7)	127 (29)	1.7(0.6, 5.2)	0.36
	>50 years	111(96)	5 (4)	116 (27)	1	

Residence	Urban	227 (95)	13 (5)	240 (55)	1	
	Rural	182 (93)	14 (7)	196 (45)	1.4(0.6, 2.9)	0.4
Family size/house	1-3	144 (95)	8 (5)	152 (35)	1	
	4-6	204 (93)	16 (7)	220 (50)	1.4(0.6, 3.4)	0.4
	>6	61 (95)	3 (5)	64 (15)	0.9(0.3, 3.5)	0.8
Marital status	Single	136 (93)	10 (7)	146 (33)	1	
	Married	226 (95)	12 (5)	238 (55)	1(0.2, 5.3)	0.9
	Divorced	17 (85)	3 (5)	20 (5)	0.8(0.2, 3.7)	0.7
	Widowed	30 (94)	2(9)	32 (7)	2.6(0.4, 17)	0.3
Occupational status	Laborer	89 (92)	8 (8)	97 (22)	1.4(0.4, 4.6)	0.6
	Government workers	91(94)	6 (6)	97 (22)	1.8(0.6, 5.9)	0.3
	Private workers	58 (92)	5 (8)	63 (15)	1.7(0.4, 6.4)	0.4
	Student	67 (96)	3 (4)	70 (16)	0.9(0.2, 4.0)	0.9
	House wife	104 (95)	5 (5)	109 (25)	1	
Educational status	Illiterate	112 (94)	7 (6)	119(72)	0.7(0.3, 2.4)	0.6
	1-8th grades	140 (95)	7 (5)	147(34)	0.6(0.2, 1.9)	0.4
	9-12th grades	98 (92)	8 (8)	106(24)	0.9(0.3, 3.0)	0.9
	>12th grade	59 (92)	5 (8)	64 (15)	1	
	<100 Birr	56 (93)	4 (7)	60 (14)	1.4(0.2, 13)	0.8
	100-1000 Birr	79 (95)	4 (5)	83 (19)	1(0.1, 9)	0.9
	1001-2000 Birr	147 (95)	8 (5)	155(36)	1(0.3, 9)	0.9
	2001-3000 Birr	54 (92)	5 (8)	59 (14)	1.8(0.2, 16)	0.6

Monthly Income	3001-4000 Birr	32 (100)	0 ()	32 (7)	-	0.9
	4001-5000 Birr	20 (80)	5 (20)	25 (6)	5(0.5, 46)	0.2
	>5001 Birr	20 (91)	2 (9)	22 (5)	1	

N.B: BCG= Bacillus Calmette–Guérin, COR= Crude Odds Ratio, DRTB= Drug Resistance Tuberculosis, HIV=Human Immunodeficiency Virus, MDR-TB=Multidrug-Resistant Tuberculosis, MTB= Mycobacterium Tuberculosis, TB=Tuberculosis

Table 4: Clinical factors associated with Magnitude of *M. tuberculosis* among presumptive TB patients at SPHMMC, Addis Ababa, Ethiopia, 2019

**Variables/ characteristics**		**#M. TB not detected (%)**	**#M.TB detected (%)**	**#Total (%)**	**COR (95% CI)**	**P-value**
Reason for Diagnosis	Presumptive TB	397 (94)	25 (6)	422 (97)	1	
	Presumptive DRTB	12 (86)	2 (14)	14 (3)	2.6(0.6, 12)	0.2
BCG Vaccination	Vaccinated	147 (94)	9 (6)	156 (36)	1	
	Non-Vaccinated	262 (94)	18 (6)	280 (64)	1.1(0.5, 2.6)	0.7
TB contact History	Yes	28 (85)	5 (15)	33 (8)	3.1(1.1, 8.7)	0.03
	No	381 (95)	22 (5)	403 (92)	1	
Alcohol Drinking	Yes	62 (91)	6 (9)	68 (16)	1.6(0.6, 4.1)	0.3
	No	347(94)	21(6)	368 (84)	1	
Cigarette smoking	Smokers	20 (91)	2 (9)	22 (5)	1.6(0.3,7.0)	0.5
	Non-smokers	389 (94)	25 (6)	414 (95)	1	
Chest X-ray	Pneumonia	25 (89)	3 (11)	28 (7)	3(33, 319)	0.02
	Interstitial	28 (90)	3 (10)	31(7)	3(0.3, 30)	1.0
	Bronchiectasis	11(92)	1 (8)	12 (3)	2.6(0.3, 27)	0.34

	Bilateral	6 (43)	8 (57)	14 (3)	9(0.9, 8)	0.4
	Unilateral	14 (74)	5 (24)	19 (4)	0.5(0.6, 4.3)	0.5
	Normal	324 (98)	7 (2)	331(76)	1	
	Untreated	103 (94)	7(6)	110 (25)	1	
Anti-TB treatment	Previously treated	306 (94)	20 (6)	326 (75)	1.1(0.4, 2.5)	0.9
	New	362 (94)	24 (6)	386 (89)	1	
Presumptive DRTB	Relapse	44 (91)	2 (9)	46 (11)	0.7(0.2, 3)	0.6
	Failure	3 (75)	1(25)	4 (1)	5.0(0.5, 5.0)	0.2
	Positive	120 (92)	10 (8)	130 (30)	1.4(0.6, 3.1)	0.4
HIV status	Negative	289 (94)	17 (6)	306 (70)	1	
CD4 count/ mm^3 blood	<200	16 (100)	0 (0)	16 (15)	1.2(0.9, 2.4)	0.9
	200-350	29 (85)	5 (15)	34 (33)	8.9(0.5, 0.9)	0.049
	≥350	52 (96)	2 (4)	54 (52)	1	
Viral Load /mm^3 blood	<1000	27 (93)	2 (7)	29 (24)	1	
	≥1000	84 (93)	6 (7)	90 (76)	0.9(0.2, 5.0)	0.9

BCG= Bacillus Calmette–Guérin, CD= Cluster of Differentiation, Crude Odds Ratio, DRTB= Drug Resistance Tuberculosis, HIV=Human Immunodeficiency Virus, MDR-TB=Multidrug-Resistant Tuberculosis, MTB= Mycobacterium Tuberculosis, TB=Tuberculosis

Click here to access/download
Supporting Information
Supporting Files_ Questionnaire.docx

Magnitude of ~~Multidrug Resistance~~ *Mycobacterium tuberculosis*, *Drug resistance* and associated factors among presumptive patients at St. Paul's Hospital Millennium Medical College, Addis Ababa, Ethiopia

Melkayehu Kassa[1], Kassu Desta[2], Rozina Ambachew[1], Zenebe Gebreyohannes[1] ~~Youhannes[1]~~, Alganesh ~~Yohannes~~Gebreyohanns[1], Nuhamen Zena[1], Misikir Amare[3], Betselot Zerihun[3], Melak~~u~~ Getu[3] and Addisu Gize[1*]

[1]St.Paul's Hospital Millennium Medical College, Addis Ababa, Ethiopia.

[2]College of Health Science, Addis Ababa University, Addis Ababa, Ethiopia

[3]Ethiopian Public Health Institute, Addis Ababa, Ethiopia.

Authors' Email:

Melkayehu Kassa= kassamelkayehu@gamil.com

Kassu Desta= kassudesta2020@gmail.com

Rozina Ambachew= nanieyasu@gmail.com

Zenebe Youhannes= zenebezoha@gmail.com

Alganesh Yohannes= alganesh5838@gmail.com

Nuhamen Zena= nuhamenzf@gmail.com

Misikir Amare= misikiramare.ma@gmail.com

Betselot Zerihun= betselotzerihun3@gmail.com

Melak~~u~~ Getu= melakgetu2005@gmail.com

Addisu Gize= addisu.gize@sphmmc.edu.et

*Corresponding author= ~~Addisu Gize,~~ Email: addisu.gize@sphmmc.edu.et , or konjoaddisu@gmail.com (AG)

Formatted: Superscript

Abstract

Background: *Mycobacterium tuberculosis* (*M. tuberculosis*) remains one of the most significant causes of death and a major public health problem in the community.

Therefore, the aimgoal of this study was to determine magnitude of Multi Drug *Mycobacterium tuberculosis*, its drug resistance (MDR-TB) and its associated factors among TB presumptive *M.Tuberculosis* (MTB) patients at St. Paul's Hospital Millennium Medical College, Addis Ababa, Ethiopia.

Methods: CA cross-sectional study was usedconducted at St. Paul's Hospital Millennium Medical College (SPHMMC), Addis Ababa, Ethiopia from January to July 2019. Socio-dDemographic and dataclinical data were collected by using structured questionnaire throughin face to face interview with patients. Pulmonary and extra-pulmonary Sputum samplesclinical samples were collected and the laboratory analysis was done by microscopic examinationy and GeneXpert MTB/RIF X-pert MTB/RIF assay and culturing in the Lowenstein-Jensen (LJ) culture media. Data were analyzed by SPSS version 23. Binary logistic regression was done to identify the associated risk factors,factors and p-value less than 0.05 was taken as significant association.

Results: Of the total of 436 respondents, 223 (51%) were male. The mean ±SD age the participants were 38±17years. Out of the total participants, the overall confirmed *Mycobacterium tuberculosis* was through GeneXpert MTB/RIF X-pert MTB/RIF assay and LJ culture media was 27/436 (6.2%), and twothree isolates were resistant for either INH or RIF and one for INH drug, of which while two of them (0.5%) were MDR-TB based on line probe assays method. Previous TB-contact history, patient weight loss, having pneumonia with chest X-ray finding, and $CD4^{+}$ T-cells count 200-350/mm^3 of blood were significantly associated predictors for MTB infection.

Formatted: Font: Not Italic

Formatted: Font: Not Italic

Formatted: Font: Italic

Commented [A1]: Copy of _AFHS1203-0325(1)

Formatted: Highlight

Formatted: Highlight

Formatted: Highlight

Formatted: Highlight

Formatted: Highlight

Formatted: Highlight

Formatted: Highlight

Formatted: Highlight

Formatted: Highlight

Formatted: Highlight

Formatted: Highlight

Formatted: Font: Not Italic

Conclusion: The magnitude of *M. tuberculosis* and MDR-TB in this study highlights the need for further extended early case detection and managing MDR-TB cases to minimize transmission and the suffering of patients.

Key words: *M. tuberculosis*, *GeneXpert MTB/RIF ~~X-Pert~~*, *Lowenstein-Jensen (LJ)*, Associated Factors, Addis Ababa.

Background

Tuberculosis (TB) is a communicable ~~n infectious~~ disease acquired ~~caused by~~ from ~~strains belonging to the~~ *Mycobacterium tuberculosis* complex. The main transmission is through~~It is transmitted by~~ respiratory route from the infected patient during ~~when a patient i~~s coughing or sneezing near to healthy individuals, however,~~and one strain of TB~~, *Mycobacterium bovis*, could infect while we are eating and drinking of unboiled milk of the infected cattle~~can be caused by drinking not boiled milk~~ [1]. World Health Organization (WHO) estimated that 9.9~~10~~ million people developed tuberculosis (TB) and 1.5~~6~~ million died of TB globally in 2020 ~~2017~~ and 22% of the world ~~one fourth of~~ people infected with latent *Mycobacterium tuberculosis*, of which 95% cases occur in resource limited settings [2].

Worldwide, in 2019 WHO report estimate that 3.4% of the prevalence of MDR-TB among the new patients, and 18% of previously treated cases had MDRTB [3].

Accordingly, this report estimate the incidence TB in Ethiopia 247 per 100,000 and considered the country ranked as the 7th from the world and 4th fourth from Sub-Saharan African high TB-burdened countries [3,4].

Formatted: Not Highlight

Formatted: Not Highlight

Formatted: Font: Italic

Formatted: Not Highlight

Formatted: Font: Not Italic

Formatted: Line spacing: Double

Formatted: Not Highlight

The epidemiology of MDR-TB in the country by conducted by 2018 confirm an overall prevalence of 7.24% of which, 2.1% from new and 21.07 % retreatment cases in Ethiopia [4].

~~Globally, the estimated prevalence of MDR-TB was 3.3% in newly diagnosed patients in the WHO 2015 report. This was higher to 20% in patients with a history of anti-TB treatment(30)~~.

~~A global TB report estimated that there were about 230,000 (247 per 100,000 population) incident cases of TB in Ethiopia.~~

~~Ethiopia ranks seventh among the world's 22 high-TB-burden countries, 10th among high-TB-pandemic countries, and fourth in sub-Saharan Africa [3].~~

~~Based on the 2005 nationwide survey in Ethiopia, the prevalence of MDR-TB was 1.6% among new cases and 11.8% in the retreatment cases and rifampicin resistant was lower than 2% in new cases [4].~~

High mortality rate was observed in different health institution of the Northern Ethiopia; 87 (11.3%) patients died in Mekelle Hospital and Ayder Comprehensive Hospital [5], 38 (14.02%) children from TB/HIV co-infected University of Gondar Comprehensive Specialized Hospital [6] and from ~~multidrug resistant tuberculosis (~~MDR-TB~~)~~ data showed that 61(29.47%) of the patients died in different hospitals of Amhara region, Northwest Ethiopia [7]. Generally in Ethiopia, TB mortality rate declined from 393.8/100,000 to 100/100,000 between 1990 and 2016 (with a total decline of 75%), which indicates slow decline and resulted males had higher TB mortality rate than females [8].

The common microscopic examination accuracy for *M. tuberculosis* in sputum stained smear difference ranged from 20% in the light microscope up 80% in the case of fluorescence method applications.

Formatted: Space Before: 0 pt, After: 10 pt, Adjust space between Latin and Asian text, Adjust space between Asian text and numbers

Field Code Changed

Formatted: Font: (Default) Times New Roman, 12 pt

Sputum smear microscopy remains the most common way to diagnose pulmonary TB. Depending on the report and method used, smear microscopy can accurately detect TB in 20% to 80% (using fluorescence microscopy methods) of TB cases. However, it could be used to diagnose TB when sputum has sufficient bacillary load, and it cannot detect drug resistance. Thus, HIV-associated TB often goes undetected because people living with HIV (PLHIV), especially those with severe immunosuppression generally have very low numbers of bacilli Despite this range difference, it could be used to detect TB when the clinical sample contains sufficient tuberculosis micro-organism without testing of its resistant pattern. This leads to missing of the causative agent detection in immunocopromised individuals like those who are living with HIV and children since they could not produce enough sputum which has low numbers of bacilli [9].

[9]. Hence, GeneXpert MTB/RIF *X-Pert* assay test is sensitive and specific and an initial diagnostic test for TB and rifampicin resistance detection in patients suspected of having TB, MDR-TB or HIV-associated TB.used as an initial diagnostic test for TB detection and rifampicin resistance detection in patients suspected of having TB, MDR-TB, or HIV-associated TB is sensitive and specific [10]. Therefore, the goal aim of this study was to determine magnitude of *Mycobacterium tuberculosis*, its drug resistance and its associated factors among TB presumptive TB patients referred to St. Paul's Hospital Millennium Medical College, Addis Ababa, Ethiopia.

Formatted: Font: Not Italic

Formatted: Font: Not Italic

Materials and methods

Study area

Formatted: Highlight

The study was conducted in St Paul's Hospital Millennium Medical College, Addis Ababa, Ethiopia. The hospital receives referrals from around the country and is under the guidance of the Ethiopian Federal Ministry of Health. It is currently has 392 beds, with an annual average of 200,000 patients and a catchment population of more than 5 million. The microbiology laboratory receives and performs in average 5 sputum and 2 extra-pulmonary clinical samples per day. ~~The hospital receives referrals from around the country and is under the guidance of the Ethiopian Federal Ministry of Health.~~

Study design and period

A cross-sectional study was conducted at SPHMMC, Addis Ababa, Ethiopia from January to July 2019, and all patients who visited were source population while all ~~*Mycobacterium*~~ pulmonary~~tuberculosis~~ presumptive tuberculosis patients visited microbiology laboratory and fulfill the inclusion criteria were considered as study population.

Inclusion and Exclusion criteria

All presumptive *Mycobacterium tuberculosis* patients visiting Microbiology laboratory were included and patients who have inadequate specimen, previous history of known ~~multi~~drug resistance for *Mycobacterium tuberculosis* were excluded from the study.

Variables

Magnitude of *Mycobacterium tuberculosis* and its drug resistance pattern among presumptive patients dependent variables. Whereas, socio-demographic characteristics (age, sex, ~~etc~~etc.), possible risk factors like; TB contact history, previous treatment for TB, presumptive Drug Resistance Tuberculosis (DRTB), BCG vaccination status, CD4^{+} and HIV viral load counts were independent variables.

Formatted: Font: Not Italic

Formatted: Font: 14 pt, Superscript

Sample size and sampling technique

The sample size was estimated based on the assumption of single population proportion formula, considering the proportion 50%, 5% marginal error, and 95% confidence level to get the highest sample size, the calculation result determined as:

$$n = \frac{(z^{\alpha}/_{2})^2 \, p\,(1-p)}{d^2} \Longrightarrow \frac{(1.96)^2 \, 0.5(1-0.5)}{(0.05)^2} = 384 \text{ study subjects.}$$

Where: n = minimum sample size,

P = estimated proportion of *Mycobacterium tuberculosis* for the study population, and taking 10% non-response rate, the final sample size become 422 participants.

d= the margin of sample error, $z^{\alpha}/_{2}$= the standard normal variable at $1-^{\alpha}/_{2}$ confidence level and we used consecutive sampling technique was used to select the study population.

Data collection procedure

Data collectors were trained and informed how to collect the data. Structured questionnaire was used to collect the socio-demographic status and associated risk factors of the study participants. From each presumptive *Mycobacterium tuberculosis* patients, 2-4 ml of clinical sputum sample pus, lymph node aspirate or peritoneal and pleural fluid, and gastric aspirate samples wereas collected. We used Gastric aspirate or induced sputum by physician support for children unable to provide sputum by coughing.

Laboratory procedures

Microscopy and GeneXpert MTB/RIF gene X-pert® MTB/RIF, microscopy and culture tests were done parallel for all samples.

In the case of sputum samples, pellets were used for GeneXpert MTB/RIF assay. Sample reagent (1.5 ml) was added to 0.5 ml of the re-suspended sputum pellet and manually agitated twice at room temperature during a 15-minutes period.

In the case of other clinical body fluids, we transferred the entire specimen to a conical centrifuge tube, and concentrate the specimen at 3000 g for 15 minutes , carefully poured off the supernatant through a funnel into a discard can containing 5% sodium hypochlorite, re-suspend the deposit to a final volume of 2 ml by Phosphate Buffer Saline (PBS) , using transfer pipette, we added a double volume of the GeneXpert MTB/RIF sample Reagent (1.4 ml) to 0.7 ml (2:1) of suspension, then 2 ml transferred to the concentrate and load to the GeneXpert MTB/RIF cartridge.

The GeneXpert MTB/RIF ~~Gene X-pert® MTB/RIF~~ purifies ~~and concentrates~~ *M. tuberculosis* ~~bacilli~~ from these clinical samples. The genetic content of *M. tuberculosis* –captured ~~Genomic material isolated from the captured bacteria by sonication~~ and subsequently amplifies the genomic DNA by polymerase chain reaction (PCR). In addition, it identifies RIF's resistance ~~Furthermore, the process identifies all the clinically relevant rifampicin resistance inducing~~ mutations in the RNA polymerase beta (rpoB) gene ~~in the~~of *M. tuberculosis* in all clinically important samples within 2 hours.- ~~genome in a real-time format using fluorescent probes Gene X-pert is capable of detecting rifampicin resistance in pulmonary and extra-pulmonary specimens from clinical cases of TB. The Gene X-pert can detect mutations in the rpoB gene and show the results in <2 hours, finally the results were recorded~~ [11].

Regarding microscopic examination, all sputum smears are prepared from decontaminated and concentrated specimens. The smears stained with Ziehl-Neelsen (ZN) staining techniques, could be used to count both viable and non-viable bacilli as acid-fast bacilli (AFB).

Formatted: Adjust space between Latin and Asian text, Adjust space between Asian text and numbers

Formatted: Font: Italic

Formatted: Font: Not Italic

Formatted: Font: Not Italic

This method uses a carbolfuchsin as primary stain, acid alcohol as decolorizer, and methylene blue as counterstain. Acid-fast organisms stain red, while the background of debris stains blue. The ZN stain confirms the acid-fast property of mycobacteria using microscopy examination. Bacillary density will be graded as scanty, 1+, 2+, and 3+, and all such smears will be defined as ‘‘smear-positive’’.

Lowenstein-Jensen (LJ) culture medium was used which incorporates congo red and malachite green to inhibit unwanted bacteria for culturing. Once good growth was obtained, the positive slants were stored in a cool, dark place to archive the positive *M. tuberculosis* isolates.

Data Quality Assurance

The questionnaire was pre-tested and proper training prior to the actual data collection was given for data collectors. The necessary adjustments were made after the pre-test. The quality of data was maintained through strictly following the pre-analytical, analytical and post-analytical steps through each day supervision and SOPs of the laboratory procedures.

Data analysis and interpretation

The collected data were entered to EPI info 2002 version 3.32 after data editing and cleaning it was exported to SPSS version 23 windows software computer program for analysis. The logistic regression was employed to assess the association between TB and risk of different factors. A p-value of less than 0.05 was considered as statistical significance.

Ethical considerations

This study was approved by Department of Medical Laboratory Sciences, College of Health Sciences, Addis Ababa University, Addis Ababa, Ethiopia. giving a reference number SR/LS/025/19. Then official permission was obtained from Institutional Review Board (IRB) of St. Paul’s Hospital Millennium Medical College, Addis Ababa, Ethiopia, and then submitted to

laboratory department. Written informed consent was secured from each participant greater than 18 years old and assents were obtained for those less than 18 years old.. Patients' names were not recorded on the questionnaire to guarantee confidentiality of the information and privacy of the patients. Infected patients and/or those who had resistance *M. tuberculosis* were informed to their health care provider for better care and management.

Operational Definition

MDR-TB: is non-susceptibility of *Mycobacterium Tuberculosis* at least two first line TB drugs (isoniazid and rifampicin).

Presumptive TB: a patient who presents with symptoms or signs suggestive of TB including cough >2week, fever >2week, significant weight loss, haemoptysis, any abnormality chest radiograph.

Formatted: Line spacing: 1.5 lines

Presumptive MDR-TB: smear positive previously treated patients who define as relapse, return after default, and failure; new smear positive pulmonary TB patients who sputum remains smear positive at month 2 or 3 of treatment.

Results

Socio-demographic characteristics

The total of 436 participants were enrolled during the study periodrespondents were included in the study, of this 223 (51%) were male. The mean ± SD age the participants were 38±17years. The highest age range category was 35-49 years and the least was less than 15 years old. Majority of the respondents were 240 (55%) were urban resident, 214 (49%) had monthly income 100-1000 Ethiopian Birr ,(tTable 1).

Clinical data

There were From the total 374 (85.8%) people with were suspected for pulmonary tuberculosis and 62 (14.2%) people with were suspected for extra-pulmonary tuberculosis in total, with 130 (30–%) of them werebeing HIV positive positiveindividuals. About 422 (96.8%) of the participants were presumptive TB and whereas presumptive DRTB was found 14(3.2%) of the total participants.were presumptive DRTB. In this study 33(7.5%) had history of TB patient/family contact, 68(15.6%) history of alcohol drinking, 22 (5%) history of cigarettes smoking, 319 (73.1%) fever, 311 (71.3%) night sweating and 365(83.7%) had cough. Out of 130 HIV positive participants, 104 (81%) were on anti-HIV treatment and monitored their $CD4^+$ T-cells count, in addition, 119 (91.5%) participants tested for HIV viral load. Higher magnitude seems to be appeared for those who have $CD4^+$count 200-350/mm^3 (5/34) and their viral load was ≥1000/mm^3 (6/90)) , (tTable 2).

Magnitude of *M. tuberculosis* and resistance pattern

Out of the total participants, 36(8.3%) were detected with GeneXpert MTB/RIFX pert, and of this figure which only 2 (0.5 %) of them were RIF resistant. Regarding culture result, 27(6.2%) was positive and one *M. tuberculosis* strain was resistant for Isozianide drug (mono-resistant) and 2 were resistant for Isozianide and RIF (Multidrug resistant TB). The bivariate logistic regression analysis of socio-demographic characteristics showed, age of less than 15 years old has 1.8 times (95% CI: 0.4, 8.1) more likely to develop *M. tuberculosis* as compared to age greater than 50 years old, and widowed participants were 2.6 times (95% CI: 0.4, 17) more likely to have *M. tuberculosis* than single marital status, and Gov't Workers were 1.8 times (95% CI:

~~0.6, 5.9) more likely to have *M. tuberculosis* than housewife, however, none of the socio-demographic characteristics significantly associated with M. tuberculosis, table 3.~~

~~Bivariate analysis~~ Factors associated to *M. tuberculosis*

Formatted: Font: Bold

The bivariate logistic regression analysis of socio-demographic characteristics showed, age of less than 15 years old has 1.8 times (95% CI: 0.4, 8.1) more likely to develop *M. tuberculosis* as compared to age greater than 50 years old, and widowed participants were 2.6 times (95% CI: 0.4, 17) more likely to have *M. tuberculosis* than single marital status, and ~~Gov~~government'~~t~~ w~~W~~orkers were 1.8 times (95% CI: 0.6, 5.9) more likely to have *M. tuberculosis* than housewife, however, none of the socio-demographic characteristics significantly associated with *M. tuberculosis* (~~, t~~Table 3).

Formatted: Font: Italic

Presence of contact history with tuberculosis infected patients, pneumonia confirmed with chest-X-ray examination, and $CD4^{+}$results were associated factors for *M. Tuberculosis* in the bivariate logistic analysis, however, none of the factors associated in multivariable analysis (~~, t~~Table 4)~~.~~

Discussion

The highest TB frequency was observed in age groups of 35-49 years old, living in 4-6 family size / house, regarding to occupation; laborer workers, having monthly income 1001-2000 Ethiopian Birr. The TB magnitude among this productive age group (35-49) years of study participants was (9 [33.3%]). This might be due to more exposure to the high workload, and wide range of mobility in these age-groups.

~~In this study it seems that as the number of family size per house hold increase the prevalence of smear positivity also increases. Family size 5-6 was highly affected by *Mycobacterium tuberculosis*. Different studies indicated individuals living in larger family size members and~~

Formatted: noindent, Font Alignment: Baseline

malnutrition are at higher risk of developing pulmonary tuberculosis The present study showed as the number of individuals living together increase (5-6 family size), the positivity of *Mycobacterium tuberculosis* also to be increased. It is documented in other literature also that higher number family size living together and malnutrition contribute for the development of the disease tuberculosis [12], however the current our study revealed that no association family size/house hold and *Mycobacterium tuberculosis*.

Higher *Mycobacterium tuberculosis* was detected from participants diagnosed the reason for presumptive tuberculosis 25/436 (5.7%), from non-vaccinated for BCG 18/436 (4.1%), in non-alcoholic drinkers 21/436 (4.8%), and non-cigarette smokers 25/436 (5.7%).

Again higher *Mycobacterium tuberculosis* result observed in tuberculosis symptoms like in those who have night sweating 23/436(5.2%), fever 22/436(5.0%), weight loss 20/436(4.5%), cough 24/436(5.5%), loss of appetite 20/436(4.5%), and chest pain 16/436(3.7%). The least results were observed those who have diarrhea 3/436(0.7%), dyspnea 9/436 (2.0%), and external-adenopathy 3/436 (0.7%) sign and symptoms of *Mycobacterium tuberculosis.*

Despite the disease of tuberculosis measured by person contact with the diseased person who have already developed cough, smoking and alcoholic drinking habit, in our study it is presented that not significantly higher for individuals having contact history, drinking alcohol, smoking habit and previous history of anti-TB treatment.

The distribution of pulmonary tuberculosis was also measured in terms of contact history with chronic coughers, smoking habit and alcoholism to trace the epidemiological features of the disease. In this study, the magnitude of pulmonary tuberculosis was not significantly high in those who had contact with TB infected patients, previous history of anti-TB treatment, drinking

~~of alcohol and in those who were smokers.~~ These findings were different from the studies done in Addis Ababa, Ethiopia in 2011 and north Gondar in 2015 [13, 14].

The possible reason might be due to lower number of participant diagnosis the reason for presumptive DRTB in our cases and using more number of participants from urban resident.

Higher result again observed in previously treated patients 20/436 (4.5%) with anti-TB drugs and in new patients for presumptive drug resistance tuberculosis 24/436 (5.5%).

Statistically significant association was observed between culture positive pulmonary tuberculosis and TB contact History and some of tuberculosis patient symptoms weight loss, having pneumonia and CD 4^+ counts. The previous study also indicated that pulmonary tuberculosis associated with the level of $CD4^+$ in HIV patients and the amount of virus present in the participant's blood [13, 15].

The current result seems similar with reports of 10% (20 individuals smear positive) study conducted in Addis Ababa, Ethiopia in 2017 [16], prisons settings of East Gojjam Zone, Northwest Ethiopia using GeneXpert MTB/RIF~~Gene *X-pert* MTB/RIF~~, 9(3.4%) [17] and 9.9 % of the study conducted in extra pulmonary tuberculosis at University of Gondar, Northwest Ethiopia [18]. This overall culture confirmed *Mycobacterium Tuberculosis,* 27/436(6.2%) magnitude is lower than the study conducted in the Health Centers of Addis Ababa, Ethiopia reported as 46.0% (233/506) [13], from Metehara sugar factory hospital, eastern Ethiopia (14.2%)~~[18]~~ and 124 (32.2 %) of studied in two public hospitals in East Gojjam zone, northwest Ethiopia [19].

As compared to retrospective study report, from the University of Gondar Hospital from January 2013 to August 2015, prevalence of (24.6%), we found low result [20].

Our finding also lower than 23.2% of the study conducted in Debre markos Referral Hospital, Ethiopia using GeneXpert MTB/RIF Gene *X-pert* MTB/RIF assay.

The possible reason for the difference might be associated with the variation of the diagnostic methods we used, for example in our cases we used sputum sedimentation concentration technique for microscopic smear examination, GeneXpert MTB/RIF Gene *X-pert* assay and finally LJ culture for confirmation whereas, a single diagnostic tool used in the previous study like; stained by Ziehl-Neelsen staining and examined by Microscopy in the case of Metehara [18], using GeneXpert MTB/RIF Gene *X-pert* MTB/RIF in the case of prisons settings of East Gojjam Zone [17]. This low prevalence may also mean that there might be a comparatively good TB infection control around our study area, Addis Ababa, Ethiopia.

From the overall confirmed *Mycobacterium Tuberculosis* 6.2% (27/436), a total of three *Mycobacterium tuberculosis* strain showed resistance pattern to anti-tuberculosis drug, of which two of them were multi drug (INH and RIF) resistance strains. This result lower than the study conducted in the University of Gondar Hospital, northwest Ethiopia which is resulted as 71(15%) resistant to rifampicin [20] among tuberculosis-presumptive cases at University of Gondar Hospital, northwest Ethiopia, 15.58 % of two public hospitals in East Gojjam zone, northwest Ethiopia [19],[19] and 12 (10.3%) patients referred to Debre markos Referral Hospital, Ethiopia [21].

From a total of 130 HIV positive status, MTB was detected in only 10 (7.7%) of the participants. Out of this sero-positive figure, one mono (INH) resistant and one MDR-–TB (INH+RIF) resistant strains were detected. Regarding participants' viral load and TB relation, only one mono resistant strain was found in the participant serum which contains high copies of viral load count ($\geq$1000/mmm^3). This might be due to HIV infection; HIV infection may cause mal-absorption of

anti-TB drugs and immune suppression which leads to resistance and our result is supported by other findings [22, 23].

The bivariate logistic analysis showed that presumptive drug resistance tuberculosis two times more likely (2.6 (95% CI 0.6, 12, p=0.2)) to develop tuberculosis than presumptive tuberculosis; also having the symptoms of night sweating two times more likely (2.4(95% CI 0.8, 7.2, p=0.1)) to develop tuberculosis than those who did not the symptoms of night sweating. Having the presence of chest pain also (1.6 (95% CI 0.8, 3.7, p=0.2)) times more likely to develop *Mycobacterium tuberculosis* than from those who did not have chest pain.

Conclusion

In general, this study found low-magnitude *Mycobacterium tuberculosis* in presumptive patients at SPHMMC, Addis Ababa, Ethiopia, and three DRTB strains, including two MDR strains were observed on those who have history of failure, relapse and previously treated with anti-TB treatment.

Presence of contact history with previous tuberculosis infected patients, patient weight loss, presence of pneumonia with radiological examination, and $CD4^+$ results were the identified symptoms and factors associated from *M. Tuberculosis* in the bivariate logistic analysis.

In general, this study highlights low magnitude *Mycobacterium tuberculosis* among presumptive patients visited to SPHMMC, Addis Ababa, Ethiopia, however from the total of three strains, two of MDR strains were observed on those who have history of failure, relapse and previously treated with anti-TB treatment.

Health education about tuberculosis, TB control programs should be continued and large community based study also recommended to sustain this low result of the disease.

Strengthening TB infection control activities and proper implementation of DOTS are also recommended to reduce the burden of MDR-TB.

Abbreviation

ATCC=American Type Culture Collection, BCG=Bacillus Calmette–Guérin, DST= Drug Susceptibility Test, FMOH=Federal Ministry of Health, HIV=Human Immunodeficiency Virus, INH=Isoniazid, IQC=Internal Quality Controls, ~~LPA=line probe assay~~, MDR-TB=Multidrug-Resistant Tuberculosis, MOTT= Mycobacteria other than TB, MTB= *Mycobacterium Tuberculosis*, OADC = Oleic Acid Albumin Dextrose Complex, PPE=Personal protective equipment, PTB= Pulmonary Tuberculosis, RIF=Rifampicin, RMR= Rifampicin Mono-Resistant, SOP= Standard Operating Procedures, SPHMMC= Saint Paul's Hospital Millennium Medical College, SR=sample reagent, STM=Streptomycin, TB=Tuberculosis, TTD= Time to detection, WHO= World Health Organization~~, XDR-TB= Extensively Drug-Resistant Tuberculosis~~.

Formatted: Font: Italic

Declaration

~~**Ethics approval and consent to participate**~~

~~The proposed study was approved by the Department of Medical Laboratory Science, Addis Ababa university research and ethics committee concerning the ethical issues giving a reference number SR/LS/025/19.~~

Consent for publication

Not applicable. This study does not contain any individual or personal data.

Availability of data and materials

All data relevant to this study are available on the manuscript.

Funding

This research work was supported by Addis Ababa University, Ethiopia. The funder had no role in study design, data collection and analysis, decision to publish, or preparation of the manuscript.

Authors' contributions

MK, KD, RA, ZY, AY, NZ, MA, BZ, MG and AG were involved in study conception, data collection and analysis, drafting the manuscript. MK, KD and AG reviewed critically the manuscript for intellectual content. All authors have read, edited and approved the manuscript.

Acknowledgement

Authors would like to thank the Departments of Medical Laboratory Science, College of Health Sciences, Addis Ababa University, Department of national tuberculosis reference laboratory staffs, members of Ethiopian Public Health Institute who were willing to use laboratory space and consumable fund, and also St. Paul's Hospital Millennium Medical College (SPHMMC) management, last but not leaste we are grateful for all study participants.

Formatted: Font: Not Italic

Competing interests

The authors declare that they have no competing interests.

References

1. Gelaw SM. Socioeconomic factors associated with knowledge on tuberculosis among adults in Ethiopia. Tuberc Res Treat. 2016; 2016:6207457. doi: 10.1155/2016/6207457. Epub 2016 Feb 1. PMID: 26949546; PMCID: PMC4753341Gelaw SM. Socioeconomic Factors Associated with Knowledge on Tuberculosis among Adults in Ethiopia [Internet]. Tuberculosis Research and Treatment. 2016 [cited 2019]. Available from: https://www.hindawi.com/journals/trt/2016/6207457/

Formatted: Font: Not Highlight

2. World Health Organization. Global tuberculosis report 2021. https://www.who.int/publications/i/item/9789240037021 (Accessed on November 08, 2021).Churchyard GJ, Swindells S. Controlling latent TB tuberculosis infection in high-burden countries: A neglected strategy to end TB. PLOS Med. 2019 ;16(4):e1002787.

3. Global Tuberculosis Report 2019. Geneva: World Health Organization; 2019. https://www.who.int/publications/i/item/9789241565714Organization WH. Global tuberculosis report 2015. 2015. Geneva World Health Organ. 2015;

4. Girum T, Muktar E, Lentiro K ,Wondiye H and Shewangizaw M. Epidemiology of multidrug-resistant tuberculosis (MDR-TB) in Ethiopia: a systematic review and meta-analysis of the prevalence, determinants and treatment outcome. Tropical Diseases, Travel Medicine and Vaccines. 2018 Jun 14;4:5. doi: 10.1186/s40794-018-0065-5. PMID: 29942536; PMCID: PMC6000958. Jaleta KN, Gizachew M, Gelaw B, Tesfa H, Getaneh A, Biadgo B. Rifampicin-resistant Mycobacterium tuberculosis among tuberculosis-presumptive cases at University of Gondar Hospital, northwest Ethiopia. Infect Drug Resist. 2017 ;10:185–92.

5. Asgedom SW, Tesfaye D, Nirayo YL, Atey TM. Time to death and risk factors among tuberculosis patients in Northern Ethiopia. BMC Res Notes. 2018;11(1):696. doi: 10.1186/s13104-018-3806-7. PMID: 30286801; PMCID: PMC6172746.

6. Atalell KA, Tebeje NB, Ekubagewargies DT. Survival and predictors of mortality among children co-infected with tuberculosis and human immunodeficiency virus at University of Gondar Comprehensive Specialized Hospital, Northwest Ethiopia. A retrospective follow-up study. PLoS One. 2018 May 22;13(5):e0197145. doi:

Formatted: Font: (Default) Times New Roman, 12 pt

Formatted: Font: (Default) Times New Roman, 12 pt

Formatted: Font: (Default) Times New Roman, 12 pt

Formatted: Font: (Default) Times New Roman, 12 pt

10.1371/journal.pone.0197145. PMID: 29787596; PMCID: PMC5963769.PLOS ONE. 2018 ;13(5):e0197145.

7. Woya AA, Tekile AK, Basha GW. Spatial Frailty Survival Model for Multidrug-Resistant Tuberculosis Mortality in Amhara Region, Ethiopia [Internet]. Tuberculosis Research and Treatment. 2019 [cited 2019];1(1) Woya AA, Tekile AK, Basha GW. Spatial Frailty Survival Model for Multidrug-Resistant Tuberculosis Mortality in Amhara Region, Ethiopia. Tuberc Res Treat. 2019 Jan 1;2019:8742363. doi: 10.1155/2019/8742363. PMID: 30693105; PMCID: PMC6332868.

8. Deribew A, Deribe K, Dejene T, Tessema GA, Melaku YA, Lakew Y, et al. Tuberculosis Burden in Ethiopia from 1990 to 2016: Evidence from the Global Burden of Diseases 2016 Study. Ethiop J Health Sci [Internet]. 2018 [cited 2019];28(5). Deribew A, Deribe K, Dejene T, Tessema GA, Melaku YA, Lakew Y, Amare AT, Bekele T, Abera SF, Dessalegn M, Kumsa A, Assefa Y, Kyu H, Glenn SD, Misganaw A, Biadgilign S. Tuberculosis Burden in Ethiopia from 1990 to 2016: Evidence from the Global Burden of Diseases 2016 Study. Ethiop J Health Sci. 2018 Sep;28(5):519-528. doi: 10.4314/ejhs.v28i5.2. PMID: 30607066; PMCID: PMC6308773.

9. Piatek AS, Cleeff MV, Alexander H, Coggin WL, Rehr M, Kampen SV, et al. GeneXpert for TB diagnosis: planned and purposeful implementation. Glob Health Sci Pract. 2013;1(1):18–23. doi: 10.9745/GHSP-D-12-00004. PMID: 25276513; PMCID: PMC4168558.

10. Steingart KR, Sohn H, Schiller I, Kloda LA, Boehme CC, Pai M, et al. Xpert® MTB/RIF assay for pulmonary tuberculosis and rifampicin resistance in adults. Cochrane Database

Formatted: Not Highlight

Formatted: Highlight

Formatted: Font: (Default) +Body (Calibri), 11 pt, Highlight

Syst Rev. 2013 Jan 31;(1):CD009593. doi: 10.1002/14651858.CD009593.pub2. Update in: Cochrane Database Syst Rev. 2014;(1):CD009593. PMID: 23440842; PMCID: PMC4470352. 2013;1(1).

11. Atashi S, Izadi B, Jalilian S, Madani SH, Farahani A, Mohajeri P. Evaluation of GeneXpert MTB/RIF for determination of rifampicin resistance among new tuberculosis cases in west and northwest Iran. New Microbes New Infect. 2017 ;19:117–20. doi: 10.1016/j.nmni.2017.07.002. PMID: 28794886; PMCID: PMC5547232.

12. Zenebe Y, Anagaw B, Tesfay W, Debebe T, Gelaw B. Smear positive extra pulmonary tuberculosis disease at University of Gondar Hospital, Northwest Ethiopia. BMC Res Notes. 2013 ;6(1):21. doi: 10.1186/1756-0500-6-21. PMID: 23331864; PMCID: PMC3558382.

13. Deribew A, Negussu N, Melaku Z, Deribe K. Investigation Outcomes of Tuberculosis Suspects in the Health Centers of Addis Ababa, Ethiopia. PLOS ONE. 2011 ;6(4):e18614. doi: 10.1371/journal.pone.0018614. PMID: 21526179; PMCID: PMC3079716.

14. Mekonnen F, Tessema B, Moges F, Gelaw A, Eshetie S, Kumera G. Multidrug resistant tuberculosis: prevalence and risk factors in districts of metema and west armachiho, Northwest Ethiopia. BMC Infect Dis. 2015 ;15(1):461. doi: 10.1186/s12879-015-1202-7. PMID: 26503269; PMCID: PMC4624367.

15. Lawn SD, Badri M, Wood R. Tuberculosis among HIV-infected patients receiving HAART: long term incidence and risk factors in a South African cohort. AIDS. 2005 ;19(18):2109 -16. doi: 10.1097/01.aids.0000194808.20035.c1. PMID: 16284460.

16. Nugussie DA, Mohammed GA, Tefera AT. Prevalence of Smear-Positive Tuberculosis among Patients Who Visited Saint Paul's Specialized Hospital in Addis Ababa, Ethiopia [Internet]. BioMed Research International. Nugussie DA, Mohammed GA, Tefera AT. Prevalence of Smear-Positive Tuberculosis among Patients Who Visited Saint Paul's Specialized Hospital in Addis Ababa, Ethiopia. Biomed Res Int. 2017;2017:6325484. doi: 10.1155/2017/6325484. Epub 2017 Aug 21. PMID: 28904965; PMCID: PMC5585560.2017 [cited 2019 Oct 7].

Formatted: Highlight

Formatted: Highlight

17. Gizachew Beza M, Hunegnaw E, Tiruneh M. Prevalence and associated factors of tuberculosis in prisons settings of East Gojjam Zone, Northwest Ethiopia. Int J Bacteriol. 2017;2017 :3826980. doi: 10.1155/2017/3826980. Epub 2017 Oct 17. Erratum in: Int J Bacteriol. 2018 Aug 9;2018:1020349. PMID: 29226216; PMCID: PMC5684609.

18. 63. Yohanes A, Abera S, Ali S. Smear positive pulmonary tuberculosis among suspected patients attending metehara sugar factory hospital; eastern Ethiopia. Afr Health Sci. 2012 ;12(3):325-330 330. 325-30. doi: 10.4314/ahs.v12i3.12. PMID: 23382747; PMCID: PMC3557681.

Formatted: Highlight

Formatted: Highlight

19. Adane K, Ameni G, Bekele S, Abebe M, Aseffa A. Prevalence and drug resistance profile of Mycobacterium tuberculosis isolated from pulmonary tuberculosis patients attending two public hospitals in East Gojjam zone, northwest Ethiopia. BMC Public

Health. 2015 ;15(1):572. doi: 10.1186/s12889-015-1933-9. PMID: 26092570; PMCID: PMC4473837.

20. Jaleta KN, Gizachew M, Gelaw B, Tesfa H, Getaneh A, Biadgo B. Rifampicin-resistant Mycobacterium tuberculosis among tuberculosis-presumptive cases at University of Gondar Hospital, northwest Ethiopia. Infect Drug Resist. 2017 ;10:185–92. doi: 10.2147/IDR.S135935. PMID: 28652786; PMCID: PMC5476602.

21. Mulu W, Abera B, Yimer M, Hailu T, Ayele H, Abate D. Rifampicin-resistance pattern of Mycobacterium tuberculosis and associated factors among presumptive tuberculosis patients referred to Debre Markos Referral Hospital, Ethiopia: a cross-sectional study. BMC Res Notes. 2017 ;10(1):8. doi: 10.1186/s13104-016-2328-4. PMID: 28057041; PMCID: PMC5217448.

22. Wells CD, Cegielski JP, Nelson LJ, Laserson KF, Holtz TH, Finlay A, et al. HIV Infection and Multidrug-Resistant Tuberculosis—The Perfect Storm. J Infect Dis. 2007 ;196(Supplement_1):S86–107. doi: 10.1086/518665. PMID: 17624830.

23. Getahun H, Gunneberg C, Granich R, Nunn P. HIV Infection—Associated Tuberculosis: The Epidemiology and the Response. Clin Infect Dis. 2010 ;50(Supplement_3):S201–7. doi: 10.1086/651492. PMID: 20397949.

List of tables

Table 1: Socio-demographic characteristics and magnitude of *M. tuberculosis* drug resistance pattern and its associated factors among presumptive TB patients referred to at SPHMMC, Addis Ababa, Ethiopia, 2019.

Formatted: Font: Italic

Formatted Table

**Variables/ characteristics**		**No. of Participants**	**Percentages (%)**
**Sex**	Male	224	51.3
	Female	212	498.6
**Age groups**	<15 years	39	8.9
	15-24 years	56	132.8
	25-34 years	98	22.5
	35-49 years	127	29.1
	>50 yearsyrs	116	276.6
**Residence**	Urban	240	55.0
	Rural	196	45.0
**Family size/house**	1-3	152	34.95
	4-6	220	50.5
	>6	64	154.6
**Marital status**	Single	146	33.5
	married	238	554.6
	Divorced	20	4.65
	Widowed	32	7.3
**Occupational status**	Laborer	97	22.2
	Gov’tGovernment wWorkers	97	22.2
	Private workers	109	25.0

	House wife	70	16
	Student	63	15~~4.4~~
	No formal Education~~Illiterate~~	119	27~~.3~~
**Educational status**	1-8th grades	147	3~~3.7~~4
	9-12th grades	106	24~~.3~~
	>12th grade	64	1~~4.7~~5
	<100 Birr	60	14~~3.8~~
	100-1000 Birr	83	19~~.0~~
	1001-2000 Birr	155	35~~.6~~
**Monthly Income**	2001-3000 Birr	59	14~~3.5~~
	3001-4000 Birr	32	7~~.3~~
	4001-5000 Birr	25	6~~5.7~~
	>5001 Birr	22	5.0

Table 2: Clinical characteristics ~~for~~participants ~~magnitude of *M. tuberculosis, drug* resistance pattern and its associated factors among~~ among presumptive TB patients ~~referred to~~at SPHMMC, Addis Ababa, Ethiopia, 2019.

Formatted: Font: Italic

Formatted Table

**Variables/ Characteristics**		**Number of participants**	**Percentages (%)**
	Presumptive TB	422	9~~6.8~~7
Reason for d~~D~~iagnosis	Presumptive DRTB	14	3~~.2~~
	Vaccinated	156	36~~5.8~~
BCG v~~V~~accination	Non-Vaccinated	280	64~~.2~~
	Yes	33	8~~7.5~~

TB contact hHistory	No	403	92.5
	Yes	68	165.6
Alcohol dDrinking	No	368	84.4
	Smokers	22	5.1
Cigarette sSmoking	Non-smokers	414	95.0
	Yes	310	71.1
Night sSweating	No	126	28.9
	Yes	318	73.0
Presence of Ffever	No	118	27.0
	Yes	200	46.0
Weight loss	No	236	54.0
	Yes	364	83.5
Presence of Cough	No	72	176.5
	Yes	285	65.4
Loss of aAppetite	No	151	354.6
	Yes	206	47.2
Presence of cChest pPain	No	230	532.8
	Yes	57	13.0
Presence of dDiarrhea	No	379	87.0
	Yes	140	32.1
Presence of dyspnea	No	296	687.9
	Yes	63	14.4

External-~~A~~adenopathy	No	373	8~~5.~~6
Anti-TB Treatment	Previously treated	110	25~~.2~~
	Previously untreated	326	75~~4.8~~
Presumptive DRTB	New	384	88~~.1~~
	Relapse	46	11~~0.6~~
	Failure	6	1~~.4~~
HIV s~~S~~tatus	Positive	130	30~~29.8~~
	Negative	306	70~~.2~~
Tuberculosis type	PTB	373	86~~5.6~~
	EPTB	63	14~~.4~~
CD4+ c~~C~~ount	<200 cells/mm^3	16	16~~5.5~~
	200-350/mm^3	34	33~~.0~~
	>350/mm^3	53	51~~.5~~
HIV v~~V~~iral load	<1000/ mm^3	29	24~~.4~~
	≥1000/ mm^3	90	7~~5.~~6

BCG= Bacillus Calmette–Guérin, DRTB= Drug Resistance Tuberculosis, EPTB=Extra Pulmonary Tuberculosis HIV=Human Immunodeficiency Virus, MDR-TB=Multidrug-Resistant Tuberculosis, MTB= Mycobacterium Tuberculosis, PTB=Pulmonary Tuberculosis.

Table 3: ~~Bivariate analysis for~~ Socio-demographic factor analysis of *M. Tuberculosis* ~~socio-demographic factors~~ among presumptive TB patients ~~referred to~~at SPHMMC, Addis Ababa, Ethiopia, 2019.

Variables/ characteristics	#M.TB	#M.TB	~~#Total~~	COR (95%	P-

Formatted: Font: 10 pt, Italic
Formatted: Font: 10 pt, Italic
Formatted: Font: 10 pt, Italic
Formatted: Font: 10 pt, Italic
Formatted: Font: 10 pt, Italic
Formatted: Font: 9 pt, Italic
Formatted: Font: Italic

		Not detected (%) egative	Positive Detected (%)	Total (%)	CI)	value
**Sex**	Male	208 (93)	16 (7)	224 (51)	1.4(0.6-3.1)	0.4
	Female	201 (95)	11 (5)	212 (49)	1	
**Age groups**	<15 years	36 (92)	3 (8)	39 (9)	1.8(0.4, 8.1)	0.41
	15-24 years	52 (93)	4 (7)	56 (13)	1.7(0.4, 6.6)	0.44
	25-34 years	92 (94)	6 (6)	98 (22)	1.5(0.4, 4.9)	0.55
	35-49 years	118 (93)	9 (7)	127 (29)	1.7(0.6, 5.2)	0.36
	>50 years	111(96)	5 (4)	116 (27)	1	
**Residence**	Urban	227 (95)	13 (5)	240 (55)	1	
	Rural	182 (93)	14 (7)	196 (45)	1.4(0.6, 2.9)	0.4
**Family size/house**	1-3	144 (95)	8 (5)	152 (35)	1	
	4-6	204 (93)	16 (7)	220 (50)	1.4(0.6, 3.4)	0.4

	>6	61 (95)	3 (5)	64 (15)	0.9(0.3, 3.5)	0.8
**Marital status**	Single	136 (93)	10 (7)	146 (33)	1	
	Married	226 (95)	12 (5)	238 (55)	1(0.2, 5.3)	0.9
	Divorced	17 (85)	3 (5)	20 (5)	0.8(0.2, 3.7)	0.7
	Widowed	30 (94)	2(9)	32 (7)	2.6(0.4, 17)	0.3
**Occupational status**	Laborer	89 (92)	8 (8)	97 (22)	1.4(0.4, 4.6)	0.6
	Goverment’t Wworkers	91(94)	6 (6)	97 (22)	1.8(0.6, 5.9)	0.3
	Private workers	58 (92)	5 (8)	63 (15)	1.7(0.4, 6.4)	0.4
	Student	67 (96)	3 (4)	70 (16)	0.9(0.2, 4.0)	0.9
	House wife	104 (95)	5 (5)	109 (25)	1	
**Educational status**	Illiterate	112 (94)	7 (6)	119(72)	0.7(0.3, 2.4)	0.6
	1-8th grades	140 (95)	7 (5)	147(34)	0.6(0.2, 1.9)	0.4
	9-12th grades	98 (92)	8 (8)	106(24)	0.9(0.3, 3.0)	0.9
	>12th grade	59 (92)	5 (8)	64 (15)	1	
**Monthly Income**	<100 Birr	56 (93)	4 (7)	60 (14)	1.4(0.2, 13)	0.8
	100-1000 Birr	79 (95)	4 (5)	83 (19)	1(0.1, 9)	0.9
	1001-2000 Birr	147 (95)	8 (5)	155(36)	1(0.3, 9)	0.9
	2001-3000 Birr	54 (92)	5 (8)	59 (14)	1.8(0.2, 16)	0.6
	3001-4000 Birr	32 (100)	0 ()	32 (7)	-	0.9

	4001-5000 Birr	20 (80)	5 (20)	25 (6)	5(0.5, 46)	0.2
	>5001 Birr	20 (91)	2 (9)	22 (5)	1	

N.B: BCG= Bacillus Calmette–Guérin, COR= Crude Odds Ratio, DRTB= Drug Resistance Tuberculosis, HIV=Human Immunodeficiency Virus, MDR-TB=Multidrug-Resistant Tuberculosis, MTB= Mycobacterium Tuberculosis, TB=Tuberculosis

Table 4: ~~Bivariate analysis of clinical factors for~~Clinical factors associated with Magnitude ~~M~~of M. *tuberculosis* among presumptive TB patients ~~referred to~~at SPHMMC, Addis Ababa, Ethiopia, 2019.

**Variables/ characteristics**		**~~Result of~~ M.TB**			**COR (95%CI)**	**P-value**
		**#Not detected (%)~~egative~~**	**#Detected (%)~~Positive~~**	**#Total()**		
Reason for Diagnosis	Presumptive TB	397 (94)	25 (6)	422 (97)	1	
	Presumptive DRTB	12 (86)	2 (14)	14 (3)	2.6(0.6, 12)	0.2
BCG Vaccination	Vaccinated	147 (94)	9 (6)	156 (36)	1	
	Non-Vaccinated	262 (94)	18 (6)	280 (64)	1.1(0.5, 2.6)	0.7
TB contact History	Yes	28 (85)	5 (15)	33 (8)	3.1(1.1, 8.7)	0.03
	No	381 (95)	22 (5)	403 (92)	1	
Alcohol Drinking	Yes	62 (91)	6 (9)	68 (16)	1.6(0.6, 4.1)	0.3
	No	347(94)	21(6)	368 (84)	1	

Cigarette sSmoking	Smokers	20 (91)	2 (9)	22 (5)	1.6(0.3,7.0)	0.5
	Non-smokers	389 (94)	25 (6)	414 (95)	1	
Chest X-ray	Pneumonia	25 (89)	3 (11)	28 (7)	3(33, 319)	0.02
	Interstitial	28 (90)	3 (10)	31(7)	3(0.3, 30)	1.0
	Bronchiectasis	11(92)	1 (8)	12 (3)	2.6(0.3, 27)	0.34
	Bilateral	6 (43)	8 (57)	14 (3)	9(0.9, 8)	0.4
	Unilateral	14 (74)	5 (24)	19 (4)	0.5(0.6, 4.3)	0.5
	Normal	3234 (98)	7 (2)	331(76)	1	
Anti-TB tTreatment	Untreated	103 (94)	7(6)	110 (25)	1	
	Previously treated	306 (94)	20 (6)	326 (75)	1.1(0.4, 2.5)	0.9
Presumptive DRTB	New	362 (94)	24 (6)	386 (89)	1	
	Relapse	44 (91)	2 (9)	46 (11)	0.7(0.2, 3)	0.6
	Failure	3 (75)	1(25)	4 (1)	5.0(0.5, 5.0)	0.2
HIV sStatus	Positive	120 (92)	10 (8)	130 (30)	1.4(0.6, 3.1)	0.4
	Negative	289 (94)	17 (6)	306 (70)	1	
CD-4 cCount/ mm^3 blood	<200	16 (100)	0 ()	16 (15)	1.2(0.9, 2.4)	0.9
	200-350	29 (85)	5 (15)	34 (33)	8.9(0.5, 0.9)	0.049
	≥350	52 (96)	2 (4)	54 (52)	1	
Viral Load /mm^3 blood	<1000	27 (93)	2 (7)	29 (24)	1	
	≥1000	84 (93)	6 (7)	90 (76)	0.9(0.2, 5.0)	0.9

BCG= Bacillus Calmette–Guérin, CD= Cluster of Differentiation, Crude Odds Ratio, DRTB= Drug Resistance Tuberculosis, HIV=Human Immunodeficiency Virus, MDR-TB=Multidrug-Resistant Tuberculosis, MTB= Mycobacterium Tuberculosis, TB=Tuberculosis

View Letter

-

Date: Mar 16 2022 10:05AM
To: "Addisu Gize Yeshanew" konjoaddisu@gmail.com
From: "PLOS ONE" plosone@plos.org
Subject: PLOS ONE Decision: Revision required [PONE-D-22-03876]

Attachment(s): PONE-D-22-03876_ suggesions and comnts.pdf

PONE-D-22-03876
Magnitude of Multidrug Resistance Mycobacterium tuberculosis and associated factors among presumptive patients at St. Paul's Hospital Millennium Medical College, Addis Ababa, Ethiopia
PLOS ONE

Dear Dr. Yeshanew,

Thank you for submitting your manuscript to PLOS ONE. After careful consideration, we feel that it has merit but does not fully meet PLOS ONE's publication criteria as it currently stands. Therefore, we invite you to submit a revised version of the manuscript that addresses the points raised during the review process.

Please submit your revised manuscript. If you will need significantly more time to complete your revisions, please reply to this message or contact the journal office at plosone@plos.org. When you're ready to submit your revision, log on to https://www.editorialmanager.com/pone/ and select the 'Submissions Needing Revision' folder to locate your manuscript file.

Please include the following items when submitting your revised manuscript:

- A rebuttal letter that responds to each point raised by the academic editor and reviewer(s). You should upload this letter as a separate file labeled 'Response to Reviewers'.
- A marked-up copy of your manuscript that highlights changes made to the original version. You should upload this as a separate file labeled 'Revised Manuscript with Track Changes'.
- An unmarked version of your revised paper without tracked changes. You should upload this as a separate file labeled 'Manuscript'.

If you would like to make changes to your financial disclosure, please include your updated statement in your cover letter. Guidelines for resubmitting your figure files are available below the reviewer comments at the end of this letter.

If applicable, we recommend that you deposit your laboratory protocols in protocols.io to enhance the reproducibility of your results. Protocols.io assigns your protocol its own identifier (DOI) so that it can be cited independently in the future. For instructions see: https://journals.plos.org/plosone/s/submission-guidelines#loc-laboratory-protocols. Additionally, PLOS ONE offers an option for publishing peer-reviewed Lab Protocol articles, which describe protocols hosted on protocols.io. Read more information on sharing protocols at

https://plos.org/protocols?utm_medium=editorial-email&utm_source=authorletters&utm_campaign=protocols.

We look forward to receiving your revised manuscript.

Kind regards,

Frederick Quinn
Academic Editor
PLOS ONE

Response: Dear Frederick Quinn, Academic Editor, PLOS ONE

We authors would like to thank for your invitation to submit our revised version of the manuscript, PONE-D-22-03876 titled" Magnitude of Multidrug Resistance Mycobacterium tuberculosis and associated factors among presumptive patients at St. Paul's Hospital Millennium Medical College, Addis Ababa, Ethiopia", that addresses the points raised during the review process.

We included a point-by-point response within the 'Response to Reviewers' box in the submission system and modifications are highlighted in the track changes of the original manuscript, and we uploaded also the clean or unmarked version of our revised paper without tracked changes.

Journal Requirements:

When submitting your revision, we need you to address these additional requirements.

1. Please ensure that your manuscript meets PLOS ONE's style requirements, including those for file naming. The PLOS ONE style templates can be found at https://journals.plos.org/plosone/s/file?id=wjVg/PLOSOne_formatting_sample_main_body.pdf and https://journals.plos.org/plosone/s/file?id=ba62/PLOSOne_formatting_sample_title_authors_affiliations.pdf

Response: Thank you for the concern, the PLOS ONE's style requirement has been incorporated accordingly in all sections.

2. Thank you for submitting the above manuscript to PLOS ONE. During our internal evaluation of the manuscript, we found significant text overlap between your submission and the following previously published works, some of which you are an author.

- https://www.hindawi.com/journals/trt/2016/6207457/
- https://www.dovepress.com/rifampicin-resistant-mycobacterium-tuberculosis-among-tuberculosis-pre-peer-reviewed-fulltext-article-IDR
- https://www.ghspjournal.org/content/1/1/18.full
- https://www.ncbi.nlm.nih.gov/pmc/articles/PMC3557681/

We would like to make you aware that copying extracts from previous publications, especially outside the methods section, word-for-word is unacceptable. In addition, the reproduction of text from published reports has implications for the copyright that may apply to the publications.

Please revise the manuscript to rephrase the duplicated text, cite your sources, and provide details as to how the current manuscript advances on previous work. Please note that further consideration is dependent on the submission of a manuscript that addresses these concerns about the overlap in text with published work.

We will carefully review your manuscript upon resubmission, so please ensure that your revision is thorough.

Response: Thank you for your concern again and the constructive comment. We have rephrased and completely modified each word taken from the previous published articles.

3. Please include additional information regarding the survey or questionnaire used in the study and ensure that you have provided sufficient details that others could replicate the analyses. For instance, if you developed a questionnaire as part of this study and it is not under a copyright more restrictive than CC-BY, please include a copy, in both the original language and English, as Supporting Information.

Response: Regarding our survey tool or questionnaire, we have uploaded as supporting file and any interested can replicate for the study and analyses.

4. Thank you for stating the following in the Acknowledgments Section of your manuscript:

"Authors would like to thank the Departments of Medical Laboratory Science, College of Health Sciences, Addis Ababa University, Department of national tuberculosis reference laboratory staffs, members of Ethiopian Public Health Institute who were willing to use laboratory space and consumable fund, and also St. Paul’s Hospital Millennium Medical College (SPHMMC) management, last but not lease we are grateful for all study participants."

We note that you have provided funding information that is not currently declared in your Funding Statement. However, funding information should not appear in the Acknowledgments section or other areas of your manuscript. We will only publish funding information present in the Funding Statement section of the online submission form.
Please remove any funding-related text from the manuscript and let us know how you would like to update your Funding Statement. Currently, your Funding Statement reads as follows:

"This research work was supported by Addis Ababa University, Ethiopia.
The funders had no role in study design, data collection and analysis, decision to publish, or preparation of the manuscript."

Please include your amended statements within your cover letter; we will change the online submission form on your behalf.

Response: We included our Funding statement in the cover letter, same time the statement which stated about our funding information is removed from the revised manuscript.

5. Your ethics statement should only appear in the Methods section of your manuscript. If your ethics statement is written in any section besides the Methods, please move it to the Methods section and delete it from any other section. Please ensure that your ethics statement is included in your manuscript, as the ethics statement entered into the online submission form will not be published alongside your manuscript.

[Note: HTML markup is below. Please do not edit.]

Response: The comment is well taken and based on the comment; we have stated the ethics statement only in the method section of the revised manuscript.

Reviewers' comments:

Reviewer's Responses to Questions

Comments to the Author

1. Is the manuscript technically sound, and do the data support the conclusions?

The manuscript must describe a technically sound piece of scientific research with data that supports the conclusions. Experiments must have been conducted rigorously, with appropriate controls, replication, and sample sizes. The conclusions must be drawn appropriately based on the data presented.

Reviewer #1: Partly

Reviewer #2: Partly

Response: It is true that one's perception different from other. However, we author's believe that our manuscript technically sound, and do the data support the manuscript conclusions.

---

2. Has the statistical analysis been performed appropriately and rigorously?

Reviewer #1: No

Reviewer #2: I Don't Know

Response: Again it may be due to personal difference or may be because of reviewers are not public health professionals. However, we author's consulted by health professionals for our statistical analysis, and we believe that the statistical analysis has been performed appropriately.

---

3. Have the authors made all data underlying the findings in their manuscript fully available?

The PLOS Data policy requires authors to make all data underlying the findings described in their manuscript fully available without restriction, with rare exception (please refer to the Data Availability Statement in the manuscript PDF file). The data should be provided as part of the manuscript or its supporting information, or deposited to a public repository. For example, in addition to summary statistics, the data points behind means, medians and variance measures should be available. If there are restrictions on publicly sharing data—e.g. participant privacy or use of data from a third party—those must be specified.

Reviewer #1: Yes

Reviewer #2: Yes

Response: Thank you.

---

4. Is the manuscript presented in an intelligible fashion and written in standard English?

PLOS ONE does not copyedit accepted manuscripts, so the language in submitted articles must be clear, correct, and unambiguous. Any typographical or grammatical errors should be corrected at revision, so please note any specific errors here.

Reviewer #1: No

Reviewer #2: No

Response: The comment is well taken. By now our manuscript revised detail to have intelligible fashion and Standard English language presentation to make it clear for the readers.

---

5. Review Comments to the Author

Please use the space provided to explain your answers to the questions above. You may also include additional comments for the author, including concerns about dual publication, research ethics, or publication ethics. (Please upload your review as an attachment if it exceeds 20,000 characters)

Reviewer #1: his deals on an important and current issue, TB and drug resistance TB in Ethiopia. However, the manuscript poorly organized and need major revision. I try to mention some points on the main manuscript but many more left.

Response: Again, this comment is well taken. Based on each comment mentioned in the main manuscript document, we have done major revision and incorporated the suggested comments on the revised manuscript.

We authors glad to appreciate reviewer#1effort for his comment/concern and questions for unclear points found in main manuscript document.

Reviewer #2: Comments
The author picked an interesting topic that has public health importance but it is not a new topic. Such kind of work has important role in monitoring the distribution of MDR-TB in the study area. The study aimed to determine the magnitude of MDR-TB and associated factors as it is seen from the title of the manuscript and objectives mentioned in the abstract; however, in the background, results, and discussion there is a different scenario. There are concerns I encountered in this manuscript some of them are: what was the aim of this study?

Response: Thank you for your interest and stating the importance of the study as public health problem. By now, we have taken your comment and concern and define the objective of the study.

Why it vary from one section to the other? There are inconsistencies in this regard that can affect the results, discussion, and conclusion section.
Response: Again the comment is well taken. We have modified many things in the revised manuscript to keep the consistency in result, discussion and conclusion.

The other one is the sample size issue, unless it is resolved, I think it will affect the manuscript.

Response: With all due respect to the reviewer, we believe that this point is not correct and our sample size is calculated with single population proportion formula, and by now it is resolved in the revised document.

Authors are advised to carefully revise the manuscript. My comments are depicted below.

Response: Well come for your comments and thank you for investing your time on our manuscript.

I. General comments
1. Revise language

Response: The comment is well taken. By now our manuscript revised detail to have intelligible fashion and Standard English language presentation to make it clear for the readers.

2. Follow the scientific way of writing to name of bacteria
Response: We agree with this reviewer that very few bacteria were named non-scientifically, but now we have scientifically corrected their names.

3. There are several typographical errors that need revision for example in citing a reference in one place it is placed in [] in some other place it is presented as superscript. Sometimes reference which is not on the list is cited.

Response: Thank you for your constructive comment. We have corrected our manuscript citation and references based on the journal requirement.

4. Proper use of abbreviations: If abbreviations appear for the first time in the manuscript use the expanded form then after use the shortened form.

Response: Again the comment is well taken, and by now we have corrected our manuscript abbreviation.

5. I suggest the use of 'GeneXpert' instead of "X-pert MTB/RIF"

Response: Again the comment is well taken, and by now we have corrected our manuscript as per suggestion as GeneXpert.

II. Abstract
1. Several typos example "….from Jan to July 2019…" change Jan to January

Response: Thank you. We have corrected as per the comment.

2. The title does not reflect the objective (prevalence of MTB vs Prevalence of MDR; factors associated with the magnitude of MTB? Or MDR? ….justify or modify the title

Response: The comment is accepted. Based on the comment the title modified as "Magnitude of Mycobacterium tuberculosis, Drug resistance and associated factors among presumptive patients at St. Paul's Hospital Millennium Medical College, Addis Ababa, Ethiopia"

3. The background in the abstract does not reflect the title

Response: Based on the comment, now our background in the abstract section corrected to reflect the title.

4. Which method was used to detect MDR? The method in the abstract needs revision it should go with the title and objective (detection of MDR) what was the need for microscopy?

Response: The methods used for MDR detection were GeneXpert MTB/RIF assay and LJ culture media. We used Microscopy for cross checking, and now we have revised the abstract section.

5. What was the sample size of this study? 422? 436?

Response: It was proposed the minimum sample size using single population formula as 422. However, we used 436 participants in the actual data collection procedure (more than the determined sample size) during the study period.

6. Omit socio-demographic data, focus on the main findings: MDR, factors associated with MDR (this was not shown anywhere in the manuscript)

With all due respect to the reviewer, we believe that this point is not correct. Socio-demographic data is mandatory for magnitude of M. tuberculosis and to describe the statistical associations.

7. A statement "Out of the total participants, the overall confirmed Mycobacterium tuberculosis was through X-pert MTB/RIF assay and LJ culture media was 27 (6.2%), and three isolates were resistant for either INH or RIF drug, while two of them were MDR-TB based on line probe assays method" needs revision

Response: Based on the comment, we have revised the stated statement.

8. In a statement "Previous TB-contact history, patient weight loss, having pneumonia with chest X-ray finding, and CD4+ T-cells count 200-350/mm3 of blood were significantly associated predictors for MTB infection" does not go with the title and objective. As I understand the aim of the study is to determine factors associated with MDR-TB

Response: Based on the above comment, now we have revised the title to reflect exactly the objective and the associated factors.

9. What is the prevalence of MDR and its predictors, these two should be given emphasis in results and conclusion.

Response: Thank you very much. The prevalence of MDR-TB was 0.5%, and the predictors are not previous TB-contact history, patient weight loss, CD4+ T-cells count 200-350/mm3 of

blood were significantly associated predictors for MTB infection, and not for MDR-TB.

III. Background

1. The first sentence "…..caused by strains belonging to …" as strains is inappropriately used, modify as "…caused by Mycobacterium tuberculosis complex…"

i. In a statement "Globally, the estimated prevalence of MDR-TB was 3.3% in newly diagnosed patients in the WHO 2015 report. This was higher to 20% in patients with a history of anti-TB treatment(30)." Reference # 30 does not exist in the reference list.

Response: The comment is well taken and raised by the first reviewer, and by now it correctly cited.

2. Use updated information in the background: 2017 WHO report is old

Response: Thank you for your suggestion and our manuscript is revised using the updated WHO 2020 report.

3. Also use the updated information for Ethiopia instead of using 2005 report

Response: Thank you for your suggestion and our manuscript is revised using the updated 2018 national study.

4. The last sentence of the background is not in line with what has been mentioned in the title and abstract. They contradict each other, justify or revise

a. "Therefore, the goal of this study was to determine magnitude of Mycobacterium tuberculosis and its associated factors among TB- presumptive patients referred to St. Paul's Hospital Millennium Medical College, Addis Ababa, Ethiopia."

b. "Therefore, the goal of this study was to determine magnitude of Multi Drug Mycobacterium tuberculosis (MDR-TB) and its associated factors among TB- presumptive patients at St. Paul's Hospital Millennium Medical College, Addis Ababa, Ethiopia"

Response: Thank you for your comment and now we have revised as suggested in the option a.

5. Use another word instead of the "the goal of this study…"

Response: We changed the goal of this study by the aim of this study.

IV. Materials and Methods

1. In the study area, mention the TB clinic (patient flow, service…etc)

Response: The comment is taken and described in the revised document.

2. What does 'Jan' stands for? Is it January?

Response: Yes to mean that January. Now we have corrected it as January in the revised manuscript.

3. Study variables should go with the title and objective of the study: MDR-TB and predictors of MDR-TB

Response: Now we have corrected the title and objective of the study, and both dependent and independent variables are in line with the revised title and objectives.

4. Determination of sample size is not clear. If p=23% (reference is not given, it is not clear whether it is the prevalence of TB or MDR), d=5% is used the sample size will be 272 + 10% (n=299) which is different from what is mentioned in the manuscript (n=422). Moreover, a sample size different from these two was used in the result section (n=436).

Response: The comment is accepted. Now we have revised that our sample size calculation using single population proportion formula, and the minimum sample size for the study was 422 participants. However that, during the actual collection of the study period we have collected data from 436 participants. (I think it is expected to collect more data from the pre-determined sample to be more representative).

5. Mention sampling technique; non-probability sampling technique (convenient)? Systematic random sampling technique? Random sampling technique?

Response: We have already mentioned our sampling technique as "consecutive sampling technique was used to select the study population", which is usually applicable to collect data from health institution as probability sampling techniques, since it is difficult to apply Systematic random sampling technique or random sampling technique. This sampling technique is considered as probability sampling method in most literatures.

6. Detail is needed on data collection procedure: how the questionnaire was validated? Move clinical specimen collection to the laboratory section.

Response: It is already stated as "the questionnaire was pre-tested and proper training prior to the actual data collection was given for data collectors. The necessary adjustments were made after the pre-test", in the data quality assurance section and collection procedure is well revised and categorized as: Collection using questionnaire, laboratory collection procedures, using GeneXpert, Microscopic examination and culturing method.

7. How the sample was collected from children who are unable to provide sputum? I think it is difficult to obtain sputum from children, how did you handle it?

Response: Thank you for your concern. We were describing that taking from participants of 2-4 ml of clinical sputum samples, pus, lymph node aspirate or peritoneal, pleural fluid and gastric aspirate. Sputum sample was taken usually from children older than 10 years who can produce sputum. We used Gastric aspirate or induced sputum by physician support for children unable to provide sputum by coughing.

8. In Laboratory procedures section mention what was done to identify MTB, MDR (microscopy?, culture, GeneXpert) instead of description or performance of the methods.

Response: The comment is accepted, and the procedures are mentioned in the revised manuscript.

9. I am not sure if the use of trademark is allowed by the journal (®), check it out.

Response: The comment is accepted and removed from the revised document.

10. Data quality assurance: mention how the quality of data was maintained both for socio-demographic, clinical characteristics, and Laboratory data

Response: Thank you. By now we have clearly mentioned.

11. Data analysis and interpretation: what does data editing mean? You should have considered multivariate analysis.

Response: The comment is taken and removed unnecessary words, and regarding to the multivariable analysis consideration, we agree with the reviewer the importance of multivariate analysis in terms of controlling the compfounders. However that, no socio-demographic factors was a candidate (p<0.25) for multivariable analysis in the table 3, and for few variable that were associated in the table 4, unfortunately we did not do analysis for multivariable analysis.

12. In a statement "…to assess the association between different factors.." what are these factors?

Response: Again the comment is taken and corrected as " to assess the association between TB and risk factors"

13. Ethical consideration: correct "…Department of Medical Laboratory Sciences" as "…….Science" check if it is school or department. Does the department of Medical Laboratory Science have IRB; most of the time IRB is at the college level.

Response: It is corrected as Science. Yes, it is department not the school, and has its own IRB.

14. In the sentence "…then submitted to laboratory department" which laboratory department? And why?

Response: I think this is clear, Laboratory department means the actual study area which data was collected, it is St. Paul's Hospital Millennium Medical College, Microbiology laboratory department, while the study was approved by Department of Medical Laboratory Science, Addis Ababa, University.

15. Did you obtain assent for children?

Response: Yes. Now we have stated in the ethics section of the revised manuscript.

16. Include operational definition for MDR, presumptive TB, and presumptive MDR-TB

Response: Thank you for your constructive comments. By now we have included our operation definition for MDR TB, presumptive TB, and presumptive MDR-TB

V. Results

1. The whole result is based on the sample size (436vs422vs299) which is different from the one mentioned in the method section (where do 436 come from?).

Response: The minimum sample size calculated or predetermined was 422, however the actual study participants were 436, and it is advised to use large sample size in any study to be more representative of the study result.

2. "…had monthly income 100-1000 Ethiopian Birr, table 1." May be re-written as "…………..Ethiopian Birr (Table 1)." Do the same wherever it applies.

Response: The comment is taken and modified based on the comment.

3. In a clinical data: “About 422 (96.8%) of the participants were presumptive TB..” how is this different from “From the total 374 (85.8%) were suspected for pulmonary tuberculosis and 62 (14.2%) were suspected for extra-pulmonary tuberculosis”

Response: No difference in the interpretation except different wording. By now it is corrected be consistent as presumptive TB.

4. Why most of the study participants are HIV positive? What kind of impact does it have on your study?

Response: Of the total 130 (30%) were HIV positive participants, of which104 (81%) were on anti-HIV treatment. These may have an impact to be infected with MTB or the chance to develop TB or MDR-TB.

5. Rename subheading ‘Bivariate analysis’ as ‘Factors associated with MDR-TB’

Response: The comment is accepted and modified as reviewer’s wish bivariate analysis to factor associated to MTB.

6. As mentioned above, the analysis does not go with title and objective and also multivariate analysis was not conducted.

Response: The comment was taken from the previous concern and suggestion. By now the title is modified as “Magnitude of Mycobacterium tuberculosis, Drug resistance and associated factors among presumptive patients at St. Paul’s Hospital Millennium Medical College, Addis Ababa, Ethiopia”, which is in line with the objective and the analysis of the manuscript.

VI. Discussion

1. Because of some points mentioned above, the discussion is not in order: it should follow the main findings of the study: should discuss the magnitude of MDR that is followed by risk factors. Discussion can be re-written after addressing the comments given above.

Response: The comment is taken and our manuscript is revised based on the comment.

2. Some types, page 11: “Mycobacterium Tuberculosis” correct as Mycobacterium tuberculosis, ‘East Gojjam zone, northwest Ethiopia,19” & “eastern Ethiopia (14.2%)18 “ references are in superscript put them in []

Response: Thank you. By now it is correctly cited as [18].

3. In a statement “The possible reason for the difference might be associated with the variation of the diagnostic methods we used, for example in our cases we used sputum sedimentation concentration technique for microscopic smear examination, Gene X-pert assay and finally LJ culture for”confirmation whereas, a single diagnostic tool used in the previous study like;stained by ZiehlNeelsen staining and examined by Microscopy in the case of Metehara [18]” in the

method section you did not mention that you have used sputum sedimentation concentration technique. Moreover if you have used all this the fining should be higher.

Response: Thank you very much again. Now it is clearly stated in the method section of the revised manuscript about sputum sedimentation concentration technique. In addition the overall result of the study will be lower because of minimizing of false positive using parallel different diagnostic and confirmatory methods.

VII. Conclusions
1. Conclusion needs revision

Response: The constructive comment is well taken, and we have revised the conclusion section.

VIII. References and Tables
2. Reference writing style is not uniform (#1, 7, 8, 16, 18,), Add URL for refer #3,

Response: By now, we have made. All the references are uniform which have URL and PubMed citation format with their own doi and PMID in the revised manuscript.

3. Tables: the labeling of table 1 is not correct (it is not for MDR?), you may omit Colum 3 and 4 (MTB present & MTB absent), the age category could have been reduced. Replace 'illiterate' with no formal education, monthly income category is not reasonable.

Response: We have revised the table title and its content as per the comment.

The same comments apply for table 2 (correct labeling of the table and omit Colum 3 &4.

Response: We have revised the table title and its content as per the comment.

Expand all abbreviations mentioned in the table under the tables. Re-write labeling of table 3 &4 analysis of what with what (MDR with socio or TB with socio) please check your title of manuscript and objective.

Response: We have revised the table title and its content as per the comment.

And also consider multivariate analysis for variables with p<0.25. In the tables include both frequency and percentages in each cell.

Response: Regarding to the multivariable analysis consideration, we agree with the reviewer the importance of multivariate analysis in terms of controlling the compfounders. However that, no socio-demographic factors was a candidate (p<0.25) for multivariable analysis from table 3, and for few variable that were associated in the table 4, unfortunately we did not do analysis for multivariable analysis.

---

6. PLOS authors have the option to publish the peer review history of their article (what does this mean?). If published, this will include your full peer review and any attached files.

If you choose “no”, your identity will remain anonymous but your review may still be made public.

Do you want your identity to be public for this peer review? For information about this choice, including consent withdrawal, please see our Privacy Policy.

Reviewer #1: No

Reviewer #2: No

Response: No problem.

[NOTE: If reviewer comments were submitted as an attachment file, they will be attached to this email and accessible via the submission site. Please log into your account, locate the manuscript record, and check for the action link "View Attachments". If this link does not appear, there are no attachment files.]

While revising your submission, please upload your figure files to the Preflight Analysis and Conversion Engine (PACE) digital diagnostic tool, https://pacev2.apexcovantage.com/. PACE helps ensure that figures meet PLOS requirements. To use PACE, you must first register as a user. Registration is free. Then, login and navigate to the UPLOAD tab, where you will find detailed instructions on how to use the tool. If you encounter any issues or have any questions when using PACE, please email PLOS at figures@plos.org. Please note that Supporting Information files do not need this step.

In compliance with data protection regulations, you may request that we remove your personal registration details at any time. (Remove my information/details). Please contact the publication office if you have any questions.
