# Supplementary material for: Magnitude of Mycobacterium tuberculosis, drug resistance and associated factors among presumptive tuberculosis patients at St. Paul’s Hospital Millennium Medical College, Addis Ababa, Ethiopia
Source: PLoS One. 2022 Aug 1;17(8):e0272459. doi: 10.1371/journal.pone.0272459 (PMC9342779; doi:10.1371/journal.pone.0272459)
Supplement: Attachment — Submitted filename: Comments.docx [file pone.0272459.s004.docx]

All my comments are addressed. I don’t have further comments.
