# Supplementary material for: Magnitude of Mycobacterium tuberculosis, drug resistance and associated factors among presumptive tuberculosis patients at St. Paul’s Hospital Millennium Medical College, Addis Ababa, Ethiopia
Source: PLoS One. 2022 Aug 1;17(8):e0272459. doi: 10.1371/journal.pone.0272459 (PMC9342779; doi:10.1371/journal.pone.0272459)
Supplement: Attachment — Submitted filename: Response Reviewer_2.docx [file pone.0272459.s006.docx]

**PONE-D-22-03876R1**

Magnitude of Mycobacterium tuberculosis, drug resistance and associated factors among presumptive patients at St. Paul’s Hospital Millennium Medical College, Addis Ababa, Ethiopia

PLOS ONE

Dear Dr. Yeshanew,

Thank you for submitting your manuscript to PLOS ONE. After careful consideration, we feel that it has merit but does not fully meet PLOS ONE’s publication criteria as it currently stands. Therefore, we invite you to submit a revised version of the manuscript that addresses the points raised during the review process.

Please submit your revised manuscript. If you will need significantly more time to complete your revisions, please reply to this message or contact the journal office at [plosone@plos.org](mailto:plosone@plos.org). When you're ready to submit your revision, log on to <https://www.editorialmanager.com/pone/> and select the 'Submissions Needing Revision' folder to locate your manuscript file.

Please include the following items when submitting your revised manuscript:

- A rebuttal letter that responds to each point raised by the academic editor and reviewer(s). You should upload this letter as a separate file labeled 'Response to Reviewers'.
- A marked-up copy of your manuscript that highlights changes made to the original version. You should upload this as a separate file labeled 'Revised Manuscript with Track Changes'.
- An unmarked version of your revised paper without tracked changes. You should upload this as a separate file labeled 'Manuscript'.

If you would like to make changes to your financial disclosure, please include your updated statement in your cover letter. Guidelines for resubmitting your figure files are available below the reviewer comments at the end of this letter.

If applicable, we recommend that you deposit your laboratory protocols in [protocols.io](http://protocols.io) to enhance the reproducibility of your results. Protocols.io assigns your protocol its own identifier (DOI) so that it can be cited independently in the future. For instructions see: <https://journals.plos.org/plosone/s/submission-guidelines#loc-laboratory-protocols>. Additionally, PLOS ONE offers an option for publishing peer-reviewed Lab Protocol articles, which describe protocols hosted on [protocols.io](http://protocols.io). Read more information on sharing protocols at <https://plos.org/protocols?utm_medium=editorial-email&utm_source=authorletters&utm_campaign=protocols>.

We look forward to receiving your revised manuscript.

Kind regards,

Frederick Quinn
Academic Editor
PLOS ONE

***Response: Dear Frederick Quinn, Academic Editor, PLOS ONE***

***We authors would like to thank for your invitation to re-submit our revised version of the manuscript, “PONE-D-22-03876R1, Magnitude of Mycobacterium tuberculosis, drug resistance and associated factors among presumptive patients at St. Paul’s Hospital Millennium Medical College, Addis Ababa, Ethiopia”, that addresses the points raised during the review process.***

***We included a point-by-point response within the 'Response to Reviewers' box in the submission system and modifications are highlighted in the track changes of the original manuscript, and we uploaded also the clean or unmarked version of our revised paper without tracked changes.***

**Journal Requirements:**
Please review your reference list to ensure that it is complete and correct. If you have cited papers that have been retracted, please include the rationale for doing so in the manuscript text, or remove these references and replace them with relevant current references. Any changes to the reference list should be mentioned in the rebuttal letter that accompanies your revised manuscript. If you need to cite a retracted article, indicate the article’s retracted status in the References list and also include a citation and full reference for the retraction notice.

[Note: HTML markup is below. Please do not edit.]

***Response: Thank you for your suggestion. We have ensured you that our reference list is completed, and we did not cite retracted papers.***

**Reviewers' comments:**

Reviewer's Responses to Questions

**Comments to the Author**

1. If the authors have adequately addressed your comments raised in a previous round of review and you feel that this manuscript is now acceptable for publication, you may indicate that here to bypass the “Comments to the Author” section, enter your conflict of interest statement in the “Confidential to Editor” section, and submit your "Accept" recommendation.

Reviewer #1: (No Response)

Reviewer #2: All comments have been addressed

***Response: We thank you all reviewers for their comments and concerns.***

2. Is the manuscript technically sound, and do the data support the conclusions?

The manuscript must describe a technically sound piece of scientific research with data that supports the conclusions. Experiments must have been conducted rigorously, with appropriate controls, replication, and sample sizes. The conclusions must be drawn appropriately based on the data presented.

Reviewer #1: Partly

Reviewer #2: Yes

***Response: We thank you all reviewers for their satisfaction about our data, their beliefs on our conclusions which was drawn appropriately based on our data presentation***

3. Has the statistical analysis been performed appropriately and rigorously?

Reviewer #1: Yes

Reviewer #2: Yes

***Response: We thank both reviewers for their beliefs on our data appropriate analyzed.***

4. Have the authors made all data underlying the findings in their manuscript fully available?

The [PLOS Data policy](http://www.plosone.org/static/policies.action#sharing) requires authors to make all data underlying the findings described in their manuscript fully available without restriction, with rare exception (please refer to the Data Availability Statement in the manuscript PDF file). The data should be provided as part of the manuscript or its supporting information, or deposited to a public repository. For example, in addition to summary statistics, the data points behind means, medians and variance measures should be available. If there are restrictions on publicly sharing data—e.g. participant privacy or use of data from a third party—those must be specified.

Reviewer #1: No

Reviewer #2: Yes

***Response: Thank you for reviewer #1 concern. However, it may be due to personal difference, and we authors believed that all data underlying the findings in our manuscript are fully available.***

5. Is the manuscript presented in an intelligible fashion and written in standard English?

PLOS ONE does not copyedit accepted manuscripts, so the language in submitted articles must be clear, correct, and unambiguous. Any typographical or grammatical errors should be corrected at revision, so please note any specific errors here.

Reviewer #1: No

Reviewer #2: Yes

***Response: The comment is well taken. By now our manuscript revised detail to have intelligible fashion and Standard English language presentation, to make it clear for the readers for the second time.***

6. Review Comments to the Author

Please use the space provided to explain your answers to the questions above. You may also include additional comments for the author, including concerns about dual publication, research ethics, or publication ethics. (Please upload your review as an attachment if it exceeds 20,000 characters)

Reviewer #1: Despite the manuscript generally has showed good improvement, but it has many flaw for the publication. It need additional work to improve it. I tried to identity some of them and tried to highlight on the main manuscript.

Reviewer #2: All my comments are addressed. I don't have additional comments. The manuscript is improved considerably.

***Response: The comment is well taken. We thank reviewer #2 for your satisfaction on our revised manuscript. However, for reviewer #1, also we would like to acknowledge for investing his time to see our manuscript and criticize well for the second time. By now our manuscript is well revised for the second time, and we believe that its great improvement.***

7. PLOS authors have the option to publish the peer review history of their article ([what does this mean?](https://journals.plos.org/plosone/s/editorial-and-peer-review-process#loc-peer-review-history)). If published, this will include your full peer review and any attached files.


If you choose “no”, your identity will remain anonymous but your review may still be made public.


**Do you want your identity to be public for this peer review?** For information about this choice, including consent withdrawal, please see our [Privacy Policy](https://www.plos.org/privacy-policy).

Reviewer #1: No

Reviewer #2: No

***Response: No problem. We would like to thank both two reviewers and editors for their constructive comments, suggestion and concerns to improve our manuscript for intellectual content.***

***Thank you.***
